# Alternative promoters and splicing create multiple functionally distinct isoforms of oestrogen receptor alpha in breast cancer and healthy tissues

**DOI:** 10.1002/cam4.6508

**Published:** 2023-09-07

**Authors:** Carlos Enrique Balcazar Lopez, Juliane Albrecht, Völundur Hafstað, Cornelia Börjesson Freitag, Johan Vallon‐Christersson, Cristian Bellodi, Helena Persson

**Affiliations:** ^1^ Department of Clinical Sciences Lund, Oncology Lund University Cancer Centre Lund Sweden; ^2^ Division of Molecular Hematology, Department of Laboratory Medicine, Lund Stem Cell Center, Faculty of Medicine Lund University Lund Sweden

**Keywords:** alternative splicing, breast cancer, isoforms, oestrogen receptor

## Abstract

**Background:**

Oestrogen receptor alpha (ER) is involved in cell growth and proliferation and functions as a transcription factor, a transcriptional coregulator, and in cytoplasmic signalling. It affects, for example, bone, endometrium, ovaries and mammary epithelium. It is a key biomarker in clinical management of breast cancer, where it is used as a prognostic and treatment‐predictive factor, and a therapeutical target. Several ER isoforms have been described, but transcript annotation in public databases is incomplete and inconsistent, and functional differences are not well understood.

**Methods:**

We have analysed short‐ and long‐read RNA sequencing data from breast tumours, breast cancer cell lines, and normal tissues to create a comprehensive annotation of ER transcripts and combined it with experimental studies of full‐length protein and six alternative isoforms.

**Results:**

The isoforms have varying transcription factor activity, subcellular localisation, and response to the ER‐targeting drugs tamoxifen and fulvestrant. Antibodies differ in ability to detect alternative isoforms, which raises concerns for the interpretation of ER‐status in routine pathology.

**Conclusions:**

Future work should investigate the effects of alternative isoforms on patient survival and therapy response. An accurate annotation of ER isoforms will aid in interpretation of clinical data and inform functional studies to improve our understanding of the ER in health and disease.

## Graphical abstract

A comprehensive transcript annotation for the breast cancer therapy target oestrogen receptor alpha including alternative first, internal and last exons, as well as splice donor and acceptor sites. Detailed functional studies compare six alternative isoforms to the full‐length protein.

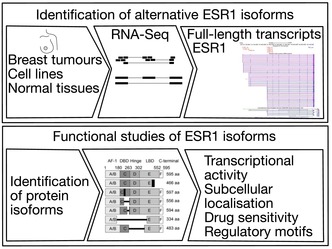

## 1 INTRODUCTION

The availability of the human genome sequence and high‐throughput sequencing methods provide rich material for research, but both experimental and bioinformatic studies need accurate transcript models. Projects such as GENCODE and RefSeq aim to provide comprehensive annotation through a combination of manual and automated curation.^1^, ^2^ However, the quality of the transcript models for a given gene strongly depend on the available sequencing data and the set of transcripts can change dramatically between database releases. Here, we have performed an in‐depth study of mRNA isoforms of the oestrogen receptor alpha (ER, official gene symbol: *ESR1*). The ER is primarily expressed in mammary glands and the female reproductive tract but is also present in a wide range of tissues such as liver and adipose tissue.^3^, ^4^ It is a transcription factor belonging to the family of nuclear hormone receptors and is, together with oestrogen receptor beta (*ESR2*) and the G‐protein coupled receptor GPER1, one of three known receptors in humans for oestrogens such as estrone, 17‐beta‐oestradiol (E2) and estriol.

The ER is distributed between nucleus and cytoplasm and interacts with several heatshock proteins of the HSP70 and HSP90 families.^5^ Upon ligand‐binding, it dimerises and binds to oestrogen response elements (EREs) to activate transcription. The minimal ERE is a palindromic DNA motif with the consensus sequence 5′‐GGTCAnnnTGACC‐3′.^6^ Activity of the ER depends on co‐activators such FOXA1 that change the chromatin state.^7^, ^8^, ^9^ It can also act as a co‐activator itself together with the AP1 or SP1 transcription factors, or by ligand‐independent transcriptional activation upon phosphorylation of critical residues including serine 118.^10^, ^11^, ^12^ Additional roles for the ER include chromatin remodelling through interaction with the histone H3 lysine 9 demethylase KDM4B^13^, ^14^ and rapid, so‐called non‐genomic effects that have been described for a subpopulation of ER located at the plasma membrane.^15^, ^16^ Recently, it was also reported that the ER is a non‐canonical RNA‐binding protein that acts in post‐transcriptional regulation of gene expression.^17^

In breast cancer, the ER is both a functionally important oncogene and a key clinical biomarker that is used as a (positive) prognostic and a treatment‐predictive factor. Approximately 75% of breast tumours are positive for ER expression by immunohistochemistry.^18^ Patients with ER‐positive tumours can receive adjuvant treatment with tamoxifen, a drug that has mixed antagonist and agonist properties and that is referred to as a selective oestrogen receptor modulator (SERM). Other treatment options include aromatase inhibitors that block oestrogen synthesis and, in recurrent disease, fulvestrant, a selective oestrogen receptor degrader (SERD). Several alternative protein isoforms that exist in parallel with the 66 kDa full‐length ER and differ in function have been described. These include isoforms of 46, 36 and 30 kDa, as well as multiple isoforms that involve cryptic alternative exons.^19^, ^20^, ^21^, ^22^

Complete annotation of all mRNA isoforms of the ER would help us better understand the regulation and complex biological effects of this transcription factor that has roles in both healthy and diseased tissues. It could also lead to improved clinical application of the ER as a prognostic and treatment‐predictive biomarker and a target for therapy. Unfortunately, the current transcript annotation in GENCODE and RefSeq has substantial differences, does not include some isoforms described in older literature and lacks both new exons and alternative splicing events that were readily detectable in our RNA sequencing (RNA‐Seq) data. We have therefore combined in‐depth analysis of transcriptional events in short‐read RNA‐Seq data with whole‐isoform detection in long‐read RNA‐Seq data to provide what is likely to be the most comprehensive annotation of the *ESR1* locus to date. We have also performed functional studies to compare six alternative protein isoforms with the full‐length receptor. The experimental results show marked differences in transcription factor activity, subcellular localisation and sensitivity to the SERD fulvestrant. The ability to detect these isoforms using N‐ or C‐terminal antibodies varies depending on which protein domains are included, a finding of potential clinical importance. An implication of the findings presented here is that analyses which are based on incomplete or different sets of annotated mRNA isoforms can produce confusing or even misleading results. This applies for example to RNA‐Seq data analysis, primer design for real‐time reverse transcription polymerase chain reaction (real‐time RT‐PCR) and interpretation of results from immunohistochemistry (IHC), western blots and functional studies. Our results provide a comprehensive view of alternative mRNA isoforms of the ER that can aid interpretation of clinical data and inform functional studies to improve our understanding of the ER in health and disease.

## 2 MATERIALS AND METHODS

Please see Data S2 for additional details.

### 2.1 Patient data and short‐read RNA‐Seq

The SCAN‐B study was conducted in accordance with the Declaration of Helsinki and has been approved by the Regional Ethical Review Board of Lund (2007/155, 2009/658, 2009/659, 2014/8), the county governmental biobank centre and the Swedish Data Inspection group (364‐2010). Written information was given by trained health professionals, and all patients provided written informed consent. Library preparation and sequencing for RNA‐Seq are described in.^23^

### 2.2 Short‐read RNA‐Seq data analysis

Sequence data had been aligned against GENCODE V27 and hg38 using HISAT2 2.1.0.^24^ Splice junction detection was done on BAM files and included known exons and splice junctions from GENCODE V36 and RefSeq release 109.20210226. The merged splice junction set was filtered and used together with the sequencing read depth to guide manual curation of exons and splice junctions.

### 2.3 Long‐read RNA‐Seq

RNA was extracted from tumour samples provided by the SCAN‐B tissue bank and the BT‐474, MCF7 and T47D cell lines. The FirstChoice Human Total RNA Survey Panel with normal tissue RNA was purchased from Ambion/Thermo Fisher Scientific. Primers for RT‐PCR were designed to amplify full‐length transcripts from seven different locations in first exons and are included in Table S1. PCR products were pooled in equimolar amounts per sample before library preparation for Oxford Nanopore Technologies sequencing. Reads were aligned using minimap2 version 2.17, and full‐length reads were assigned to individual amplicons through comparison with the coordinates of forward and reverse primers used for RT‐PCR. Transcripts were filtered and divided into three tiers depending on the available splice junction support.

### 2.4 Isoform and luciferase reporter cloning

The coding sequences of full‐length ER and six selected alternative isoforms were created in the pEGFP‐C1 vector (Clontech) from DNA fragments in Table S2. The pmirGLO‐3xERE reporter was constructed from the 3xERE‐containing region of the 3xERE‐TATA‐luc plasmid^25^ (Addgene) cloned in the pmirGLO vector (Promega) that had been engineered to introduce a multiple cloning site upstream of the transcription start site. The pmirGLO human C3 promoter reporter contains a weak ERE and an AP1 binding site (−307/+58, described in^26^) and was constructed by amplification of a fragment from a plasmid that was a kind gift from Gilles Flouriot, Research Institute for Environmental and Occupational Health, Rennes, France.^27^ All inserts were verified by Sanger sequencing. Primer sequences are shown in Table S1.

### 2.5 Cell culture

Breast cancer cell lines were obtained from American Type Culture Collection (ATCC) and HepG2 from LGC Standards GmbH and cultured at 37°C, 5% CO_2_ in a humidified atmosphere. Cell line identity was authenticated by STR profiling, and all cells were verified to be mycoplasma‐free (Eurofins Genomics). HepG2 (RRID: CVCL_0027) and MCF7 (RRID: CVCL_0031) were cultured in Dulbecco's Modified Eagle's Medium (DMEM) High Glucose with 10% fetal bovine serum (FBS), both from Cytiva/HyClone. Insulin (Thermo Fisher Scientific) was added to 10 μg/mL for MCF7. BT‐474 (CVCL_0179) and T47D (CVCL_0553) were cultured in RPMI‐1640 (Cytiva/HyClone) with 10% FBS (Cytiva/HyClone). For experiments involving stimulation with ER ligands, cells were seeded in phenol red‐free DMEM (Cytiva/HyClone) with 2.5% charcoal‐stripped, dextran‐treated FBS (CSS‐FBS, Cytiva/HyClone).

### 2.6 Western blotting

Primary antibodies used in western blotting were C‐terminal anti‐ER antibody (sc‐543, Santa Cruz Biotechnology, 1:500), N‐terminal anti‐ER antibody (HPA000449, Atlas antibodies, 1:250), anti‐α‐tubulin antibody (ab7291, abcam, 1:5000) and the anti‐Lamin B2 antibody (ab8983, abcam, 1:1000). Secondary antibodies were HRP‐conjugated goat anti‐rabbit (31,460, Invitrogen/Thermo Fisher Scientific) or anti‐mouse (31,430, Invitrogen/Thermo Fisher Scientific) secondary antibodies, both diluted 1:10,000.

### 2.7 Luciferase assays

HepG2 and MCF7 cells were transfected with empty pEGFP‐C1 and/or ER isoforms cloned in pEGFP‐C1. The medium was replaced after 24 h with fresh phenol red‐free DMEM with 2.5% CSS‐FBS containing either 0.1% ethanol (vehicle control), 10 nM E2 (Sigma‐Aldrich/Merck), 10 nM E2 and 100 nM 4‐hydroxytamoxifen (4OHT, Selleck Chemicals), or 100 nM 4OHT. Cells were lysed after 24 h and analysed with the Dual‐Luciferase Reporter Assay System (Promega).

### 2.8 Subcellular fractionation

At 24 h after transfection of HepG2 cells, the medium was replaced with fresh phenol red‐free DMEM with 2.5% CSS‐FBS containing either 0.1% ethanol (vehicle control) or 10 nM E2. Cells were incubated at 37°C for 30 min before scraping and subcellular fractionation. Total lysate and subcellular fractions were analysed by western blotting.

### 2.9 Immunofluorescence

Cells were seeded in Lab‐Tek II Chamber Slides (Thermo Scientific/Nunc) and transfected with ER isoforms cloned in pEGFP‐C1. At 24 h after transfection, cells were treated with vehicle control or 10 nM E2 for 30 min at 37°C before staining for immunofluorescence. Primary antibodies were rabbit anti‐ESR1, clone EP1, Dako, 1:100 and mouse anti‐actin, clone C4, MP Biomedicals, 1:500. Secondary antibodies were AF488‐conjugated goat anti‐rabbit and AF647‐conjugated goat anti‐mouse diluted 1:1000, ThermoFisher/Invitrogen.

### 2.10 ER degradation

At 24 h after transfection of HepG2 cells, the medium was replaced with fresh phenol red‐free DMEM with 2.5% CSS‐FBS containing either 0.01% DMSO (vehicle control, Thermo Fisher Scientific) or 1 μM fulvestrant (Selleck Chemicals). Cells were incubated for 24 h before lysis and analysis by western blotting.

### 2.11 Polysome fractionation and isoform‐specific real‐time RT‐PCR

Polysome fractionation was performed as described in^28^ with minor modifications. TRI reagent LS (Sigma‐Aldrich) was added to each fraction for RNA extraction with the Direct‐Zol RNA Microprep kit (Zymo Research) including on‐column DNase treatment. Fractions containing polysome RNA were pooled after RNA extraction and used for cDNA synthesis using RevertAid H Minus reverse transcriptase (Thermo Fisher Scientific) with anchored oligo(dT) primers (dT_20_VN). Primer sequences for real‐time RT‐PCR on the on the CFX96 real‐time PCR detection system with iTaq Universal SYBR Green Supermix (Bio‐Rad) are included in Table S1.

### 2.12 Analysis of regulation of promoters and alternative splicing

Transcription factor data were from the UniBind robust set of transcription factor ‐ DNA interactions.^29^ For promoter methylation analysis, *ESR1* expression and beta values were obtained for breast tumours (TCGA‐BRCA) from The Cancer Genome Atlas (TCGA).^30^ For conservation analysis, phyloP^31^ scores for the *ESR1* locus came from the UCSC Table Browser.^32^ The MaxEntScan^33^ web server was used for the splice site calculations (http://hollywood.mit.edu/burgelab/maxent/Xmaxentscan_scoreseq.html and http://hollywood.mit.edu/burgelab/maxent/Xmaxentscan_scoreseq_acc.html).

### 2.13 Statistical analysis and data visualisation

Statistical analyses and plotting were done in R version 4.1.0 and Microsoft Excel. Survival analysis was performed in R using the survival and survminer packages. GraphPad Prism version 9 was used to plot the dose–response curve and calculate half maximal effective concentration (EC_50_) values. Unless otherwise stated, statistical tests used Student's *t‐*test.

## 3 RESULTS

### 3.1 Creating an extended map of ER exons and splice junctions

To create a comprehensive map of exons and splice junctions for the *ESR1* locus, we analysed RNA‐Seq data for 3478 breast tumours (Table S3) in combination with known splice junctions from GENCODE and RefSeq. The merged splice junction set was filtered and used together with the sequencing read depth to guide manual curation of exons and splice junctions. An overview of the annotation pipeline is shown in Figure S1, and the resulting annotation is included in Table S4. The new transcript map of *ESR1* contains many novel first, internal and last exons, as well as splice junctions and splice site combinations (Figure S2). It can provide rich material for experimental studies and improve our understanding of transcriptional regulation and the functions of the ER.

### 3.2 Expression patterns in breast cancer

**FIGURE 1:**
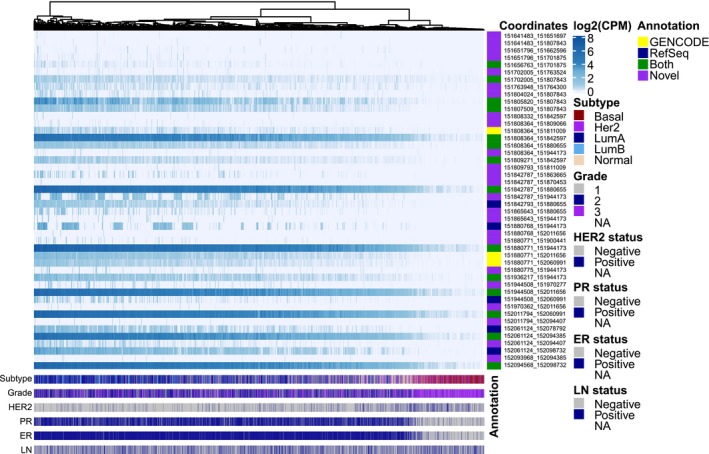
Heatmap illustrating the expression of splice junctions used in *ESR1* transcripts among 3478 breast tumours with junctions sorted by increasing genomic coordinates from top to bottom. Only junctions with an expression of at least 2 counts per million reads (cpm) in at least 2 samples are shown. Tumours are labelled according to molecular subtype, grade and status for ER, progesterone receptor (PR), the tyrosine kinase receptor ERBB2 (also known as HER2) and positive lymph nodes at surgery (LN). ER, PR and HER2 status were defined by immunohistochemistry. Exons are marked in green if they are annotated in both GENCODE and RefSeq databases, yellow for GENCODE only, blue for RefSeq only and purple for novel exons.

We next investigated the expression of the extended set of exons and splice junctions in the breast tumour cohort. Figure 1 shows samples clustered by the expression of splice junctions sorted by their genomic coordinates together with clinical tumour annotation. A corresponding heatmap for exons is shown in Figure S3. As expected, most splice junctions show a gradient of increasing expression from ER‐negative and basal‐like tumours to ER‐positive tumours of the luminal A and B subtypes. ER status was here defined by immunohistochemistry with nuclear staining in ≥10% of tumour cells as cut‐off for ER‐positivity. Interestingly, some alternative junctions have an expression pattern that is un‐ or anticorrelated with the canonical ER splice sites (Figure S4).

We then analysed differential expression of exons and splice junctions between groups of tumours (Figure S5). Percent spliced‐in (PSI) scores measure the inclusion rate of each exon or splice site among all reads covering the event (see Data S2 for details). Splicing events with significant differences included an alternative donor site in exon 2 of the canonical full‐length transcript leads to insertion of two amino acids in the DNA‐binding domain (DBD, see cds11 in Table 1), an alternative donor site in exon 3 that leads to deletion of a single amino acid in the DBD (see cds16 in Table 1) and exclusion of exon 7, a known cassette exon^34^ that leads to a frame‐shift and produces a truncated protein (see cds1 in Table 1).

**TABLE 1:** Characteristics of protein isoforms selected for functional validation, including coding sequence differences according to HGVS nomenclature, number of amino acids, matches to annotated transcripts and the underlying alternative splicing events.

Isoform	Alteration	Amino acids	Annotated transcripts	Transcriptional event
cds1	Gly457Valfs*10	466	NM_001385570, NM_001385571, NM_001385572	Skipping exon 7
cds11	Gly215_His216insAsnArg	597	NM_001291230	Alternative donor exon 2
cds13	His216_Gly254del	556	NA	Skipping exon 3
cds16	Gly254del	594	NM_001291241	Alternative donor exon 3
cds38	Pro152_Arg412del	334	ENST00000406599.5	Skipping exons 2–5
cds60	Gly254_Pro365del	483	NA	Skipping exon 4

We also calculated analogous scores for first and last exons as the fraction of the expression of all first or last exons, respectively (Figure S6). Exons with significant differences included an upstream first exon, the main first exon and an alternative last exon located between exons 7 and 8 of the canonical full‐length isoform that encodes a previously reported isoform with a truncated ligand‐binding domain (LBD).^20^

### 3.3 Determining full‐length transcript isoforms

The high read depth and quality of Illumina data are excellent for exon and splice site annotation, but even with paired‐end data it is only possible to connect a few exons at most. To assemble our novel features into full‐length transcripts we performed Oxford Nanopore Technologies (ONT) long‐read sequencing of RT‐PCR products. Forward primers were designed in seven different first exons (one of them novel) with matching reverse primers located in the main last exon to capture whole transcripts (products shown in Figure S7). Sequencing included eight breast tumours, three ER‐positive breast cancer cell lines (BT‐474, MCF7 and T47D) and 12 normal tissues (adipose, bladder, breast, cervix, kidney, liver, lung, ovary, prostate, skeletal muscle, spleen, testis). The analysis pipeline is illustrated in Figure S8. Aligned full‐length reads spanning from forward to reverse primer were compared to the curated sets of exons and splice junctions and transcripts were divided into three tiers depending on the available support: Tier 1 isoforms only have exon ends present in the curated set of exons from SCAN‐B breast tumours, GENCODE and RefSeq, tier 2 isoforms have one or more exon ends that are only present in the unfiltered SCAN‐B junction set and isoforms in tier 3 have one or more exon ends that have not been supported by new or existing annotation. Exon and junction support from long‐read sequencing has been included in Table S4.

**FIGURE 2:**
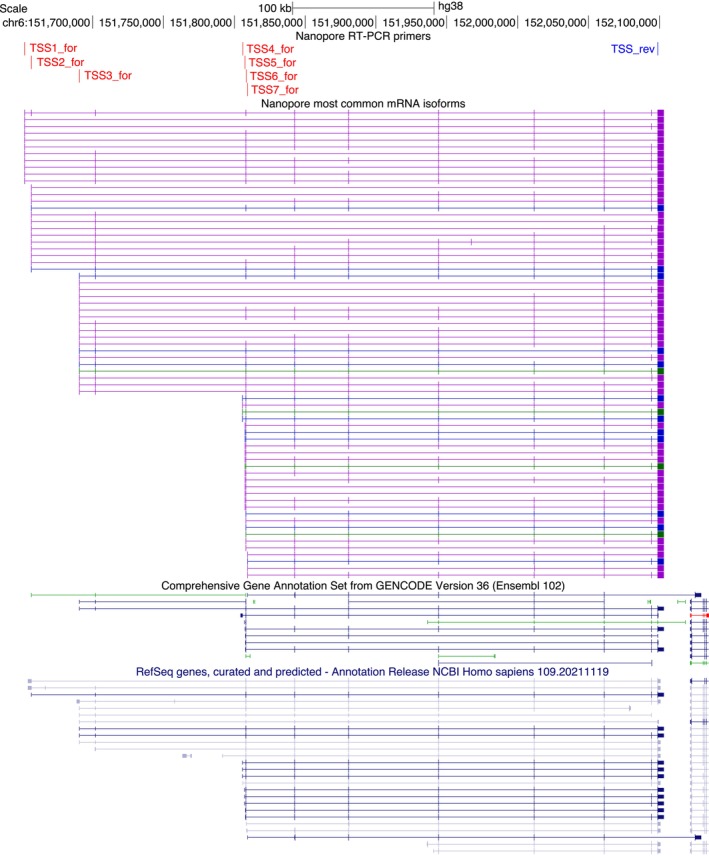
Schematic illustration of the transcript structure for the most common isoforms in the long‐read sequencing data. These are isoforms that together encompass 80% of the full‐length reads for each primer pair. Annotated transcripts from GENCODE V36 and RefSeq release 109 are shown for comparison.

Each of the seven different first exons was included in a large number of alternatively spliced isoforms, some with considerable variation in expression among the analysed samples (Figure S9). The most common isoforms (together encompassing 80% of the full‐length reads for each primer pair) are shown in Figure 2. The transcriptional complexity is generally higher in tumours and breast cancer cell lines than in healthy tissues, perhaps a result of general deregulation of the genome in cancer (Figure S10). The long‐read sequencing data confirm that there is a remarkable transcriptional complexity compared to the annotated isoforms and non‐negligible expression of some alternative isoforms.

### 3.4 Predicting protein‐coding sequences

We then searched the identified full‐length transcripts for open reading frames to compare with the coding sequence and known alternative protein isoforms (Figure S8). The open reading frames of the most common tier 1 protein isoforms for each targeted first exon (together encompassing 80% of the full‐length reads for a primer pair) are shown in Figure S11 together with a heatmap of their estimated relative expression. In addition to the full‐length ER, six of the isoforms had been previously annotated and eight were novel. The size distribution for tier 1 isoforms is shown in Figure S12 together with the predicted effects on the different protein domains of the ER.

### 3.5 Transcription factor activity of alternative isoforms

**FIGURE 3:**
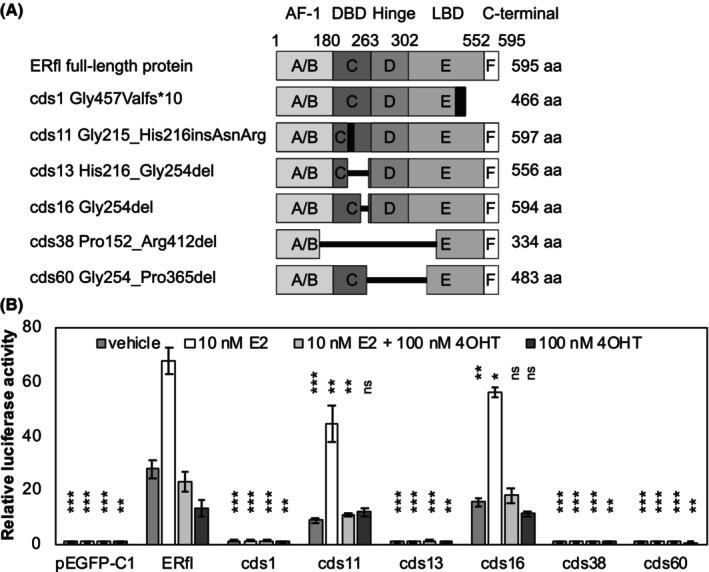
(A) Schematic illustration of the domain structure of the selected isoforms. (B) Isoform transcription factor activity on a dual‐luciferase reporter with firefly luciferase controlled by a 3xERE‐containing promoter. Luminescence from firefly luciferase was normalised to *Renilla* luciferase, and the activity was expressed relative to the baseline value for the empty pEGFP‐C1 vector with vehicle. ERfl = full‐length oestrogen receptor alpha (ER), vehicle control = 0.1% ethanol, E2 = oestradiol and 4OHT = 4‐hydroxytamoxifen. Bars indicate the mean of n = 3 replicates ± standard deviation. Two‐tailed Student's *t‐*test was used to test for significant differences in relative luciferase activity between each isoform and ERfl matched by treatment. *p < 0.05, **p < 0.01, ***p < 0.001, and ns = non‐significant.

Next, we cloned six alternative protein isoforms for functional evaluation. The isoforms were selected among the tier 1 transcripts that had the highest expression in the long‐read data, strong splice junction support in the breast tumour cohort and only contained one alteration compared to the full‐length ER. The isoforms are listed in Table 1, schematically illustrated in Figure 3A and validation of expression and expected sizes is shown in Figure S13. Among these isoforms, cds1 results from an exon‐skipping event that leads to a frame‐shift producing a truncated protein, cds11 uses an alternative splice donor leading to an insertion of two amino acids in the DBD (Figure S14), cds13 has a 39‐amino acid deletion in the DBD, cds16 has a single‐amino acid deletion in the DBD, while cds38 and cds60 have larger internal deletions affecting multiple domains. Two of these isoforms, cds13 and cds60, were not present in the databases (though cds13 has been previously described), while the others could be translated from GENCODE or RefSeq transcripts. Patient survival analysis for expression of these isoforms in the 3478 breast tumours is included in Figures S15–S17 and Tables S5 and S6.

We first assessed the ability of these isoforms to act as transcription factors in a luciferase reporter gene system with EREs. The hepatocellular carcinoma‐derived cell line HepG2 was selected since it is ER‐negative, gave the lowest ligand‐independent transcriptional background and had the clearest induction upon treatment with oestradiol. It is also a commonly used model system for ER function in the literature, which allows for comparison with other researchers' findings. Isoform expression plasmids were co‐transfected with pmirGLO‐3xERE, a dual‐luciferase vector engineered to contain firefly luciferase under control of a promoter containing three EREs. Cells were treated with vehicle control (0.1% ethanol), 10 nM E2, 10 nM E2 and 100 nM 4OHT (active metabolite of the ER antagonist tamoxifen) or 100 nM 4OHT. As shown in Figure 3B, two alternative isoforms possessed transcription factor activity, although with lower activity than the full‐length protein with vehicle (*p* = 8.6 × 10^−4^ and 5.7 × 10^−3^ for cds11 and cds16, respectively) and when stimulated with E2 (*p* = 8.3 × 10^−3^ and 1.8 × 10^−2^ for cds11 and cds16, respectively). Both alternative isoforms displayed decreased transcription activation similar to the full‐length ER upon treatment with 4OHT. Transcriptional induction was weaker for a reporter plasmid with a segment of the ERE‐containing *C3* promoter, but cds11 and cds16 were still the only active alternative isoforms (Figure S18).

Dose–response curves for E2 concentrations ranging from 1 pM to 100 nM for the full‐length protein and these alternative isoforms highlight the differences in baseline activity (without ligand) and maximum response (Figure S19). The estimated EC_50_ values were 165, 800 and 319 pM for full‐length ER, cds11 and cds16. This is in line with the previously reported EC_50_ value for binding of E2 by the full‐length ER of 160 ± 20 pM.^35^ In summary, the two isoforms with minimal alterations (cds11 and cds16) had moderately decreased transcription factor activity while isoforms with larger deletions involving the DBD (cds13, cds38 and cds60) were unable to induce transcription from a 3xERE‐driven luciferase reporter construct. The C‐terminally truncated isoform cds1 which lacks part of the LBD was also inactive.

### 3.6 Effects of co‐expression of alternative isoforms on ER function

In the previous experiments, we expressed one single isoform at a time in the ER‐negative cell line HepG2 but, normally, alternative isoforms are most likely co‐expressed with each other and with the full‐length ER protein. To study the possible effects of interaction between different isoforms, we therefore co‐transfected the plasmid encoding full‐length ER with each of the alternative isoforms and the 3xERE dual‐luciferase reporter plasmid in HepG2. As shown in Figure S20A, co‐transfection of the cds1, cds38 or cds60 isoforms (all inactive when transfected individually) led to increased luciferase activity in the vehicle control (*p* = 4.15 × 10^−3^, 3.02 × 10^−4^ and 2.24 × 10^−4^) and in the presence of 10 nM E2 (*p* = 2.95 × 10^−2^, 8.15 × 10^−4^ and 2.61 × 10^−2^). Co‐transfection of the cds13 isoform, which lacks part of the DBD, instead led to a marked decrease in luciferase activity in the vehicle control (*p =* 4.43 × 10^−2^) and to an even larger extent upon treatment with 10 nM E2 (*p =* 1.40 × 10^−5^).

For comparison, we also transfected the individual isoforms in the ER‐positive cell line MCF7 (Figure S20B). Overexpression of the full‐length ER significantly increased luciferase activity in the vehicle control and in cells treated with 10 nM E2 (*p* = 7.96 × 10^−4^ and 2.82 × 10^−2^, respectively). There was, however, no significant difference between MCF7 transfected with empty pEGFP‐C1 or ERfl after treatment with 10 nM E2 plus 100 nM 4OHT, or with 100 nM 4OHT. Only the decreased activity seen upon transfection of the cds13 isoform was reproducible and significant also in MCF7 cells, both in vehicle control and after treatment with 10 nM E2 (*p* = 4.01 × 10^−6^ and 1.92 × 10^−7^, respectively). Luciferase activity was also markedly lower upon treatment with 10 nM E2 plus 100 nM 4OHT or with 100 nM 4OHT (*p* = 7.36 × 10^−6^ and 1.43 × 10^−5^, respectively). These results suggest that the cds13 isoform, which lacks exon 3 and most of the DBD, acts as a dominant‐negative ER, as previously reported.^36^

### 3.7 Alternative isoforms differ in subcellular localisation

**FIGURE 4:**
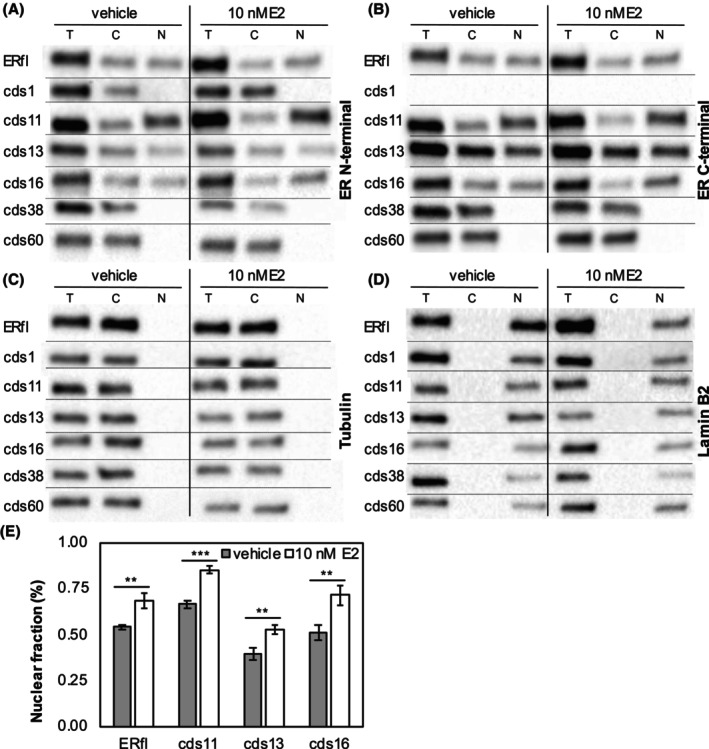
Subcellular fractionation followed by western blotting to determine the relative distribution between cytoplasm and nucleus for each isoform using (A) an N‐terminal or (B) a C‐terminal anti‐ESR1 antibody. (C) Tubulin is included as a control for the purity of the cytoplasmic fraction and (D) lamin B2 for the nuclear fraction. (E) Quantitation of nuclear fraction in vehicle control and after 30 min treatment with 10 nM E2. Bars indicate the mean of *n* = 3 replicates ± standard deviation. T = total lysate, C = cytoplasmic fraction and N = nuclear fraction, vehicle control = 0.1% ethanol, E2 = oestradiol, ERfl = full‐length oestrogen receptor alpha (ER). **p < 0.01 and ***p < 0.001.

To better understand the functional differences between ER isoforms, we analysed their subcellular localisation in HepG2 cells after a 30‐minute treatment with 10 nM E2. Cells were separated into cytoplasmic and nuclear fractions, and the amount of ER was analysed by western blotting. As shown in Figure 4, the full‐length ER and three of the alternative isoforms (cds11, cds13 and cds16) were present in both cytoplasm and nucleus, while the remaining isoforms (cds1, cds38 and cds60) were localised in the cytoplasm. A very small amount of protein, which did not increase upon E2 treatment, was observed for cds38 and cds60 in the nuclear fraction upon overexposure of the blot (data not shown), while tubulin remained negative. This may be a result of passive diffusion through the nuclear pore complex.^37^, ^38^ The isoforms that could enter the nucleus at appreciable levels also showed significantly increased nuclear localisation upon treatment with E2 (Figure 4E; Figures S21–S23). Interestingly, the cds11 isoform had significantly higher accumulation in the nucleus compared with the full‐length protein both with and without E2 (*p* = 3.4 × 10^−3^ and 7.3 × 10^−4^, respectively). The cds13 isoform, however, accumulated to a significantly lower degree in the nucleus both with and without E2 (*p* = 5.4 × 10^−3^ and 2.7 × 10^−3^, respectively). There was no significant difference between cds16 and full‐length ER, and in all cases, the fold change between cells treated with E2 or vehicle did not differ significantly between isoforms. Co‐transfection of full‐length ER and the isoforms that lacked transcription factor activity did not alter the distribution between nucleus and cytoplasm (Figures S24 and S25). In summary, only the three alternative isoforms that have an intact LBD were clearly capable of nuclear translocation and two of these differed from the full‐length ER in the subcellular distribution between nucleus and cytoplasm. The isoforms cds11 and cds16 were also the only tested isoforms that possessed transcription factor activity. Strikingly, the dominant‐negative isoform cds13 is also present in the nucleus, suggesting that it may act through heterodimerisation with full‐length ER. Analysis of subcellular localisation was also performed using immunofluorescence for HepG2 cells cultured on microscope chamber slides. The results support the cytoplasmic localisation of cds38 and cds60, but under these conditions cds1 was also localised to some extent in the nucleus (Figure S26).

### 3.8 Alternative isoforms differ in sensitivity to fulvestrant

**FIGURE 5:**
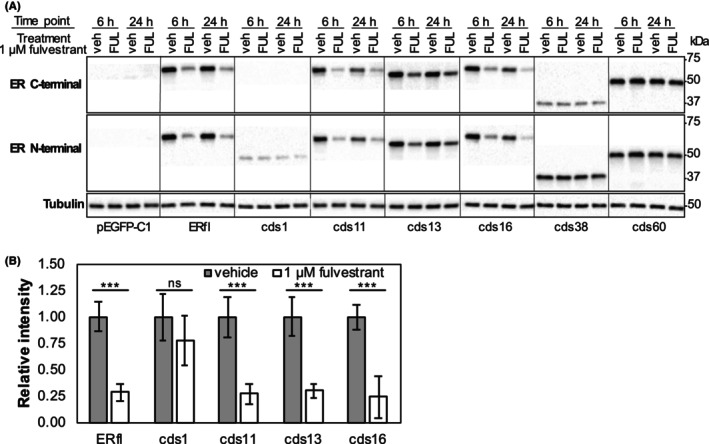
(A) HepG2 cells transfected with plasmids encoding full‐length ER or one of six alternative isoforms were treated for 6 and 24 h with vehicle control (0.01% DMSO) or 1 μM fulvestrant. ER expression was assessed by western blotting using N‐terminal and C‐terminal ER antibodies, and tubulin was used as a control for equal loading. (B) Quantitation of ER expression after 24 h treatment with vehicle or 1 μM fulvestrant by western blotting with an N‐terminal ER antibody. Band intensities were normalised to tubulin and expressed relative to the vehicle control for each isoform individually. Bars indicate the mean of *n* = 3 replicates ±standard deviation. **p < 0.01 and ns = not significant. pEGFP‐C1 = empty vector, ERfl = full‐length oestrogen receptor alpha (ER), kDa = kilo Dalton.

We then examined the sensitivity of the different isoforms to the SERD fulvestrant, a drug used in treatment of recurrent ER‐positive breast cancer. HepG2 cells transfected with plasmids encoding full‐length ER or one of the six alternative isoforms were treated with 1 μM fulvestrant or vehicle control (0.01% DMSO) for 6 and 24 h (Figure 5A; Figure S27). The isoforms that lack parts of the LBD (cds1, cds38 and cds60) were insensitive to treatment with fulvestrant. The remaining alternative isoforms were degraded to the same extent as the full‐length isoform (Figure 5B; Figures S28 and S29).

### 3.9 Validation of translation of novel isoforms

To verify that the endogenous mRNA isoforms selected for functional studies are translated into the corresponding protein isoforms, we performed polysome fractionation and isoform‐specific real‐time RT‐PCR for the three breast cancer cell lines that were used for long‐read sequencing (BT‐474, MCF7 and T47D, Figures S30 and S31). Five out of six isoforms were readily detectable in both total RNA and the polysome fractions that contain highly translated mRNAs, indicating that these ER transcripts encode peptides. The sixth isoform, cds38, was barely detectable and only in BT‐474 and MCF7, but also had the lowest expression among the analysed isoforms in the long‐read sequencing data. These results show that the alternative mRNA isoforms that were selected for functional evaluation are endogenously expressed and translated in human cells.

### 3.10 Regulation of promoter usage and alternative splicing

Our results show that the *ESR1* locus uses multiple alternative first, internal and last exons, as well as alternative splice donor and acceptor sites to create a large number of different transcripts. To study the genomic regulation of these complex events, we first analysed transcription factor chromatin immunoprecipitation sequencing (ChIP‐Seq) data from the UniBind database.^29^ Compared to cells of breast origin, cells derived from liver and prostate have more pronounced binding in the distal, upstream promoter regions (TSS1‐3) than in the proximal promoter region (TSS4‐8, Figures S32A and S32B). Transcripts from the proximal promoter region also have the highest overall expression in breast tumours. Next, we compared methylation at CpG sites with ER expression in breast tumours from the The Cancer Genome Atlas (TCGA).^30^ Significant negative correlations at some sites suggest that methylation of the CpG island shores may have a strong effect on transcription from the proximal promoter in breast cancer (Figure S32C).

To study the many splice sites that contribute to the generation of a large number of different transcript isoforms, we analysed evolutionary conservation using phyloP^31^ scores and predicted splice site strength using MaxEntScan.^33^ Canonical splice sites tend to be more conserved than alternative splice sites and most, but not all, splice sites in novel exons are non‐conserved (Figure S33A,B). As expected, canonical splice sites also have stronger splice recognition sequences than alternative splice sites according to the MaxEntScan scores that measure similarity to a human splice site consensus sequence (Figure S33C and D). Interestingly, most donors and acceptors in novel exons have strong (though non‐conserved) splice sites. As shown in Figure S34, phyloP scores are a better predictor of splice site expression than MaxEntScan scores and conserved splice sites tend to have higher expression than non‐conserved sites. These results suggest that alternative splice sites in well‐expressed exons are used more rarely than their canonical counterparts because they are inherently weaker and therefore presumably depend on other regulatory elements and splicing factors for activation. The novel exons mostly have non‐conserved splice sites that are lowly expressed although they possess strong splice sites. Perhaps, these evolutionarily young exons have not yet acquired the necessary enhancer elements but represent a reservoir for future evolution of alternative ER isoforms.

## 4 DISCUSSION

The ER is the single most important biomarker in breast cancer; it provides prognostic information, guides the choice of treatment and is a target for therapy. As a nuclear steroid hormone receptor, it is also a commonly used model for transcriptional regulation. Here, we have created a comprehensive map of alternative transcripts and splicing events in the ER through in‐depth analysis of short‐ and long‐read RNA‐Seq data from breast tumours, cell lines and normal tissues. Some of the novel events are expressed in the range of previously annotated exons and splice junctions and for some there is considerable variation between breast tumours. We have furthermore identified numerous possible alternative protein isoforms and performed functional studies for six isoforms. Two alternative isoforms retain transcription factor activity (cds11 and cds16) and show nuclear accumulation that increases upon oestrogen stimulation. A similar change in subcellular localisation was observed for a transcriptionally inactive isoform (cds13) lacking a large part of the DBD that is encoded in exon 3. This isoform acts as a dominant‐negative receptor in co‐expression experiments, in agreement with the literature.^36^ Three isoforms with a partial LBD (cds1, cds38 and cds60) were all resistant to degradation upon treatment with fulvestrant but lacked transcription factor activity.

A nuclear localisation signal (NLS) with three stretches of basic amino acids has previously been identified between the DBD and Hinge domains (amino acids 243‐272).^37^ In our experiments, isoforms with changes up to and including glycine 254 (which retain two of three motifs) were still capable of nuclear translocation. The two isoforms with larger deletions of this region were cytoplasmic. These results are in line with the conclusions of Moriyama et al^37^ The results for cds1 are somewhat unclear; ER lacking exon 7 has been reported to be capable of nuclear translocation and inhibit transcriptional activity of the full‐length receptor.^34^, ^39^ We were not able to detect this isoform in nuclear extract after subcellular fractionation, also not upon co‐expression with the full‐length isoform, and did not detect a dominant‐negative effect on transactivation of an ERE‐driven luciferase reporter. However, in the immunofluorescence analysis where cells were cultured on microscope slides some nuclear cds1 was seen, and the fraction increased upon treatment with E2.

Some of the most frequently cited alternative ER isoforms are the 46, 36 and 30 kDa proteins. The 46 kDa isoform has been described as a negative regulator of the full‐length ER.^40^ It uses a start codon in exon 2 of the canonical transcript and lacks the activation function 1 (AF‐1) domain. This isoform can be translated from transcripts originating from the first exon that is located in the intron downstream of the main first exon (TSS7 in Figure 4). The coding sequence corresponds to cds6 in Figure 5. It is one of the most common coding sequence isoforms in the long‐read sequencing data, especially in some normal tissues and cell lines. The expression in tumours is relatively low, however, according to both short‐ and long‐read RNA‐Seq data. An internal ribosome entry site (IRES) has been reported that could potentially allow production of this protein isoform from other transcripts.^41^ The 36 kDa isoform is also translated from transcripts starting at the intronic promoter downstream of the main first exon, but skips exons 7–8 of the canonical transcript and splices to a downstream last exon.^21^ It produces a protein that lacks the AF‐1 and a large part of the AF‐2 domain. It was reported to be localised at the cell membrane and to be involved in non‐genomic ER signalling activity. In our first experiments on cell lines, RT‐PCR with a reverse primer in the downstream alternative last exon did not produce any detectable products and it was therefore excluded from the long‐read sequencing. In the breast tumour short‐read data, on average only 1% of reads spliced from exon 6 correspond to this splicing event, and it is rare compared to other alternative splicing events in the region including exon 7‐skipping and splicing to an intronic cryptic exon. The 30 kDa isoform was reported to result from splicing between novel internal splice sites in exons 4 and 6 and exclusion of exon 7.^22^ We could not detect any such isoform in our short‐ or long‐read RNA‐Seq data, not even in the unfiltered splice junction set. These results suggest that the most frequently cited alternative isoforms do not make up an exhaustive list and that some comparatively more common alternative isoforms such as cds11, cds13 and cds16 deserve further study.

We initiated this project because it was clear from breast tumour RNA‐Seq data that there were both exons and alternative splicing events in the *ESR1* locus that were not present in GENCODE or RefSeq, the main transcript annotation databases. Another issue was the many and striking discrepancies between the two databases. For example, the isoform with exon 7‐skipping that is referred to as cds1 in this paper has been previously reported as ER∆7^34^, ^42^ and is present in RefSeq but not in GENCODE. Well into the project we realised that our cds13, which is absent from both GENCODE and RefSeq, has been reported as ER∂E3.^36^ *ESR1* is a well‐known gene that has been studied for decades but the lack of inclusion of existing data on transcript isoforms and inconsistencies between databases severely hamper the possibilities to do good science. This problem has not disappeared with easy access to high‐throughput sequencing methods, which have made researchers even more dependent on the quality and completeness of available genomic annotation.

From our experiments, it is apparent that the ability and sensitivity to detect different ER isoforms using N‐ or C‐terminal anti‐ER antibodies vary considerably depending on which protein domains that are included. In the clinical management of breast cancer, IHC is used in routine pathology to determine the expression of the ER and the result guides the choice of treatment.^18^ When there is a disagreement in ER status between IHC and RNA‐Seq in a patient where both data types are available, it could indicate that more careful investigation is needed—potentially, there might be tumours classified as ER‐negative in patients that could benefit from endocrine therapy. It has already been observed that a number of antibodies used for breast cancer diagnosis differ markedly in their ability to detect ER isoforms.^43^ Looking at the binding sites for three antibodies that are commonly used in clinical IHC analysis (https://www.nordiqc.org/downloads/assessments/159_2.pdf),^44^, ^45^ two N‐terminal antibodies (EP1 and 6F11) could theoretically detect all six alternative isoforms that were used in our functional studies, while the C‐terminal SP1 would miss cds1. For the tier 1 protein isoforms that were predicted from our long‐read sequencing data, EP1 and 6F11 should be able to detect 60% and SP1 49% of isoforms. Clinical differences have already been reported for some isoforms but these have been studied in isolation. If a careful evaluation of all identified alternative ER isoforms concludes that some of these provide relevant prognostic and treatment‐predictive information, isoform‐level expression could be introduced in the clinic to help identify the best treatment strategy for the individual patient.

The transcriptional map of *ESR1* that is presented here has novel first, internal and last exons, as well as splice junctions and splice site combinations that together result in a large number of expressed mRNAs and potential protein isoforms. Many of these have not been described before and the functional characterisation of six alternative isoforms together with full‐length ER is, to the best of our knowledge, the most extensive comparison made so far. Our work is, however, mostly focused on expression in breast tumours, although samples representing 12 different normal tissues (including breast, cervix and ovary) were included in the long‐read sequencing. Future work should explore the functional roles of alternative ER isoforms in normal breast and reproductive organs, as well as in tumour biology.

In the functional studies performed here, we mainly used the hepatocellular carcinoma‐derived cell line HepG2. It is a convenient cell line to use since it is ER‐negative and gives a robust response for ERE‐driven luciferase reporters upon co‐transfection with an ER expression vector. It is also an established model for functional studies of the ER, which allowed us to compare our results with the existing literature. However, it would not be a suitable model system to use for more detailed studies of transcriptional targets or protein interactions where critical DNA modifications and protein partners would differ in a naturally ER‐positive cellular environment. A possible alternative could be knockout of endogenous ER with concomitant replacement with different alternative isoforms. We have attempted to do isoform‐specific knockdowns using siRNAs, but for isoforms that do not contain any unique exonic sequence it has not been possible to achieve specific knockdown. As expected, siRNAs placed across splice junctions still have sufficient complementarity to affect expression of full‐length ER through a miRNA‐like mechanism. It would also be interesting to develop methods to specifically detect alternative isoforms at the protein level. We have tested mass spectrometry for detection of the six alternative isoforms that were used for functional studies in this manuscript, but each isoform only generates one unique peptide and the sensitivity in our initial tests was insufficient to detect these even in transfected HepG2.

In summary, our results demonstrate an unanticipated complexity in the expression of alternative mRNA isoforms of the ER and provide extensive functional characterisation for six alternative protein isoforms. Two of these isoforms are active transcription factors, one acts as a dominant‐negative receptor when co‐expressed with full‐length ER, and three are transcriptionally inactive but are resistant to degradation by fulvestrant. These results will aid in the interpretation of clinical and experimental data and inform functional studies to improve our understanding of the ER in health and disease.

## AUTHOR CONTRIBUTIONS

**Carlos Enrique Balcazar Lopez:** Formal analysis (supporting); funding acquisition (supporting); investigation (supporting); methodology (supporting); validation (supporting); visualization (supporting); writing – original draft (supporting); writing – review and editing (supporting). **Juliane Albrecht:** Formal analysis (supporting); investigation (supporting); software (supporting); validation (supporting); visualization (supporting); writing – original draft (supporting); writing – review and editing (supporting). **Völundur Hafstad:** Formal analysis (supporting); software (supporting); visualization (supporting); writing – original draft (supporting); writing – review and editing (supporting). **Cornelia Börjesson Freitag:** Investigation (supporting); validation (supporting); writing – review and editing (supporting). **Johan Vallon‐Christersson:** Data curation (supporting); resources (supporting); writing – review and editing (supporting). **Cristian Bellodi:** Resources (supporting); writing – review and editing (supporting). **Helena Persson:** Conceptualization (lead); data curation (supporting); formal analysis (lead); funding acquisition (lead); investigation (lead); methodology (lead); project administration (lead); resources (supporting); software (lead); supervision (lead); validation (lead); visualization (lead); writing – original draft (lead); writing – review and editing (lead).

## CONFLICT OF INTEREST STATEMENT

The authors do not declare any conflicts of interest.

## ETHICS STATEMENT

For the SCAN‐B data, the study was conducted in accordance with the Declaration of Helsinki and has been approved by the Regional Ethical Review Board of Lund (2007/155, 2009/658, 2009/659, 2014/8), the county governmental biobank centre and the Swedish Data Inspection group (364‐2010). Written information was given by trained health professionals, and all patients provided written informed consent.

## Supporting information


Data S1.

Data S2.

Table S4.

## Data Availability

The results shown here are in part based upon data generated by the TCGA Research Network: https://www.cancer.gov/tcga. Due to Swedish law, the patient consent, and the risk that the sequence data contain person‐identifiable information and hereditary mutations, we cannot deposit the SCAN‐B raw sequence data in a repository. The short‐read dataset supporting the conclusions of this article is available in the NCBI Gene Expression Omnibus (GEO) repository, accession number GSE96058, and from the corresponding author upon request. The long‐read sequencing data for commercially available cell lines and healthy tissues have been deposited in the NCBI Gene Expression Omnibus (GEO) repository, accession number GSE208238.
