# Correction to ‘Alternative promoters and splicing create multiple functionally distinct isoforms of oestrogen receptor alpha in breast cancer and healthy tissues’

**DOI:** 10.1002/cam4.71829

**Published:** 2026-04-11

Balcazar Lopez CE, Albrecht J, Hafstað V, Börjesson Freitag C, Vallon‐Christersson J, Bellodi C, Persson H. Alternative promoters and splicing create multiple functionally distinct isoforms of oestrogen receptor alpha in breast cancer and healthy tissues. Cancer Med. 2023 Sep;12(18):18931‐18945. doi:10.1002/cam4.6508.

The following text was omitted from the Acknowledgements: This project has received funding from the European Union's Horizon 2020 research and innovation programme under the Marie Skłodowska‐Curie grant agreement No. 847583—CanFaster Postdoc.

The correct Acknowledgements should be:

The authors would like to acknowledge patients, clinicians and hospital staff participating in the SCAN‐B study, the staff at the central SCAN‐B laboratory at the Division of Oncology, Lund University, the Swedish National Breast Cancer Quality Registry (NKBC), Regional Cancer Centre South and the South Swedish Breast Cancer Group (SSBCG). This project has received funding from the European Union's Horizon 2020 research and innovation programme under the Marie Skłodowska‐Curie grant agreement No. 847583—CanFaster Postdoc.

We apologize for this error.

