# Supplementary material for: Alternative promoters and splicing create multiple functionally distinct isoforms of oestrogen receptor alpha in breast cancer and healthy tissues
Source: Cancer Med. 2023 Sep 7;12(18):18931–45. doi: 10.1002/cam4.6508 (PMC10557849; doi:10.1002/cam4.6508)
Supplement: Supplementary file 1 — Data S1. [file CAM4-12-18931-s003.pdf]

Alternative promoters and splicing create multiple functionally distinct isoforms of estrogen receptor alpha in breast cancer and healthy tissues

Carlos Enrique Balcazar Lopez, Juliane Albrecht, Völundur Hafstað, Cornelia Börjesson Freitag, Johan Vallon-Christersson, Cristian Bellodi & Helena Persson

Table of Contents

Supplementary Table S1……………………………………………………………2
Supplementary Table S2……………………………………………………………3
Supplementary Table S3……………………………………………………………4
Supplementary Figure S1…………………………………………………………..5
Supplementary Figure S2…………………………………………………………..6
Supplementary Figure S3…………………………………………………………..7
Supplementary Figure S4…………………………………………………………..8
Supplementary Figure S5…………………………………………………………..9
Supplementary Figure S6…………………………………………………………..10
Supplementary Figure S7…………………………………………………………..11
Supplementary Figure S8…………………………………………………………..12
Supplementary Figure S9…………………………………………………………..13
Supplementary Figure S10…………………………………………………………14
Supplementary Figure S11…………………………………………………………15
Supplementary Figure S12…………………………………………………………16
Supplementary Figure S13…………………………………………………………17
Supplementary Figure S14…………………………………………………………18
Supplementary Figure S15…………………………………………………………19
Supplementary Figure S16…………………………………………………………20
Supplementary Figure S17…………………………………………………………21
Supplementary Table S5…………………………………………………………...22
Supplementary Table S6…………………………………………………………...23
Supplementary Figure S18…………………………………………………………24
Supplementary Figure S19…………………………………………………………25
Supplementary Figure S20…………………………………………………………26
Supplementary Figure S21…………………………………………………………27
Supplementary Figure S22…………………………………………………………28
Supplementary Figure S23…………………………………………………………29
Supplementary Figure S24…………………………………………………………30
Supplementary Figure S25…………………………………………………………31
Supplementary Figure S26…………………………………………………………32
Supplementary Figure S27…………………………………………………………33
Supplementary Figure S28…………………………………………………………34
Supplementary Figure S28…………………………………………………………35
Supplementary Figure S30…………………………………………………………36
Supplementary Figure S31…………………………………………………………37
Supplementary Figure S32…………………………………………………………38
Supplementary Figure S33…………………………………………………………39
Supplementary Figure S34…………………………………………………………40

Supplementary Table S4 is available separately as an .xlsx file.

Supplementary Table S1. List of primers used and their applications.

Primer	Sequence	Comment
TC_15_Forward	GTACTCCAAATAATCCACAAGAATTAATTG	RT-PCR TSS1
TC_15_Reverse	GAGTGTATGCAGGAGACAGAATTTG	RT-PCR TSS1
TC_16_Forward	CTCAAATATCTCCAAAATCTGATACC	RT-PCR TSS2
TC_16_Reverse	GTGGTGCATGATGAGGGTAAA	RT-PCR TSS2
TC_54_Forward	TCCTTTCTCCTGCCCATTCTATAG	RT-PCR TSS3
TC_54_Reverse	ACCACGTTCTTGCACTTCAT	RT-PCR TSS3
TC_74_Forward	CTCGGCCCTTGACTTCTACA	RT-PCR TSS4
TC_74_Reverse	ACCACGTTCTTGCACTTCAT	RT-PCR TSS4
2740_Forward	GCACATAAGGCAGCACATTAGA	RT-PCR TSS5
2740_Reverse	CTTCATGCTGTACAGATGCTCC	RT-PCR TSS5
CDS_Forward	CTACTACCTGGAGAACGAGCCC	RT-PCR TSS6
CDS_Reverse	AAGGAATGCGATGAAGTAGAGC	RT-PCR TSS6
8100_Forward	GGACACAGGAGACCACTTTG	RT-PCR TSS7
8100_Reverse	ACCACGTTCTTGCACTTCAT	RT-PCR TSS7
ER_5prime_for	AAAAGCTAGCCGGTCTGCACC	fragment amplification
ER_5prime_rev	TTTTGGCCGGCCTCGCGCA	fragment amplification
ER_3prime_for	AAAAGGCCGGCCCGCCGGCA	fragment amplification
ERfl_3prime_rev	TTTTGGATCCTCAGACCGTGG	fragment amplification
ERisoforms_3prime_rev	TTTTGGATCCTCATGCTGTACAGAT	fragment amplification
cds11_SDM_for	CTTCAAGAGAAGTATTCAAGGTAATAGACATAACGA CTATATGTGTCC	site-directed mutagenesis
cds11_SDM_rev	GGACACATATAGTCGTTATGTCTATTACCTTGAATAC TTCTCTTGAAG	site-directed mutagenesis
cds16_SDM_for	AGTGGGAATGATGAAAGGGATACGAAAAGACCGAA G	site-directed mutagenesis
cds16_SDM_rev	CTTCGGTCTTTTCGTATCCCTTTCATCATTCCCACT	site-directed mutagenesis
pEGFP-C1_for	TGAACCGTCAGATCCGCTAG	Sanger sequencing
pEGFP-C1_rev	TGAGTTTGGACAAACCACCACAAC	Sanger sequencing
ESR1_internal_for	CAGGGTGGCAGAGAAAGATTGG	Sanger sequencing
ESR1_internal_rev	GTGCCTGATGTGGGAGAGGATGA	Sanger sequencing
3xERE_NdeI_for	ATGCCATATGAGGTACCGAGCTCTTACGC	amplification 3xERE
3xERE_SmaI_rev	ATGCCCCGGGTGCAGGTCGACTCTAGAGGA	amplification 3xERE
C3-LUC_NdeI_for	TAGTCATATGCTGAGCTAACATAACCCGGG	amplification C3 promoter
C3-LUC_EcoRI_rev	ATCCGAATTCCAACAGTACCGGAATGCCAA	amplification C3 promoter
pmirGLO_for	TTACTGTCTGCCCTCGTAGC	Sanger sequencing
pmirGLO_rev	TTCGAGTGGGTAGAATGGCG	Sanger sequencing
cds1_qPCR_for	TGTAGAGGGCATGGTGGAGA	real-time RT-PCR
cds1_qPCR_rev	CTCCATGCCTTTGTTACAGAATT	real-time RT-PCR
cds11-13_qPCR_for	GGAGACTCGCTACTGTGCAG	real-time RT-PCR
cds11_qPCR_rev	AGTCGTTATGTCTATTACCTTGAA	real-time RT-PCR
cds13_qPCR_rev	TCGGTCTTTTCGTATCCCTTGA	real-time RT-PCR
cds16-60_qPCR_for	CACCAACCAGTGCACCATTG	real-time RT-PCR
cds16_qPCR_rev	CTTCGGTCTTTTCGTATCCCTTT	real-time RT-PCR
cds60_qPCR_rev	GGTCAAATCCACAAAGCCACC	real-time RT-PCR
cds38_qPCR_for	CTCAACAGCGTGTCTCCGAG	real-time RT-PCR
cds38_qPCR_rev	ACATTTTCCCTGGTTCCTGTAGA	real-time RT-PCR
ER_3UTR_for	ACACAGACCCCTTTGCATTC	real-time RT-PCR
ER_3UTR_rev	TGTAGTGCACAAAAAGCATTG	real-time RT-PCR
MRPL19_for	TCGAAGGACAAGGTGTCGAG	real-time RT-PCR
MRPL19_rev	ATTCAGGAAGGGCATCTCGT	real-time RT-PCR

Supplementary Table S2. Fragment design for ER isoform cloning in pEGFP-C1 and for creation of a multiple cloning site in pmirGLO.

ER_5prime
AAAAGCTAGCCGGTCTGCACCCTGCCCGCGGCCACGGACCATGACCATGACCCTCCACACCAAAGCATCTGGGATGGCCCTACTGCATCAGATC
CAAGGGAACGAGCTGGAGCCCCTGAACCGTCCGCAGCTCAAGATCCCCCTGGAGCGGCCCCTGGGCGAGGTGTACCTGGACAGCAGCAAGCCC
GCCGTGTACAACTACCCCGAGGGCGCCGCCTACGAGTTCAACGCCGCGGCCGCCGCCAACGCGCAGGTCTACGGTCAGACCGGCCTCCCCTACG
GCCCCGGGTCTGAGGCTGCGGCGTTCGGCTCCAACGGCCTGGGGGGTTTCCCCCCACTCAACAGCGTGTCTCCGAGCCCGCTGATGCTACTGCAC
CCGCCGCCGCAGCTGTCGCCTTTCCTGCAGCCCCACGGCCAGCAGGTGCCCTACTACCTGGAGAACGAGCCCAGCGGCTACACGGTGCGCGAGG
CCGGCCAAAA

ERfl_3prime
AAAAGGCCGGCCCGCCGGCATTCTACAGGCCAAATTCAGATAATCGACGCCAGGGTGGCAGAGAAAGATTGGCCAGTACCAATGACAAGGGAA
GTATGGCTATGGAATCTGCCAAGGAGACTCGCTACTGTGCAGTGTGCAATGACTATGCTTCAGGCTACCATTATGGAGTCTGGTCCTGTGAGGGC
TGCAAGGCCTTCTTCAAGAGAAGTATTCAAGGACATAACGACTATATGTGTCCAGCCACCAACCAGTGCACCATTGATAAAAACAGGAGGAAGA
GCTGCCAGGCCTGCCGGCTCCGTAAATGCTACGAAGTGGGAATGATGAAAGGTGGGATACGAAAAGACCGAAGAGGAGGGAGAATGTTGAAAC
ACAAGCGCCAGAGAGATGATGGGGAGGGCAGGGGTGAAGTGGGGTCTGCTGGAGACATGAGAGCTGCCAACCTTTGGCCAAGCCCGCTCATGA
TCAAACGCTCTAAGAAGAACAGCCTGGCCTTGTCCCTGACGGCCGACCAGATGGTCAGTGCCTTGTTGGATGCTGAGCCCCCGATACTCTATTCC
GAGTATGATCCTACCAGACCCTTCAGTGAAGCTTCGATGATGGGCTTACTGACCAACCTGGCAGACAGGGAGCTGGTTCACATGATCAACTGGG
CGAAGAGGGTGCCAGGCTTTGTGGATTTGACCCTCCATGATCAGGTCCACCTTCTAGAATGTGCCTGGCTAGAGATCCTGATGATTGGTCTCGTC
TGGCGCTCCATGGAGCACCCAGGGAAGCTACTGTTTGCTCCTAACTTGCTCTTGGACAGGAACCAGGGAAAATGTGTAGAGGGCATGGTGGAGA
TCTTCGACATGCTGCTGGCTACATCATCTCGGTTCCGCATGATGAATCTGCAGGGAGAGGAGTTTGTGTGCCTCAAATCTATTATTTTGCTTAATT
CTGGAGTGTACACATTTCTGTCCAGCACCCTGAAGTCTCTGGAAGAGAAGGACCATATCCACCGAGTCCTGGACAAGATCACAGACACTTTGAT
CCACCTGATGGCCAAGGCAGGCCTGACCCTGCAGCAGCAGCACCAGCGGCTGGCCCAGCTCCTCCTCATCCTCTCCCACATCAGGCACATGAGT
AACAAAGGCATGGAGCATCTGTACAGCATGAAGTGCAAGAACGTGGTGCCCCTCTATGACCTGCTGCTGGAGATGCTGGACGCCCACCGCCTAC
ATGCGCCCACTAGCCGTGGAGGGGCATCCGTGGAGGAGACGGACCAAAGCCACTTGGCCACTGCGGGCTCTACTTCATCGCATTCCTTGCAAAA
GTATTACATCACGGGGGAGGCAGAGGGTTTCCCTGCCACGGTCTGAGGATCCAAAA

cds1_3prime
AAAAGGCCGGCCCGCCGGCATTCTACAGGCCAAATTCAGATAATCGACGCCAGGGTGGCAGAGAAAGATTGGCCAGTACCAATGACAAGGGAA
GTATGGCTATGGAATCTGCCAAGGAGACTCGCTACTGTGCAGTGTGCAATGACTATGCTTCAGGCTACCATTATGGAGTCTGGTCCTGTGAGGGC
TGCAAGGCCTTCTTCAAGAGAAGTATTCAAGGACATAACGACTATATGTGTCCAGCCACCAACCAGTGCACCATTGATAAAAACAGGAGGAAGA
GCTGCCAGGCCTGCCGGCTCCGTAAATGCTACGAAGTGGGAATGATGAAAGGTGGGATACGAAAAGACCGAAGAGGAGGGAGAATGTTGAAAC
ACAAGCGCCAGAGAGATGATGGGGAGGGCAGGGGTGAAGTGGGGTCTGCTGGAGACATGAGAGCTGCCAACCTTTGGCCAAGCCCGCTCATGA
TCAAACGCTCTAAGAAGAACAGCCTGGCCTTGTCCCTGACGGCCGACCAGATGGTCAGTGCCTTGTTGGATGCTGAGCCCCCGATACTCTATTCC
GAGTATGATCCTACCAGACCCTTCAGTGAAGCTTCGATGATGGGCTTACTGACCAACCTGGCAGACAGGGAGCTGGTTCACATGATCAACTGGG
CGAAGAGGGTGCCAGGCTTTGTGGATTTGACCCTCCATGATCAGGTCCACCTTCTAGAATGTGCCTGGCTAGAGATCCTGATGATTGGTCTCGTC
TGGCGCTCCATGGAGCACCCAGGGAAGCTACTGTTTGCTCCTAACTTGCTCTTGGACAGGAACCAGGGAAAATGTGTAGAGGGCATGGTGGAGA
TCTTCGACATGCTGCTGGCTACATCATCTCGGTTCCGCATGATGAATCTGCAGGGAGAGGAGTTTGTGTGCCTCAAATCTATTATTTTGCTTAATT
CTGTAACAAAGGCATGGAGCATCTGTACAGCATGAGGATCCAAAA

cds13_3prime
AAAAGGCCGGCCCGCCGGCATTCTACAGGCCAAATTCAGATAATCGACGCCAGGGTGGCAGAGAAAGATTGGCCAGTACCAATGACAAGGGAA
GTATGGCTATGGAATCTGCCAAGGAGACTCGCTACTGTGCAGTGTGCAATGACTATGCTTCAGGCTACCATTATGGAGTCTGGTCCTGTGAGGGC
TGCAAGGCCTTCTTCAAGAGAAGTATTCAAGGGATACGAAAAGACCGAAGAGGAGGGAGAATGTTGAAACACAAGCGCCAGAGAGATGATGG
GGAGGGCAGGGGTGAAGTGGGGTCTGCTGGAGACATGAGAGCTGCCAACCTTTGGCCAAGCCCGCTCATGATCAAACGCTCTAAGAAGAACAG
CCTGGCCTTGTCCCTGACGGCCGACCAGATGGTCAGTGCCTTGTTGGATGCTGAGCCCCCGATACTCTATTCCGAGTATGATCCTACCAGACCCT
TCAGTGAAGCTTCGATGATGGGCTTACTGACCAACCTGGCAGACAGGGAGCTGGTTCACATGATCAACTGGGCGAAGAGGGTGCCAGGCTTTGT
GGATTTGACCCTCCATGATCAGGTCCACCTTCTAGAATGTGCCTGGCTAGAGATCCTGATGATTGGTCTCGTCTGGCGCTCCATGGAGCACCCAG
GGAAGCTACTGTTTGCTCCTAACTTGCTCTTGGACAGGAACCAGGGAAAATGTGTAGAGGGCATGGTGGAGATCTTCGACATGCTGCTGGCTAC
ATCATCTCGGTTCCGCATGATGAATCTGCAGGGAGAGGAGTTTGTGTGCCTCAAATCTATTATTTTGCTTAATTCTGGAGTGTACACATTTCTGTC
CAGCACCCTGAAGTCTCTGGAAGAGAAGGACCATATCCACCGAGTCCTGGACAAGATCACAGACACTTTGATCCACCTGATGGCCAAGGCAGGC
CTGACCCTGCAGCAGCAGCACCAGCGGCTGGCCCAGCTCCTCCTCATCCTCTCCCACATCAGGCACATGAGTAACAAAGGCATGGAGCATCTGT
ACAGCATGAAGTGCAAGAACGTGGTGCCCCTCTATGACCTGCTGCTGGAGATGCTGGACGCCCACCGCCTACATGCGCCCACTAGCCGTGGAGG
GGCATCCGTGGAGGAGACGGACCAAAGCCACTTGGCCACTGCGGGCTCTACTTCATCGCATTCCTTGCAAAAGTATTACATCACGGGGGAGGCA
GAGGGTTTCCCTGCCACGGTCTGAGGATCCAAAA

cds38_3prime
AAAAGGCCGGCCCGCCGGCATTCTACAGGAACCAGGGAAAATGTGTAGAGGGCATGGTGGAGATCTTCGACATGCTGCTGGCTACATCATCTCG
GTTCCGCATGATGAATCTGCAGGGAGAGGAGTTTGTGTGCCTCAAATCTATTATTTTGCTTAATTCTGGAGTGTACACATTTCTGTCCAGCACCCT
GAAGTCTCTGGAAGAGAAGGACCATATCCACCGAGTCCTGGACAAGATCACAGACACTTTGATCCACCTGATGGCCAAGGCAGGCCTGACCCTG
CAGCAGCAGCACCAGCGGCTGGCCCAGCTCCTCCTCATCCTCTCCCACATCAGGCACATGAGTAACAAAGGCATGGAGCATCTGTACAGCATGA
AGTGCAAGAACGTGGTGCCCCTCTATGACCTGCTGCTGGAGATGCTGGACGCCCACCGCCTACATGCGCCCACTAGCCGTGGAGGGGCATCCGT
GGAGGAGACGGACCAAAGCCACTTGGCCACTGCGGGCTCTACTTCATCGCATTCCTTGCAAAAGTATTACATCACGGGGGAGGCAGAGGGTTTC
CCTGCCACGGTCTGAGGATCCAAAA

cds60_3prime
AAAAGGCCGGCCCGCCGGCATTCTACAGGCCAAATTCAGATAATCGACGCCAGGGTGGCAGAGAAAGATTGGCCAGTACCAATGACAAGGGAA
GTATGGCTATGGAATCTGCCAAGGAGACTCGCTACTGTGCAGTGTGCAATGACTATGCTTCAGGCTACCATTATGGAGTCTGGTCCTGTGAGGGC
TGCAAGGCCTTCTTCAAGAGAAGTATTCAAGGACATAACGACTATATGTGTCCAGCCACCAACCAGTGCACCATTGATAAAAACAGGAGGAAGA
GCTGCCAGGCCTGCCGGCTCCGTAAATGCTACGAAGTGGGAATGATGAAAGGTGGCTTTGTGGATTTGACCCTCCATGATCAGGTCCACCTTCTA
GAATGTGCCTGGCTAGAGATCCTGATGATTGGTCTCGTCTGGCGCTCCATGGAGCACCCAGGGAAGCTACTGTTTGCTCCTAACTTGCTCTTGGA
CAGGAACCAGGGAAAATGTGTAGAGGGCATGGTGGAGATCTTCGACATGCTGCTGGCTACATCATCTCGGTTCCGCATGATGAATCTGCAGGGA
GAGGAGTTTGTGTGCCTCAAATCTATTATTTTGCTTAATTCTGGAGTGTACACATTTCTGTCCAGCACCCTGAAGTCTCTGGAAGAGAAGGACCA
TATCCACCGAGTCCTGGACAAGATCACAGACACTTTGATCCACCTGATGGCCAAGGCAGGCCTGACCCTGCAGCAGCAGCACCAGCGGCTGGCC
CAGCTCCTCCTCATCCTCTCCCACATCAGGCACATGAGTAACAAAGGCATGGAGCATCTGTACAGCATGAAGTGCAAGAACGTGGTGCCCCTCT
ATGACCTGCTGCTGGAGATGCTGGACGCCCACCGCCTACATGCGCCCACTAGCCGTGGAGGGGCATCCGTGGAGGAGACGGACCAAAGCCACTT
GGCCACTGCGGGCTCTACTTCATCGCATTCCTTGCAAAAGTATTACATCACGGGGGAGGCAGAGGGTTTCCCTGCCACGGTCTGAGGATCCAAA
A

pmirGLO_MCS
AAATCGAGATCTAATAAAATATCTTTATTTTCATTACATCTGTGTGTTGGTTTTTTGTGTGCATATGAAAAGAATTCAAAACCCGGGATGGAAGA
TGCCAAAAACATTAAGAAGGGCCCATCGAA

Supplementary Table S3. Clinical characteristics and PAM50 molecular subtypes of the SCAN-B breast tumour cohort. ER = Estrogen receptor alpha, PR = Progesterone receptor, HER2 = human epidermal growth factor receptor 2 (ERBB2), NA = not available.

**Receptor status**	**ER**	**PR**	**HER2**
Positive	2927 (84.2%)	2506 (72.1%)	462 (13.3%)
Negative	504 (14.5%)	923 (26.5%)	2861 (82.3%)
NA	47 (1.4%)	49 (1.4%)	155 (4.5%)
**Lymph node status**			
Positive	1240 (35.7%)		
Negative	2140 (61.5%)		
NA	98 (2.8%)		
**Grade**			
Grade 1	523 (15.0%)		
Grade 2	1638 (47.1%)		
Grade 3	1253 (36.0%)		
NA	64 (1.8%)		
**PAM50 molecular subtype**			
Luminal A	1754 (50.4%)		
Luminal B	399 (11.5%)		
HER2-enriched	355 (10.2%)		
Basal	330 (9.5%)		
Normal-like	640 (18.4%)		

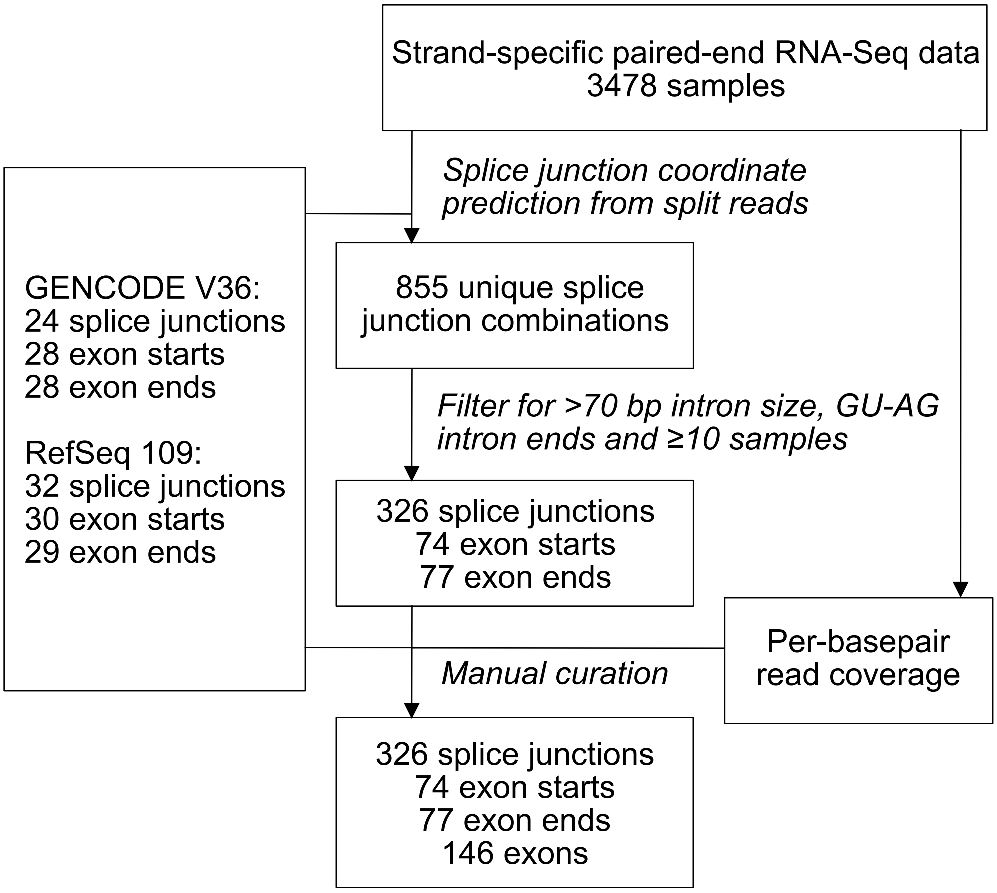

Supplementary Figure S1. Overview of the pipeline for identification of splice junctions and manual curation of exons. Exons from GENCODE V36 and RefSeq release 109 were included and only excluded when they were incompatible with GU-AG intron ends.

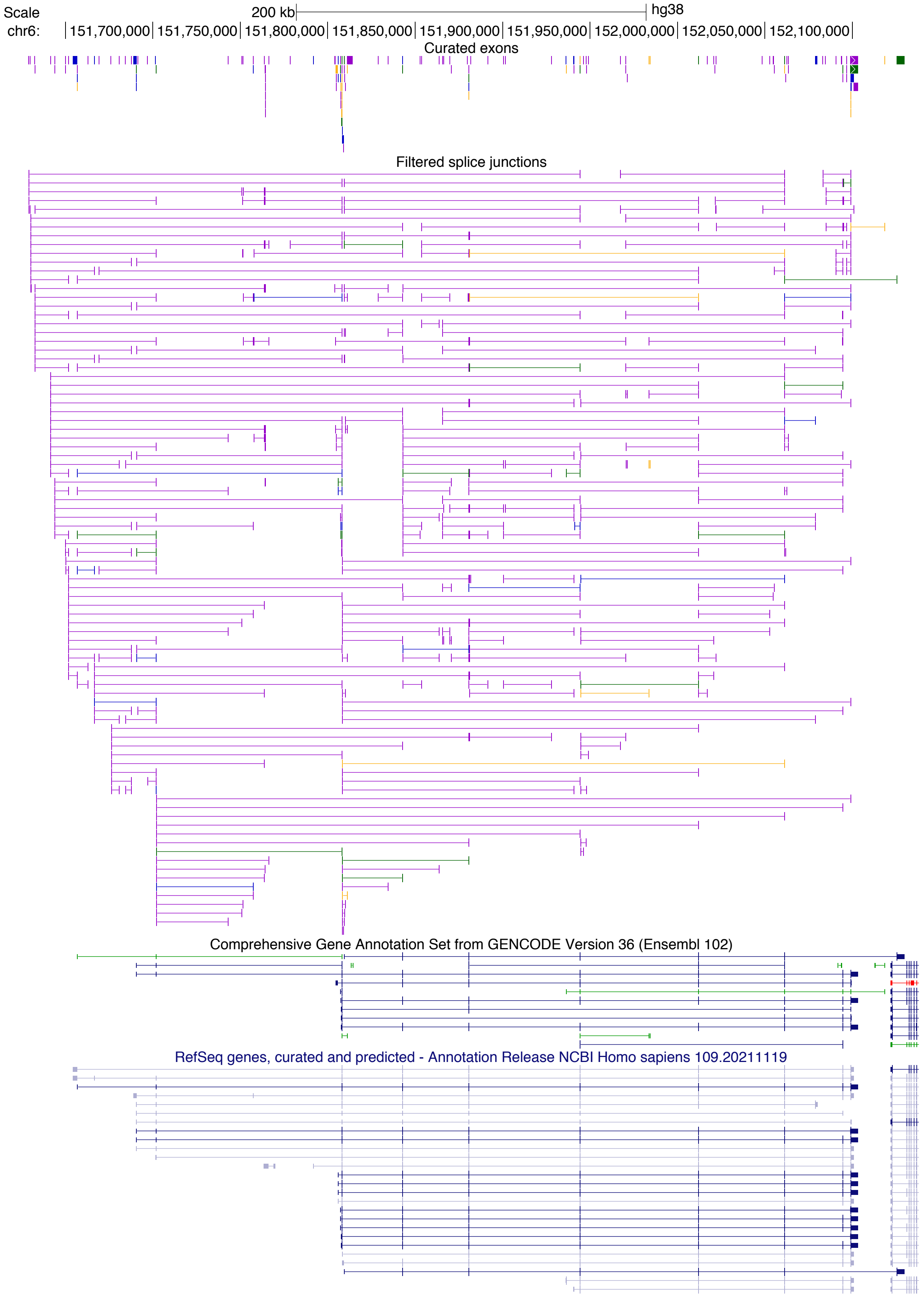

Supplementary Figure S2. Curated *ESR1* exons and the filtered set of splice junctions (green = annotated in GENCODE V36 and RefSeq release 109, yellow = GENCODE only, blue = RefSeq only, and purple = novel) together with GENCODE and RefSeq transcript annotation.

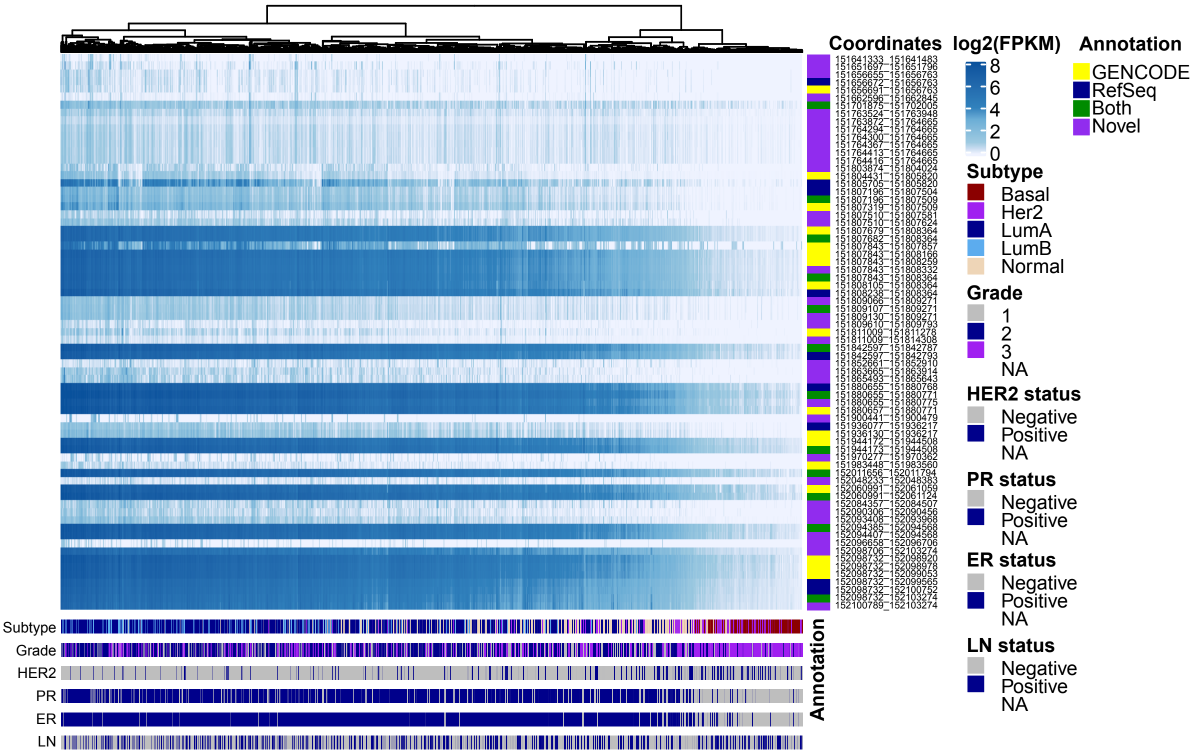

Supplementary Figure S3. Heatmap illustrating the expression of exons used in *ESR1* transcripts among 3478 breast tumours with exons sorted by increasing genomic coordinates from top to bottom. Only exons with an expression of at least 3 fragments per kilobase of exon model and million reads (fpkm) in at least 3 samples are shown. Tumours are labelled according to molecular subtype, grade and status for ER, progesterone receptor (PR), the tyrosine kinase receptor ERBB2 (also known as HER2) and positive lymph nodes at surgery (LN). ER, PR, and HER2 status were defined by immunohistochemistry. Exons are marked in green if they are annotated in both GENCODE and RefSeq databases, yellow for GENCODE only, blue for RefSeq only, and purple for novel exons.

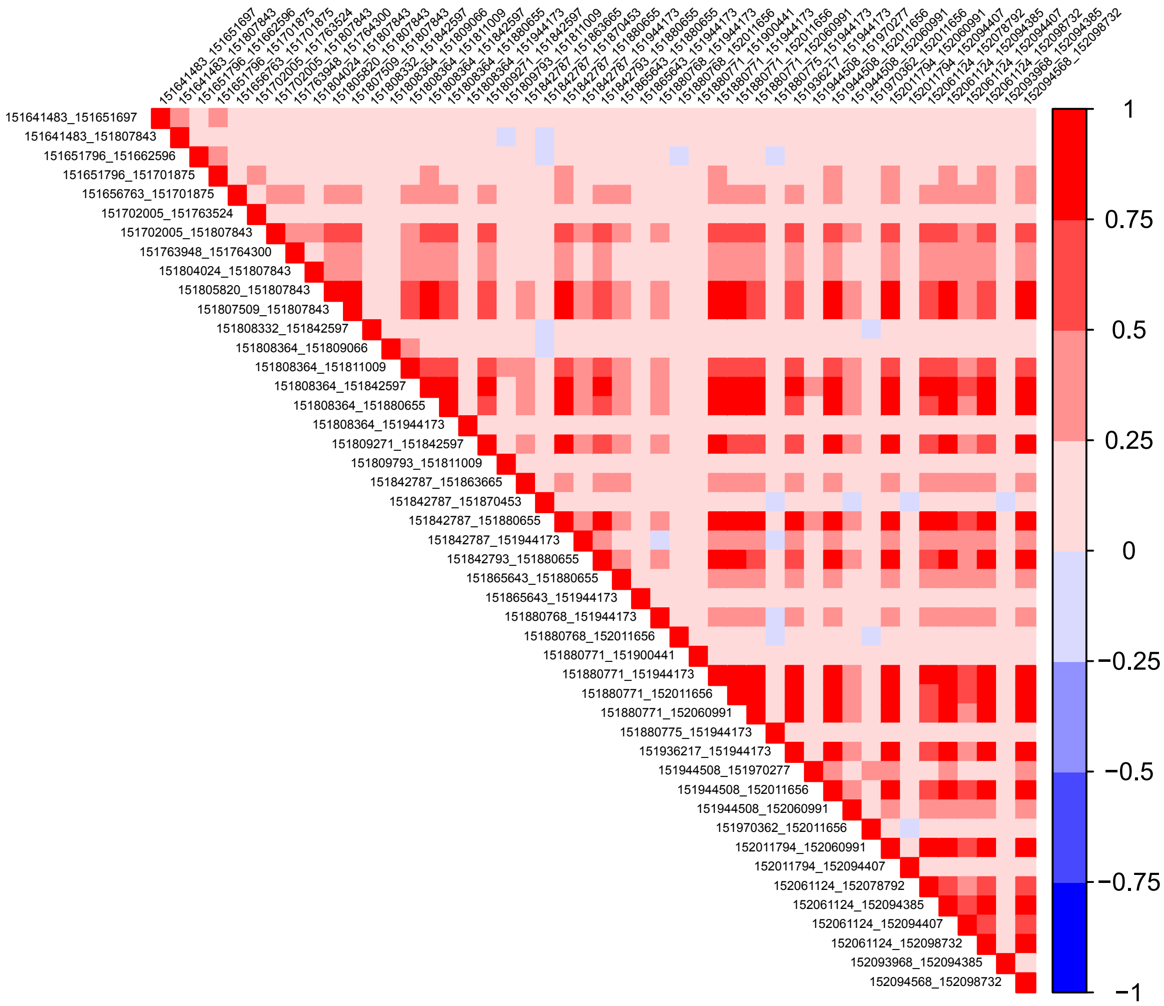

Supplementary Figure S4. Spearman rank correlation between splice junctions based on the expression in 3478 breast tumours from the SCAN-B cohort. Junctions are arranged by increasing genomic coordinates from top to bottom and left to right.

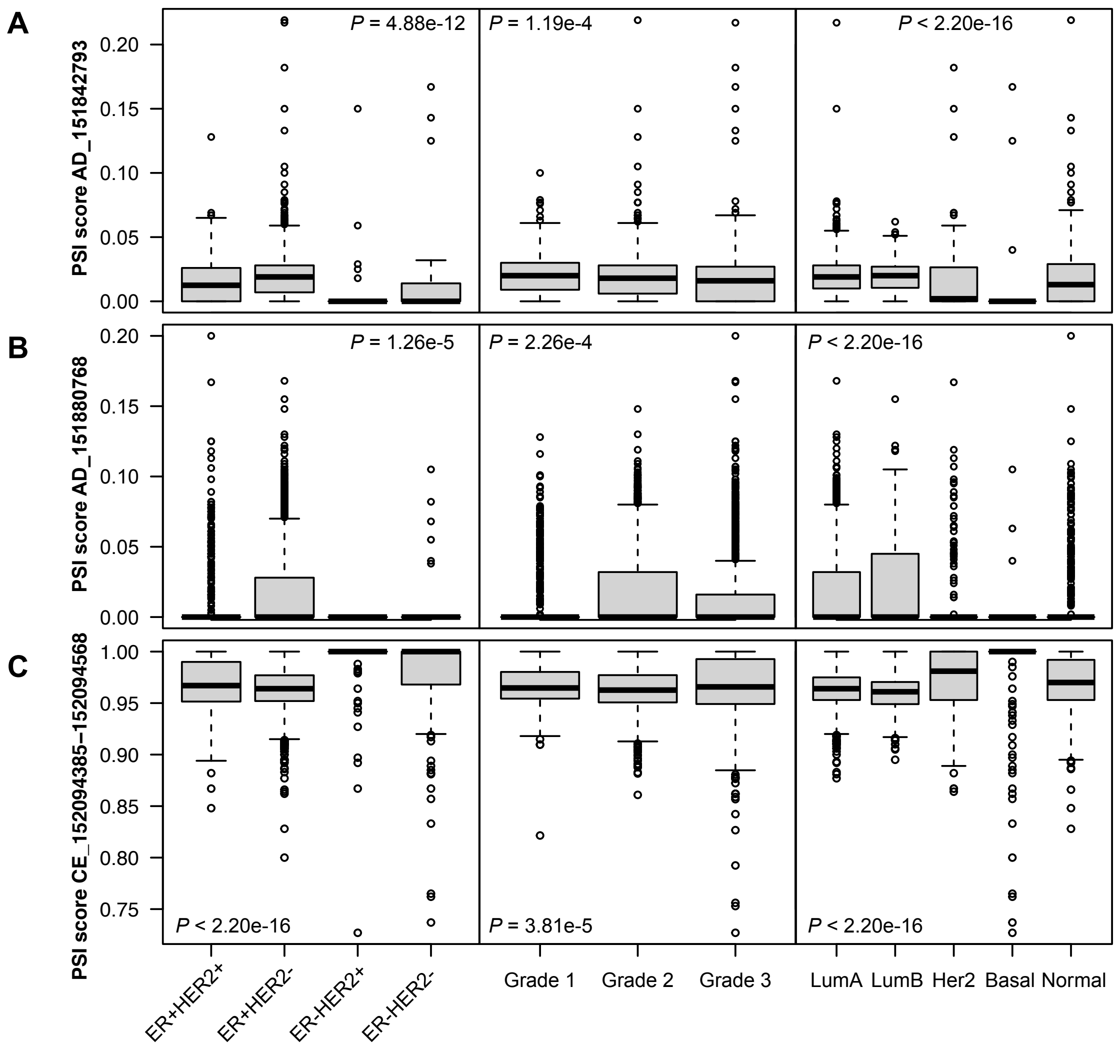

Supplementary Figure S5. Distribution of percent spliced-in index (PSI) scores across breast tumours divided by ER and HER2 receptor status, histological grade, and PAM50 molecular subtype. A PSI score of 1 means that a given exon, splice donor, or acceptor site is used in all transcripts, while lower values indicate alternative splicing. (A) An alternative donor site in exon 2 of the canonical full-length transcript leads to insertion of two amino acids in the DBD (see cds11 in Table 1) and had significantly higher PSI scores in HER2-negative *vs* HER2-positive samples ($p = 2.9 \times 10^{-2}$). (B) An alternative donor site in exon 3 leads to deletion of a single amino acid in the DBD (see cds16 in Table 1) and had higher PSI scores in ER-positive *vs* ER-negative tumours ($p = 9.5 \times 10^{-10}$) and in grade 2 vs grade 3 tumours ($p = 1.3 \times 10^{-3}$). (C) Exclusion of exon 7 leads to a frame-shift and produces a truncated protein (see cds1 in Table 1). It was more often excluded in ER-positive *vs* ER-negative tumours ($p = 2.5 \times 10^{-4}$). LumA = Luminal A, LumB = Luminal B, Her2 = HER2-enriched, Basal = Basal-like, and Normal = Normal-like subtypes. The Kruskal-Wallis rank sum test was used to calculate *P*-values for multi-group comparisons of PSI scores.

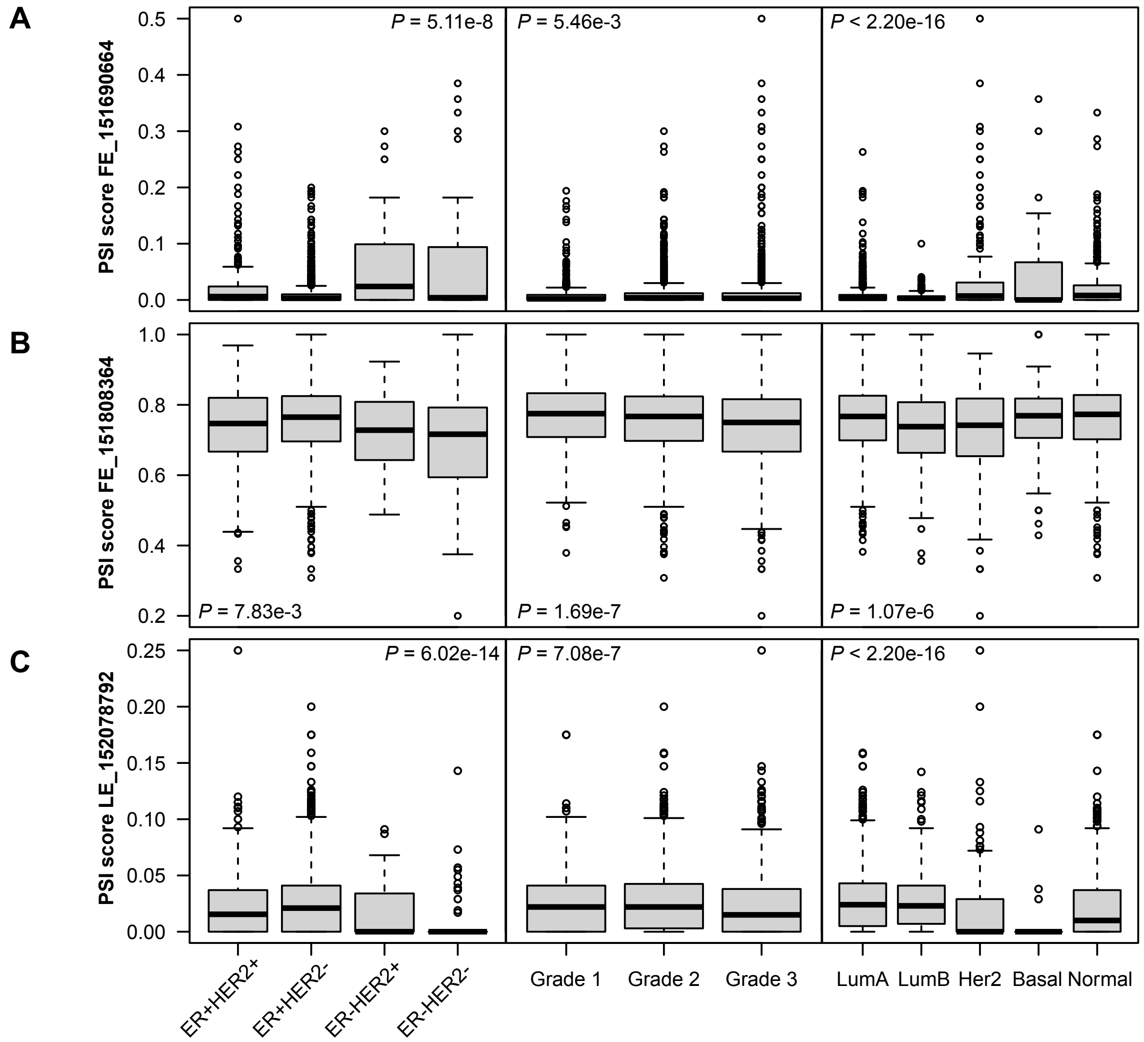

Supplementary Figure S6. Distribution of percent spliced-in index (PSI) scores across breast tumours divided by ER and HER2 receptor status, histological grade, and PAM50 molecular subtype. A PSI score of 1 means that a given exon, splice donor, or acceptor site is used in all transcripts, while lower values indicate alternative splicing. (A) An upstream first exon with significantly higher PSI scores in ER-negative vs ER-positive tumours (p = 4.3 x 10-5), in HER2-positive vs HER2-negative tumours (p = 3.3 x 10-6), and in grade 3 vs grade 1 and 2 tumours (p = 3.3 x 10-3 and 2.5 x 10-2, respectively). (B) The main first exon had significantly higher PSI scores in e.g. ER-positive vs ER-negative tumours (p = 6.8 x 10-3), in grade 1 vs grade 3 tumours (p = 1.0 x 10-7), and in tumours of the Luminal A vs Luminal B subtypes (p = 5.8 x 10-5). (C) An alternative last exon is located between exons 7 and 8 of the canonical full-length isoform and encodes a previously reported isoform with a truncated LBD. It had significantly higher PSI scores in ER-positive vs ER-negative tumours (p = 4.1 x 10-5) and in grade 1 vs grade 3 tumours (p = 3.2 x 10-3). LumA = Luminal A, LumB = Luminal B, Her2 = HER2-enriched, Basal = Basal-like, and Normal = Normal-like subtypes. The Kruskal-Wallis rank sum test was used to calculate *P*-values for multi-group comparisons of PSI scores.

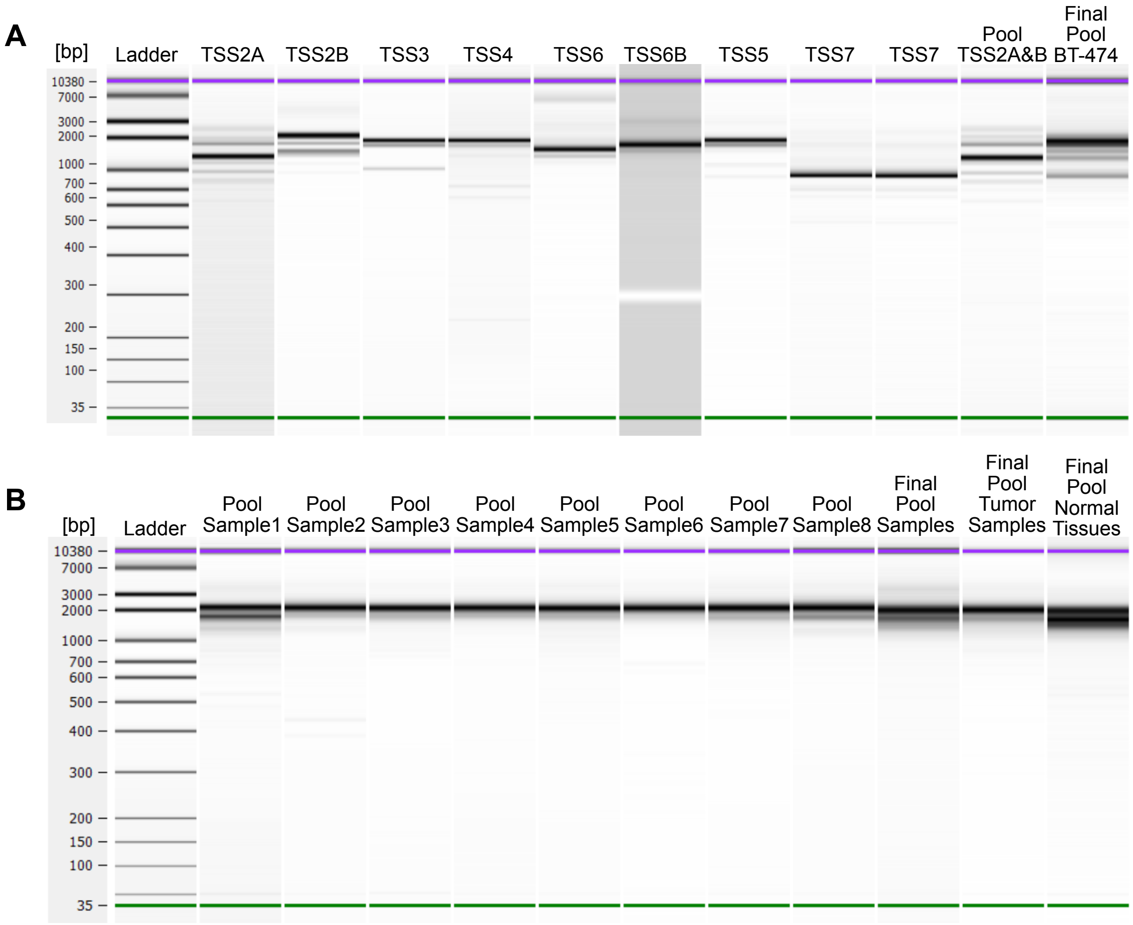

Supplementary Figure S7. Validation of RT-PCR primers for long-read sequencing. (A) Bioanalyzer gel-like view showing amplification and the size range of products from primer pairs targeting different first exons in the BT-474 cell line. Last lane: Amplicon library pool for BT-474. (B) Amplicon library pools for 8 individual tumour samples and the final, barcoded pools for tumours and normal tissues.

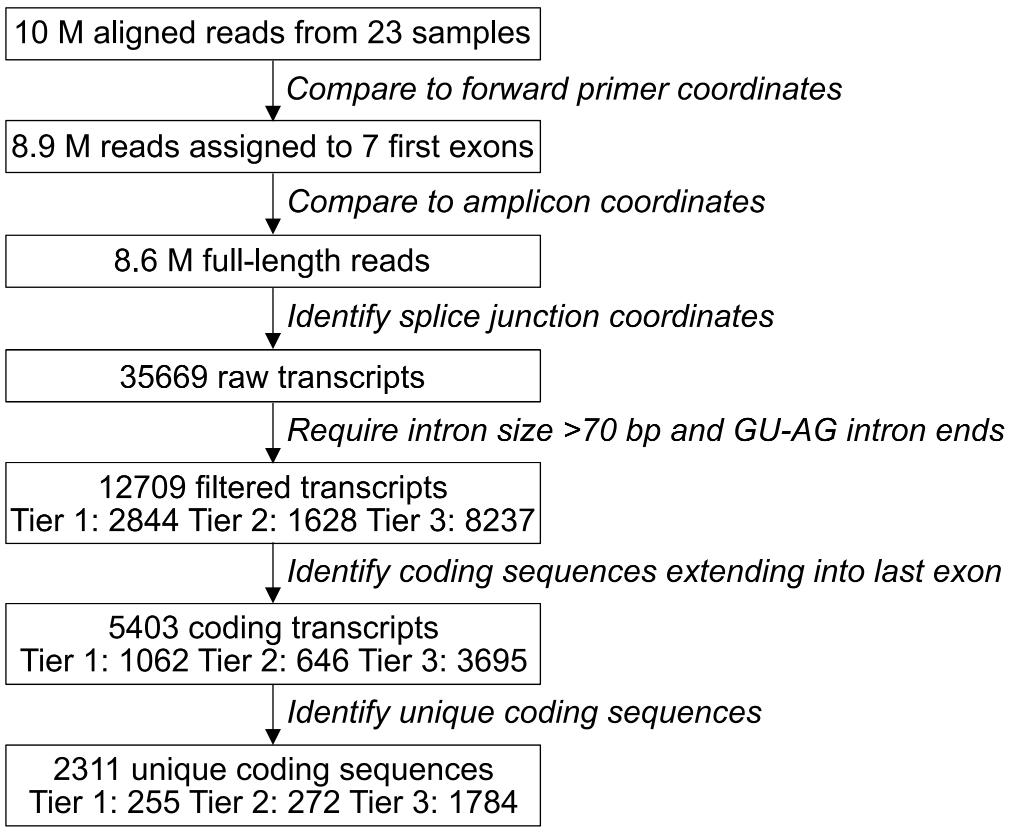

Supplementary Figure S8. Annotation of transcripts and protein-coding sequences from long-read sequencing data. Aligned full-length reads spanning from forward to reverse primer were compared to the curated sets of exons and splice junctions and transcripts were divided into three tiers depending on the available support: Tier 1 isoforms only have exon ends present in the curated set of exons from SCAN-B breast tumours, GENCODE and RefSeq, tier 2 isoforms have one or more exon ends that are only present in the unfiltered SCAN-B junction set and isoforms in tier 3 have one or more exon ends that have not been supported by new or existing annotation.

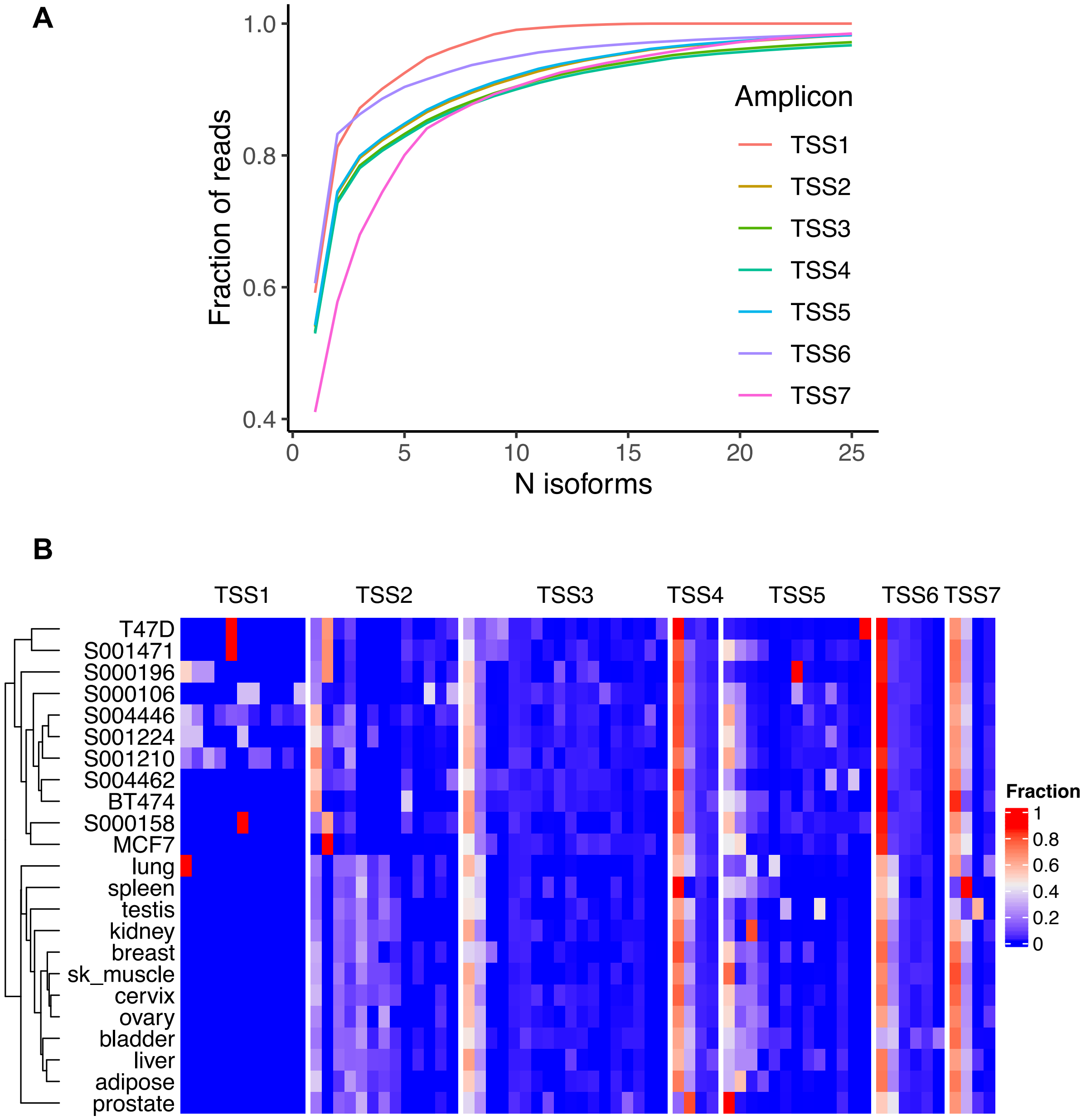

Supplementary Figure S9. (A) Isoform complexity among transcripts resulting from each first exon illustrated as the fraction of all full-length reads that is explained by a given number of mRNA isoforms. (B) Heatmap with the expression of the most common mRNA isoforms for each first exon shown as the fraction of full-length reads for that amplicon for the sequenced breast tumours, breast cancer cell lines and normal tissues. TSS = transcription start site, sk_muscle = skeletal muscle.

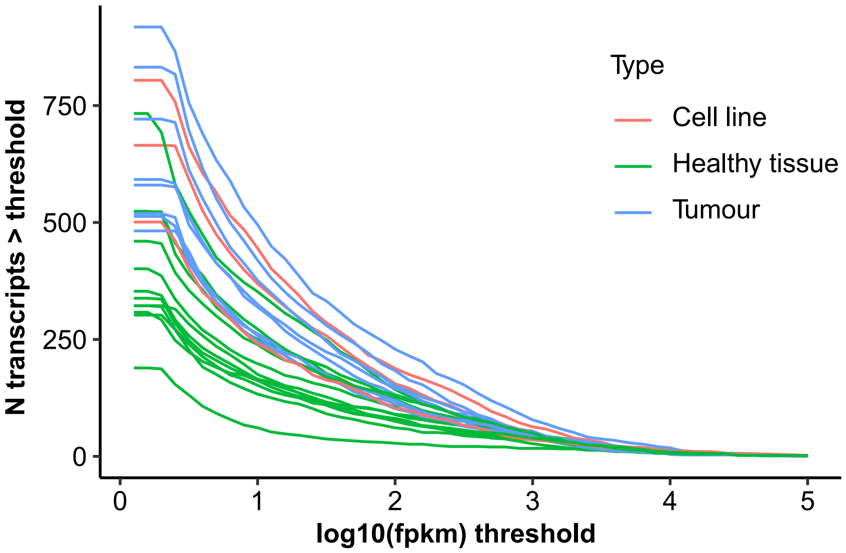

Supplementary Figure S10. Total transcript isoform complexity in the long-read sequencing data for samples coloured by origin (Cell line = BT-474, MCF7, T47D; Healthy tissue = adipose, bladder, breast, cervix, kidney, liver, lung, ovary, prostate, skeletal muscle, spleen, testis; Tumour = breast tumours).

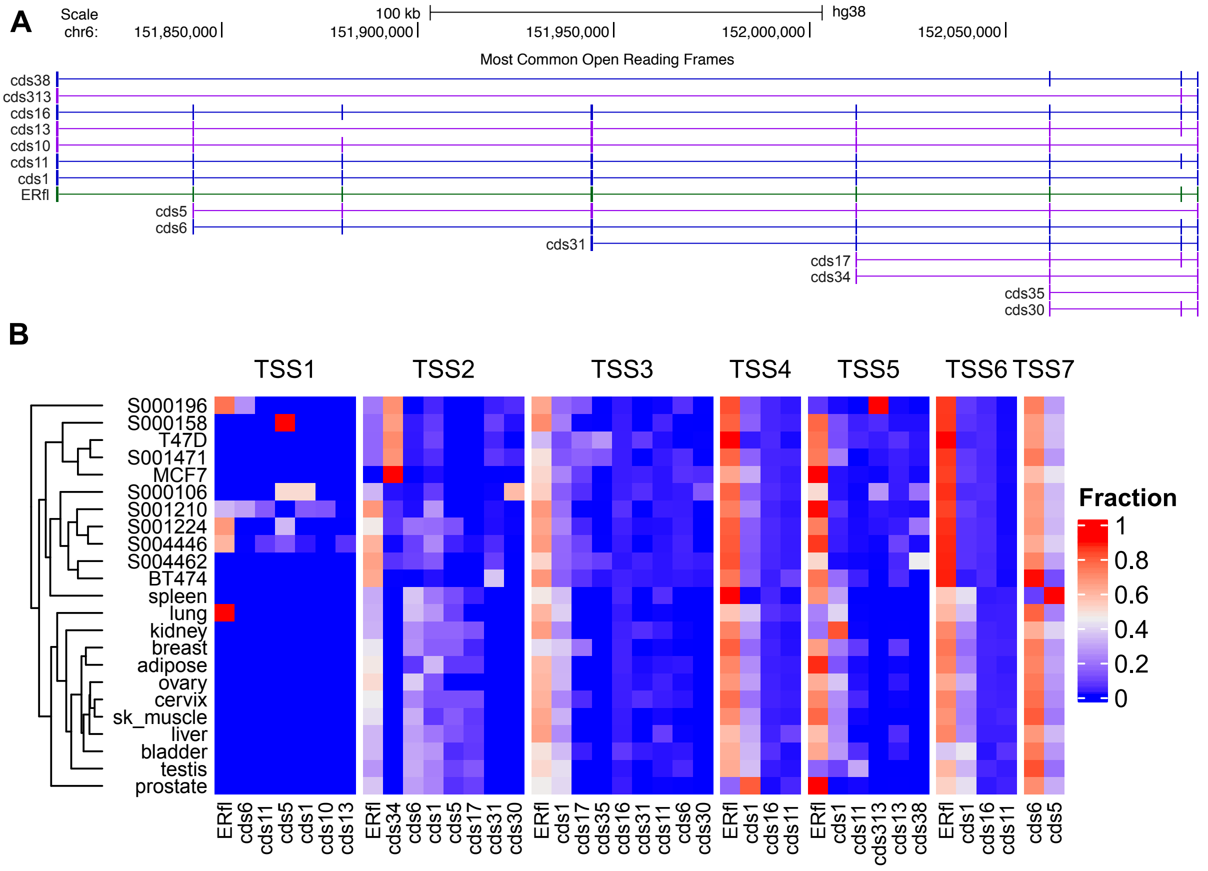

Supplementary Figure S11. Open reading frames of the most common tier 1 protein isoforms for each targeted first exon (TSS1-7, A). These isoforms together encompass 80% of the full-length reads for a primer pair. Heatmap of the estimated relative expression for these isoforms in the sequenced breast tumours, breast cancer cell lines, and normal tissues (B). The expression was estimated by combining the counts for all transcripts that encode a given amino acid sequence and expressed as the fraction of the total number of full-length reads for a primer pair.

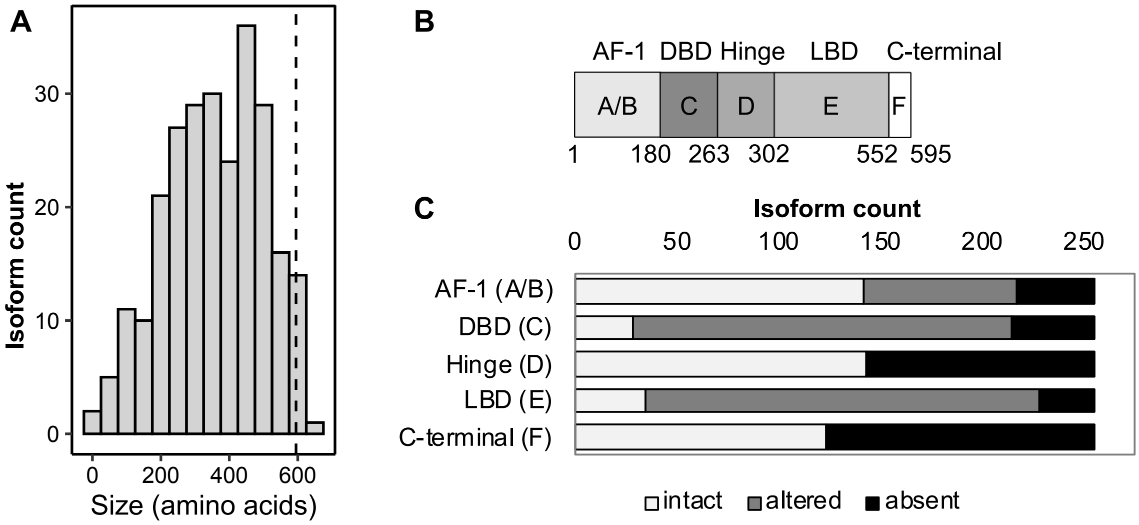

Supplementary Figure S12. (A) Size distribution of the predicted protein isoforms from tier 1 transcripts (transcripts where all exon ends are present in the curated set combining known and novel exons and splice junctions). The full-length isoform of ER is indicated at 595 amino acids. (B) Domain structure of the ER with amino acid positions from (53). (C) Effects on the different domains of the ER for tier 1 isoforms. AF-1 = activating function 1, DBD = DNA-binding domain, LBD = ligand-binding domain.

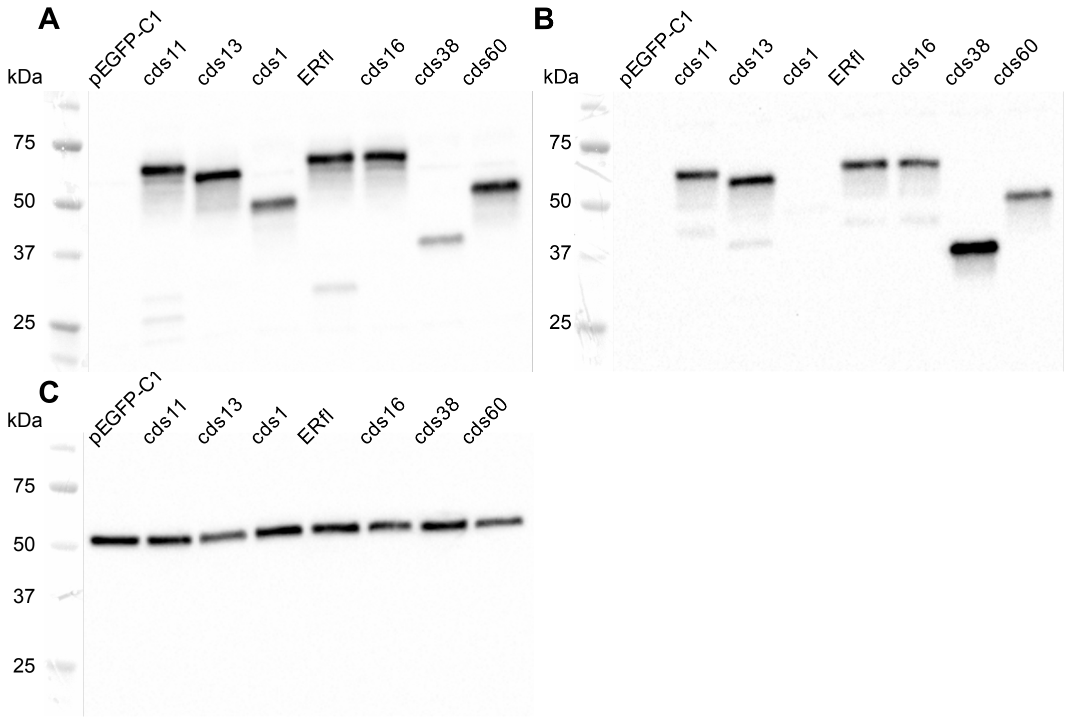

Supplementary Figure S13. Western blots showing validation of protein expression and expected sizes for the cloned full-length and alternative isoforms using (A) an N-terminal or (B) a C-terminal anti-ESR1 antibody. (C) Tubulin is included as a loading control. pEGFP-C1 = empty pEGFP-C1 vector, ERfl = full-length estrogen receptor alpha (ER), kDa = kilo Dalton.

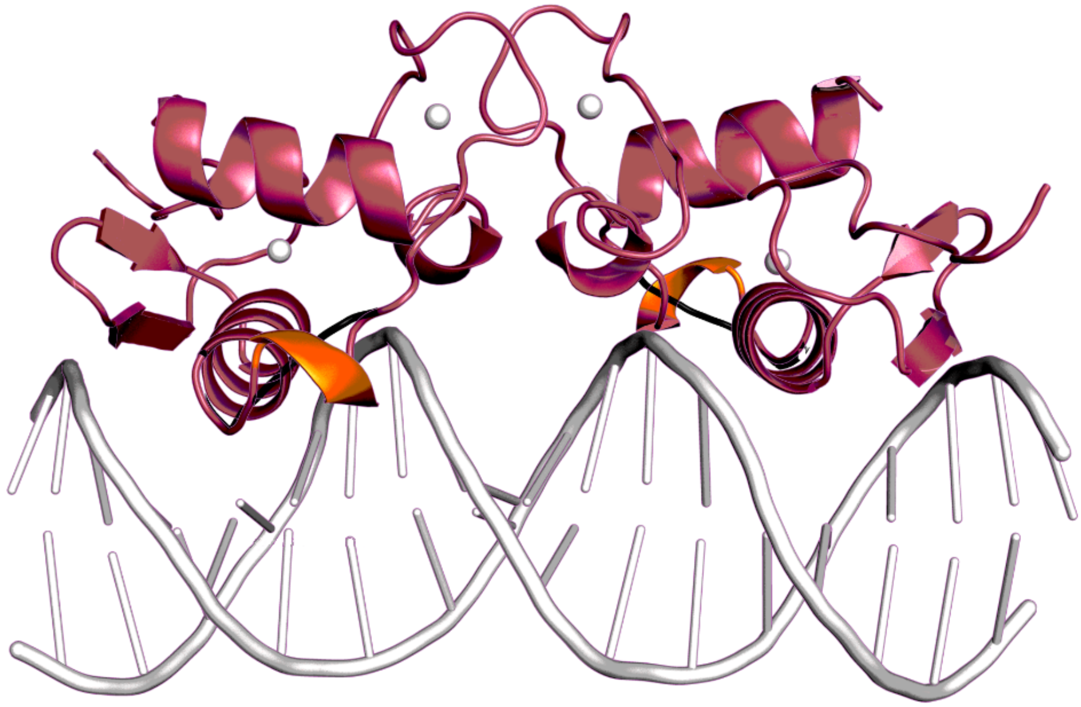

Supplementary Figure S14. Homology modelling of the estrogen receptor alpha DNA-binding domain for the cds11 isoform dimer (SWISS-MODEL) based on PDB 1hcq.2. Red: Structurally overlapping domains. Orange: Amino acids only present in cds11. Light grey spheres: Zinc atoms. Light grey: Double-stranded DNA.

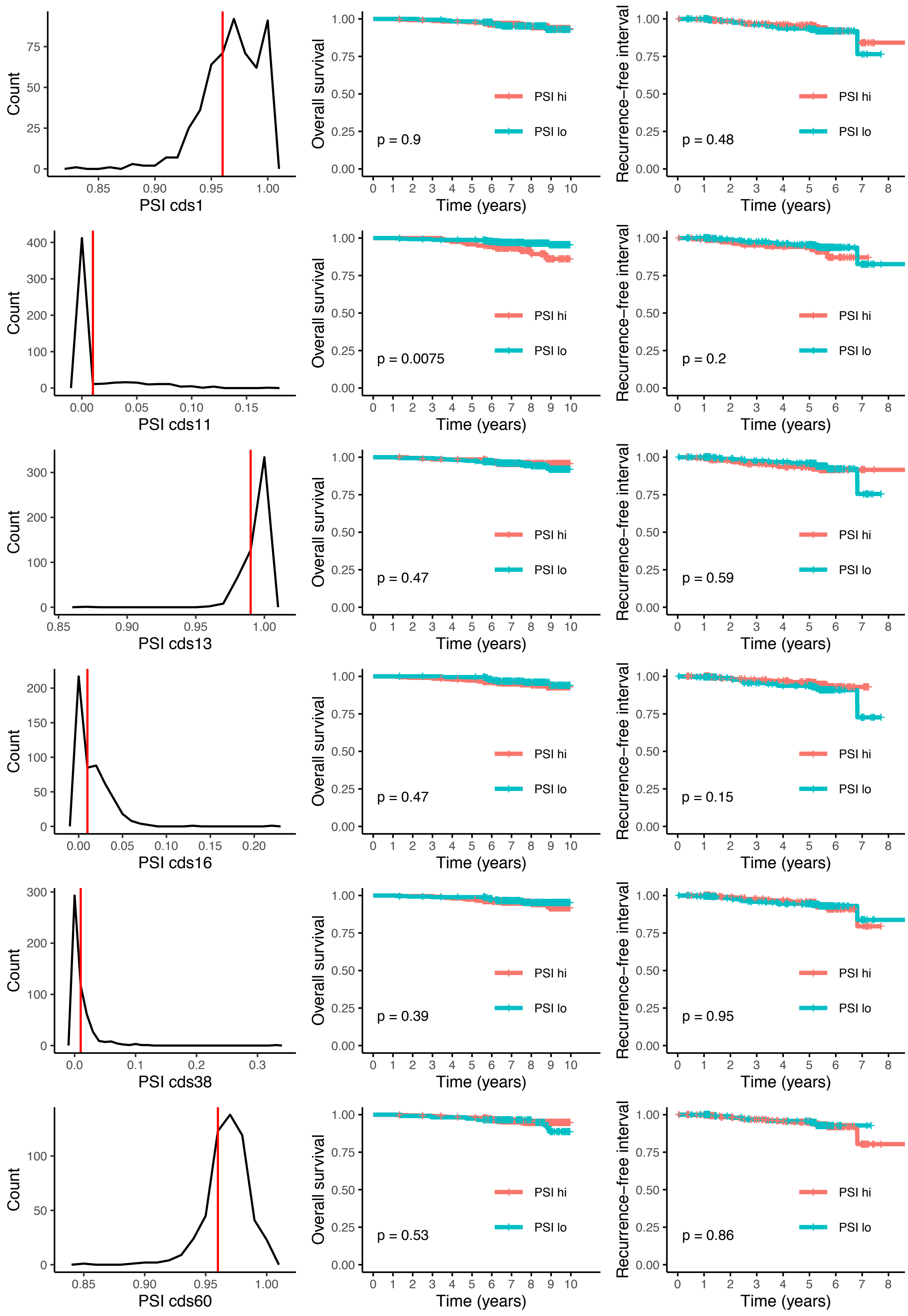

Supplementary Figure S15. PSI score distribution for alternative isoforms (vertical red line marks cut-off for low or high score), overall survival, and recurrence-free interval for premenopausal women with ER-positive tumours who received endocrine therapy.

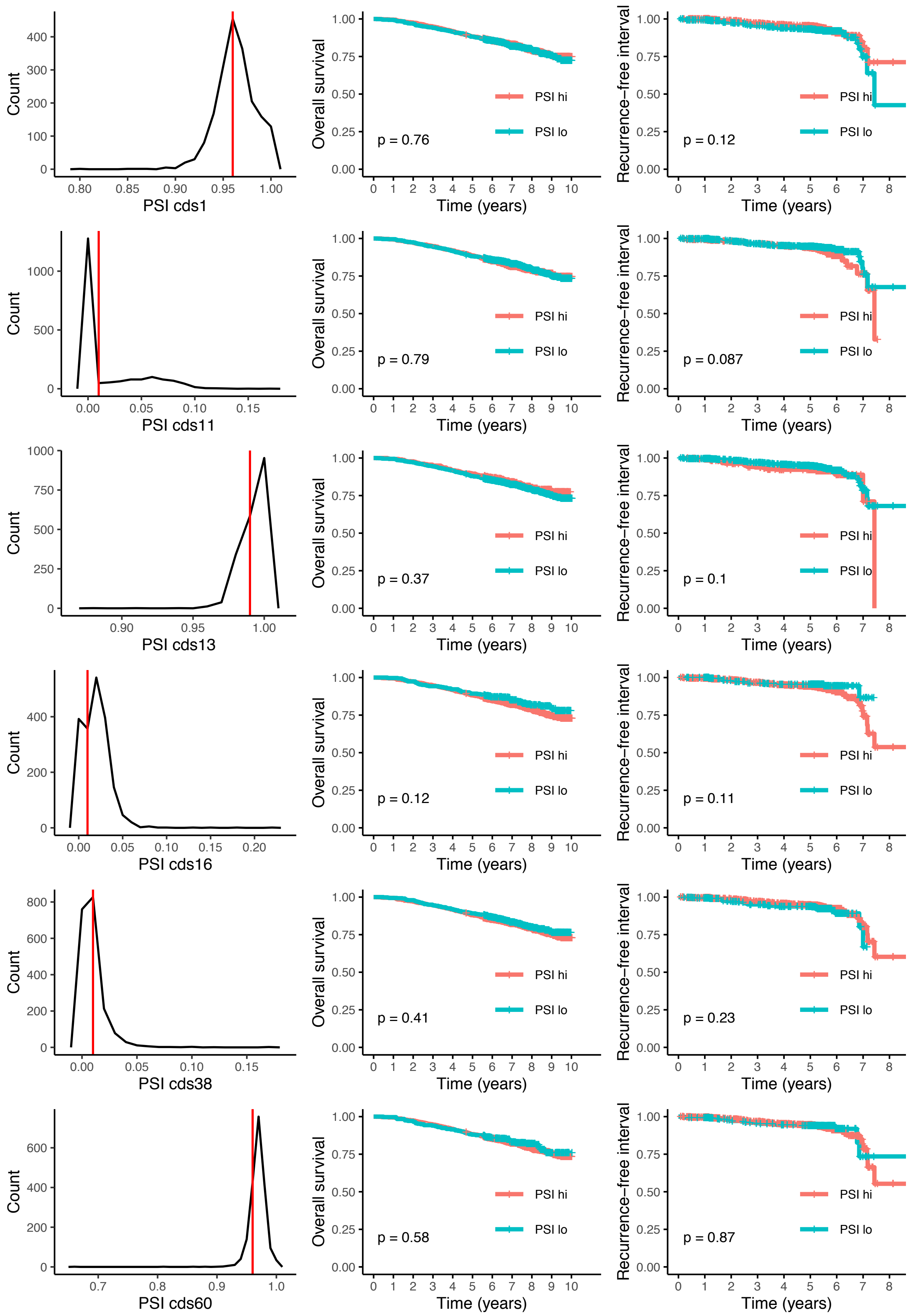

Supplementary Figure S16. PSI score distribution for alternative isoforms (vertical red line marks cut-off for low or high score), overall survival, and recurrence-free interval for postmenopausal women with ER-positive tumours who received endocrine therapy.

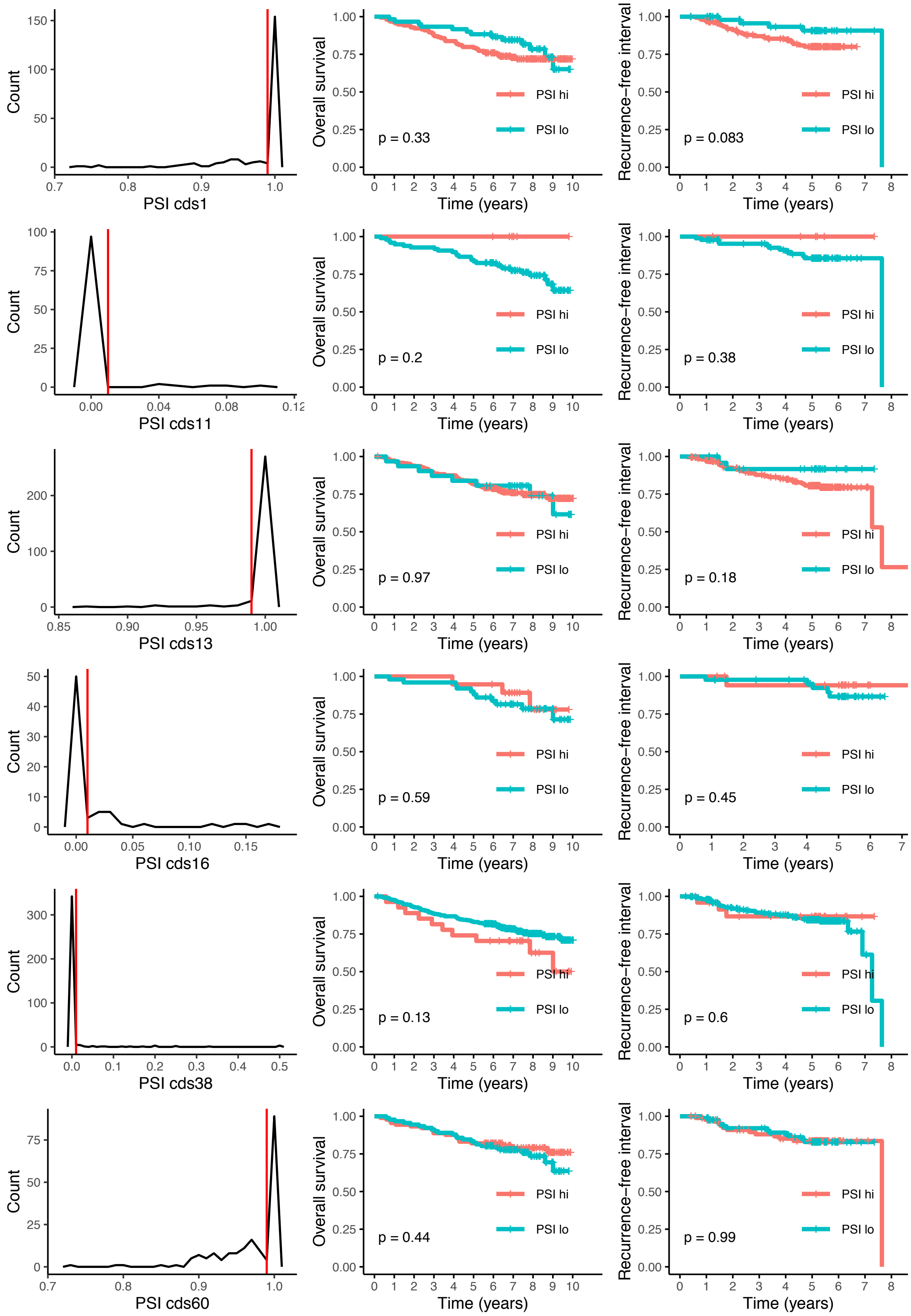

Supplementary Figure S17. PSI score distribution for alternative isoforms (vertical red line marks cut-off for low or high score), overall survival, and recurrence-free interval for women with ER-negative tumours.

Supplementary Table S5. Univariable and multivariable Cox proportional hazards regression analysis for overall survival (OAS) in patient groups divided by isoform expression. PSI lo and PSI hi corresponds to the number of patients in each group. The hazard ratios are shown with 95% confidence intervals within parenthesis, followed by *P*-values. The multivariable analysis included patient lymph node status (positive or negative), histological grade (1, 2, or 3), tumour size (≤20 mm or >20 mm), and for ER-negative patients, patient age (<50 years or ≥50 years).

Isoform	PSI lo	PSI hi	OAS univariable	OAS multivariable
ER-positive, endocrine therapy, premenopausal				
cds1	182	353	1.05 (0.47-2.36) P=0.90	1.13 (0.41-3.07) P=0.82
cds11	409	119	0.36 (0.16-0.79) P=0.01	0.51 (0.19-1.39) P=0.19
cds13	333	203	1.36 (0.59-3.12) P=0.47	1.33 (0.47-3.82) P=0.59
cds16	205	321	0.73 (0.32-1.70) P=0.47	0.84 (0.31-2.29) P=0.73
cds38	280	255	0.71 (0.32-1.56) P=0.39	0.82 (0.31-2.12) P=0.68
cds60	115	417	1.31 (0.55-3.13) P=0.54	1.17 (0.38-3.58) P=0.79
ER-positive, endocrine therapy, postmenopausal				
cds1	788	1144	1.03 (0.84-1.26) P=0.76	1.02 (0.83-1.25) P=0.88
cds11	1255	664	0.97 (0.79-1.20) P=0.79	0.95 (0.77-1.18) P=0.67
cds13	1614	318	1.13 (0.86-1.50) P=0.37	1.26 (0.94-1.69) P=0.13
cds16	346	1565	0.81 (0.61-1.06) P=0.12	0.74 (0.55-0.98) P=0.04
cds38	535	1400	0.91 (0.73-1.14) P=0.41	0.88 (0.70-1.12) P=0.30
cds60	316	1613	0.93 (0.70-1.22) P=0.58	0.96 (0.72-1.26) P=0.75
ER-negative tumours				
cds1	60	154	0.73 (0.39-1.37) P=0.33	0.61 (0.29-1.27) P=0.19
cds11	97	6	NA	NA
cds13	31	265	1.02 (0.49-2.12) P=0.97	0.93 (0.42-2.04) P=0.85
cds16	50	19	1.42 (0.4-5.11) P=0.59	3.12 (0.37-26.03) P=0.29
cds38	341	27	0.61 (0.31-1.17) P=0.14	0.56 (0.28-1.13) P=0.11
cds60	91	89	1.27 (0.69-2.32) P=0.44	1.40 (0.74-2.65) P=0.31

Supplementary Table S6. Univariable and multivariable Cox proportional hazards regression analysis for recurrence-free interval (RFI) in patient groups divided by isoform expression. PSI lo and PSI hi corresponds to the number of patients in each group. The hazard ratios are shown with 95% confidence intervals within parenthesis, followed by *P*-values. The multivariable analysis included patient lymph node status (positive or negative), histological grade (1, 2, or 3), tumour size (≤20 mm or >20 mm), and for ER-negative patients, patient age (<50 years or ≥50 years).

**Isoform**	**PSI lo**	**PSI hi**	**RFI univariable**	**RFI multivariable**
ER-positive, endocrine therapy, premenopausal				
cds1	182	353	1.29 (0.64-2.6) P=0.48	1.29 (0.59-2.8) P=0.52
cds11	409	119	0.6 (0.28-1.33) P=0.21	0.58 (0.25-1.33) P=0.20
cds13	333	203	0.82 (0.4-1.67) P=0.59	0.89 (0.4-1.97) P=0.78
cds16	205	321	1.69 (0.82-3.47) P=0.15	1.64 (0.75-3.57) P=0.21
cds38	280	255	0.98 (0.48-1.98) P=0.95	1 (0.47-2.14) P=0.99
cds60	115	417	0.92 (0.38-2.26) P=0.86	0.97 (0.37-2.6) P=0.96
ER-positive, endocrine therapy, postmenopausal				
cds1	788	1144	1.33 (0.93-1.92) P=0.12	1.36 (0.94-1.97) P=0.10
cds11	1255	664	0.73 (0.5-1.05) P=0.09	0.77 (0.53-1.12) P=0.17
cds13	1614	318	0.69 (0.45-1.07) P=0.10	0.76 (0.48-1.21) P=0.25
cds16	346	1565	0.64 (0.37-1.11) P=0.11	0.64 (0.37-1.10) P=0.11
cds38	535	1400	1.27 (0.86-1.88) P=0.23	1.25 (0.84-1.88) P=0.27
cds60	316	1613	0.96 (0.59-1.57) P=0.87	0.88 (0.52-1.47) P=0.62
ER-negative tumours				
cds1	60	154	0.41 (0.14-1.17) P=0.09	0.41 (0.12-1.38) P=0.15
cds11	97	6	NA	NA
cds13	31	265	0.39 (0.09-1.61) P=0.19	0.22 (0.03-1.62) P=0.14
cds16	50	19	2.24 (0.26-19.18) P=0.46	NA
cds38	341	27	1.37 (0.42-4.45) P=0.60	1.62 (0.38-6.89) P=0.52
cds60	91	89	1 (0.45-2.22) P=0.99	0.97 (0.42-2.27) P=0.95

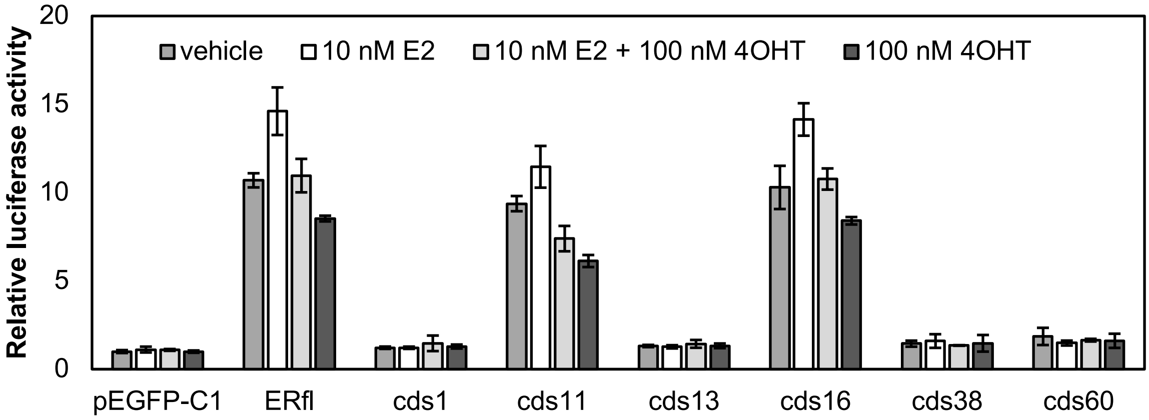

Supplementary Figure S18. Isoform transcription factor activity for a dual-luciferase reporter with firefly luciferase controlled by the ERE-containing C3 promoter. Luminescence from firefly luciferase was normalised to *Renilla* luciferase and the activity was expressed relative to the baseline value for the empty pEGFP-C1 vector with vehicle. ERfl = full-length estrogen receptor alpha (ER), vehicle control = 0.1% ethanol, E2 = estradiol, and 4OHT = 4-hydroxytamoxifen. Bars show the mean of n = 3 replicates ± standard deviation.

A

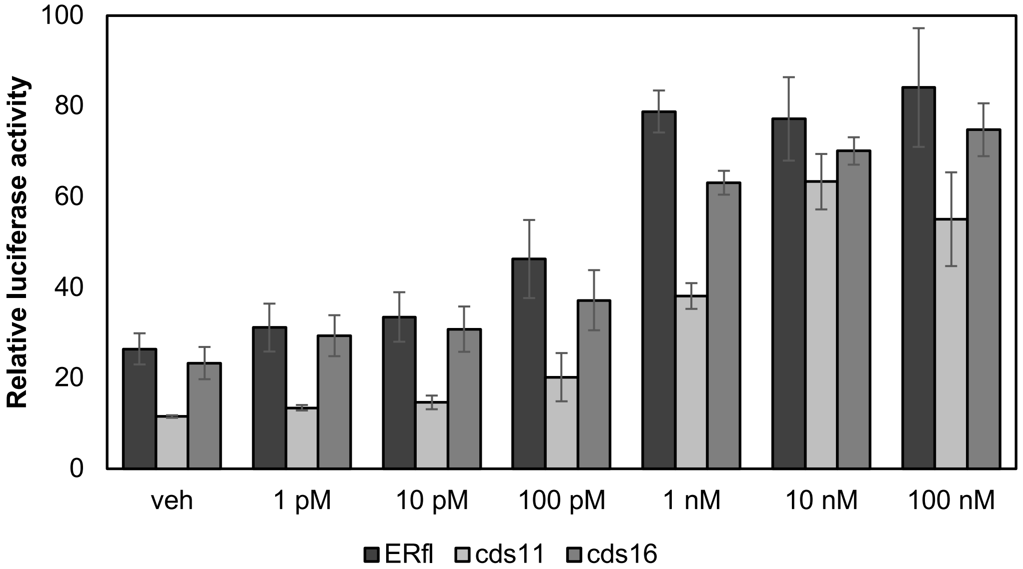

B

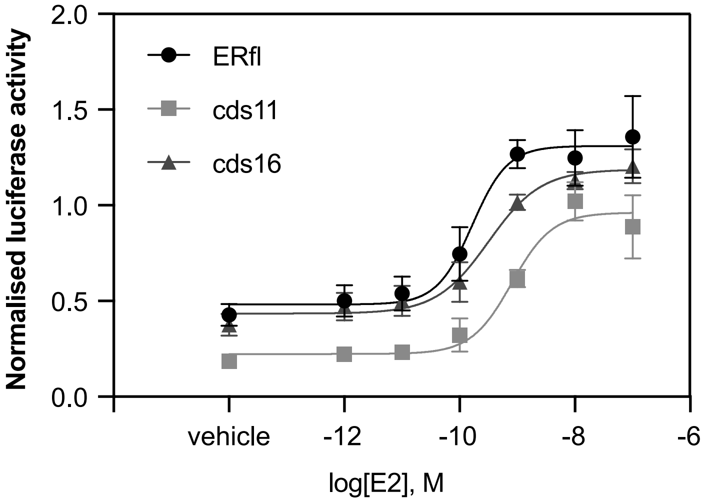

Supplementary Figure S19. Dose-response analysis of two alternative isoforms, cds11 and cds16, compared with the full-length protein, ERfl with concentrations of estradiol (E2) ranging from 1 pM to 100 nM. (A) Firefly luciferase activity for the pmirGLO-3xERE construct relative to cells transfected with pEGFP-C1 and treated with vehicle (0.1% ethanol). (B) Dose-response curves illustrating the differences between isoforms. ERfl = full-length estrogen receptor alpha (ER), vehicle control = 0.1% ethanol, E2 = estradiol, and 4OHT = 4-hydroxytamoxifen. Bars show the mean of n = 3 replicates ± standard deviation.

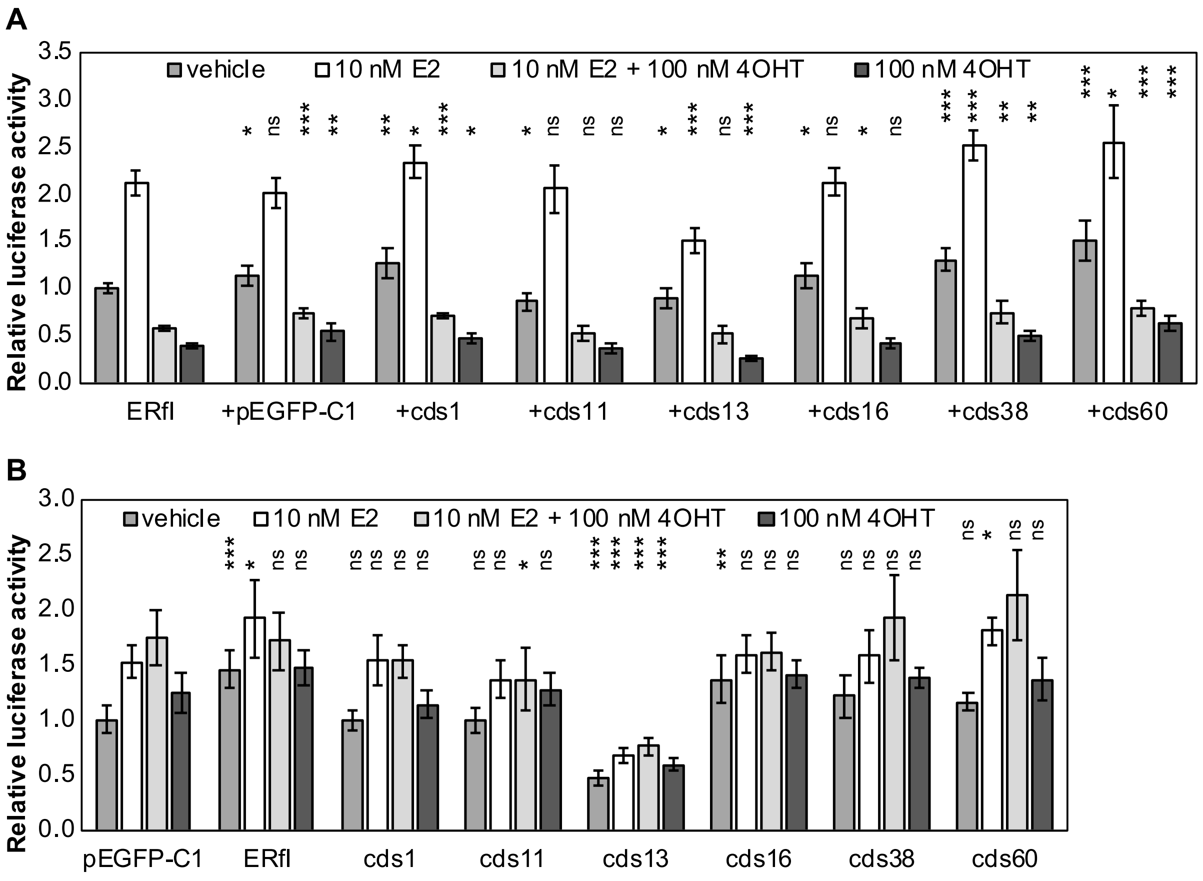

Supplementary Figure S20. (A) Transcription factor activity on a dual-luciferase reporter with firefly luciferase controlled by a 3xERE-containing promoter upon co-transfection in ER-negative HepG2 cells of full-length ER together with empty pEGFP-C1 or one of the alternative ER isoforms as indicated. Luminescence from firefly luciferase was normalised to *Renilla* luciferase and the activity was expressed relative to the baseline value for only full-length ER with vehicle. Two-tailed Student's *t*-test was used to test for significant differences in relative luciferase activity between each isoform and ERfl matched by treatment. (B) Transcription factor activity after transfection of empty pEGFP-C1, full-length ER or one of the alternative isoforms in ER-positive MCF7 cells. Normalised activity was expressed relative to the baseline value for the empty pEGFP-C1 vector with vehicle. Two-tailed Student's *t*-test was used to test for significant differences in relative luciferase activity between each isoform and empty pEGFP-C1 matched by treatment. * $p < 0.05$, ** $p < 0.01$, *** $p < 0.001$, and ns = non-significant. ERfl = full-length estrogen receptor alpha (ER), vehicle control = 0.1% ethanol, E2 = estradiol, and 4OHT = 4-hydroxytamoxifen. Bars indicate the mean of n = 6 replicates ± standard deviation.

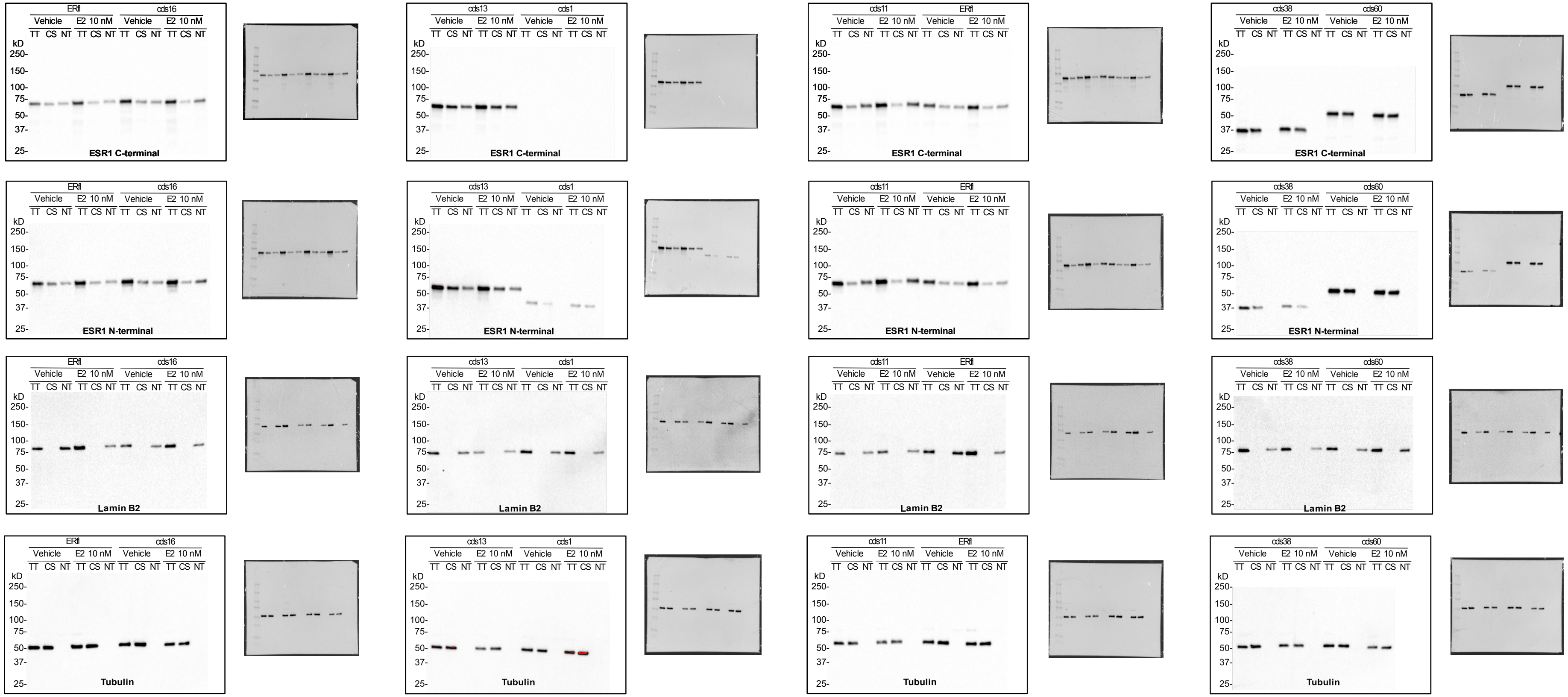

Supplementary Figure S21. Subcellular fractionation followed by western blotting to determine the relative distribution between cytoplasm and nucleus for each isoform; uncropped blots and overlay with size marker for subcellular fractionation experiments included in Figure 9A-D. Isoform and antibody are marked in each panel.

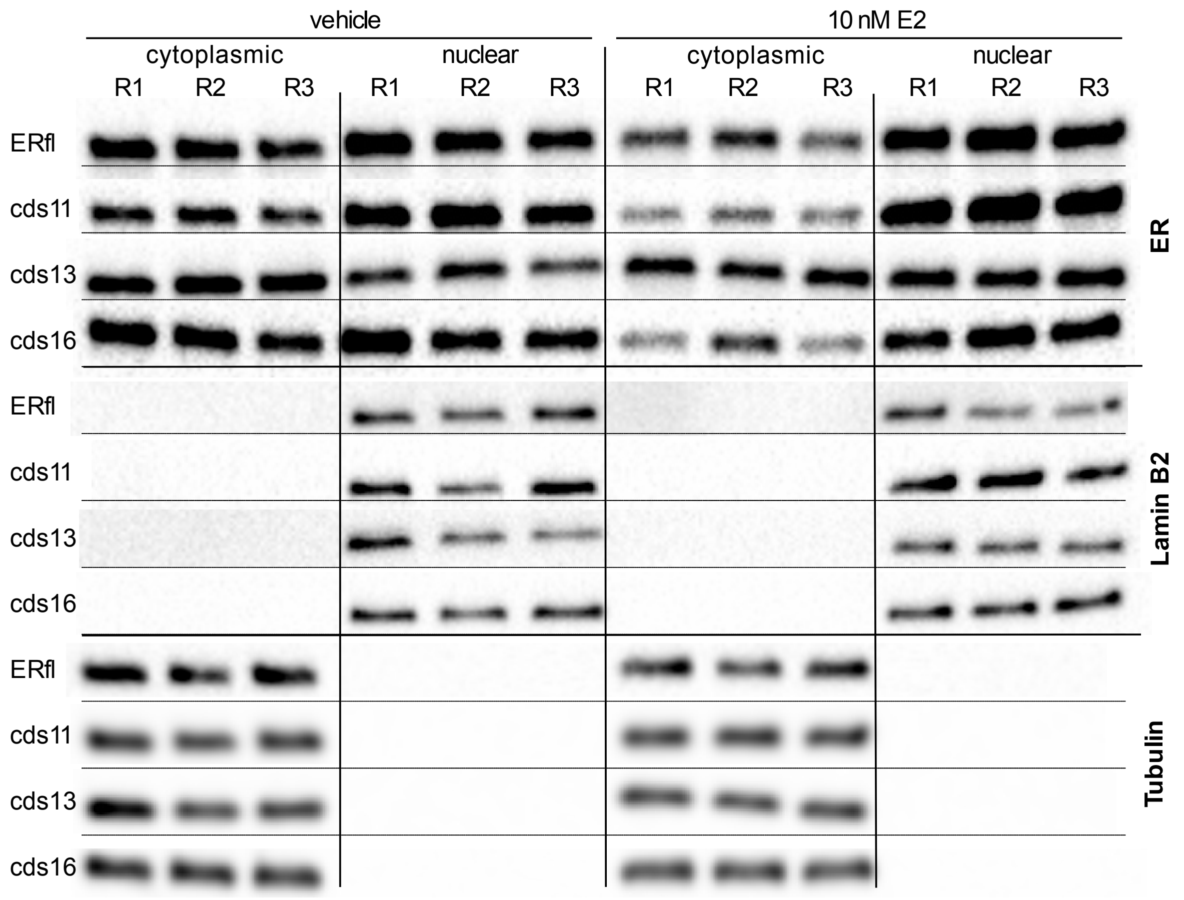

Supplementary Figure S22. Subcellular fractionation followed by western blotting using an N-terminal anti-ESR1 antibody to determine the relative distribution between cytoplasm and nucleus for each isoform after a 30 min treatment with vehicle or 10 nM E2. Lamin B2 and tubulin were included as controls for the purity of the nuclear and cytoplasmic fractions, respectively. Vehicle control = 0.1% ethanol, E2 = estradiol, ERfl = full-length estrogen receptor alpha (ER), n = 3 replicates.

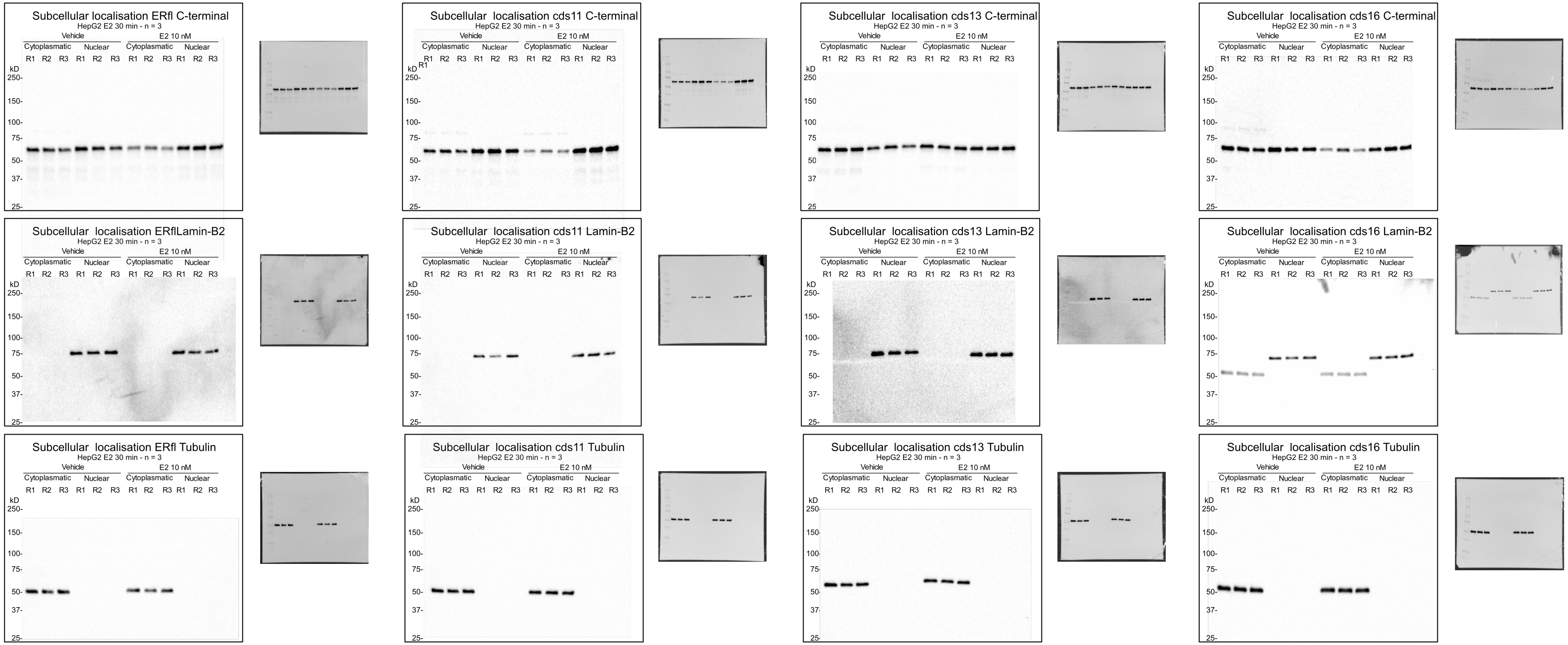

Supplementary Figure S23. Subcellular fractionation with replicates followed by western blotting to determine the relative distribution between cytoplasm and nucleus for each isoform; uncropped blots and overlay with size marker for subcellular fractionation experiments used in Figure 9E. Isoform and antibody are marked in each panel.

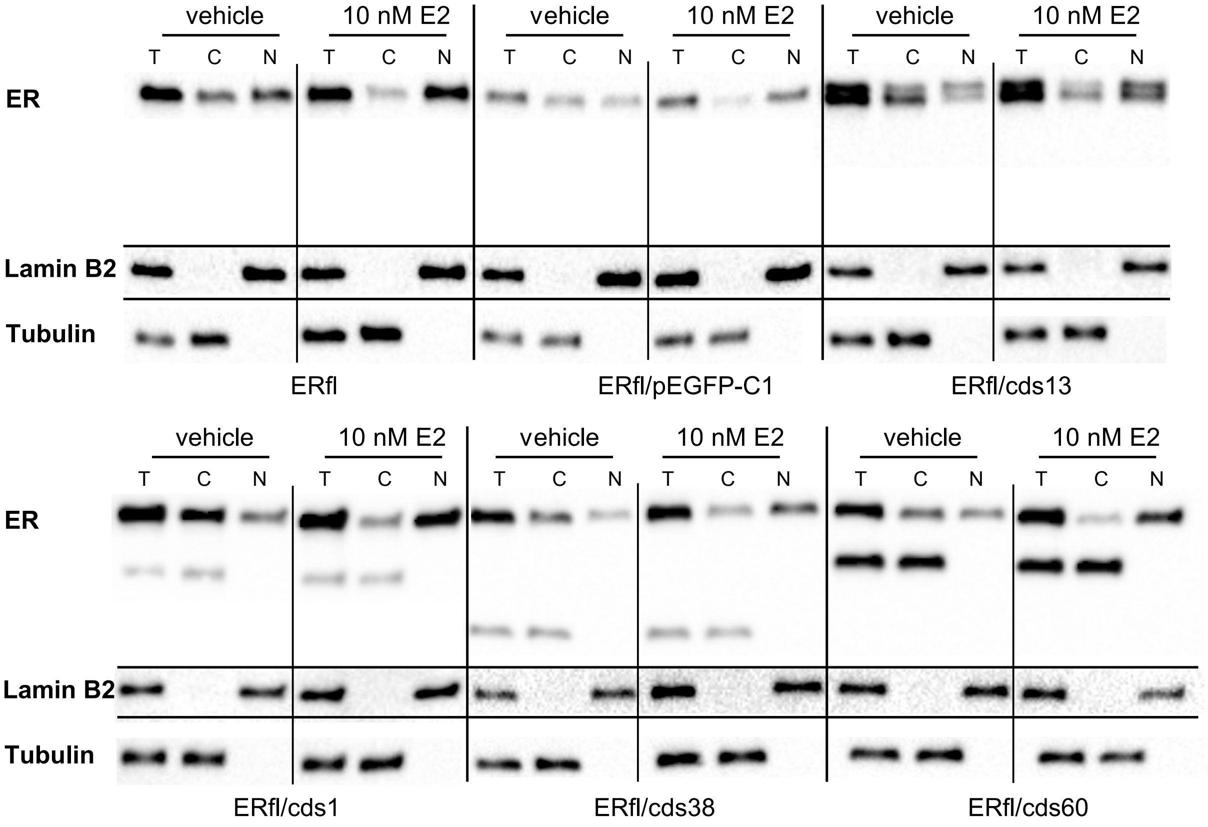

Supplementary Figure S24. Subcellular fractionation followed by western blotting using an N-terminal anti-ESR1 antibody to determine the relative distribution between cytoplasm and nucleus for full-length ER and co-transfected alternative isoforms after a 30 min treatment with vehicle or 10 nM E2. Lamin B2 and tubulin were included as controls for the purity of the nuclear and cytoplasmic fractions, respectively. Vehicle control = 0.1% ethanol, E2 = estradiol, ERfl = full-length estrogen receptor alpha (ER), pEGFP-C1 = empty pEGFP-C1 plasmid.

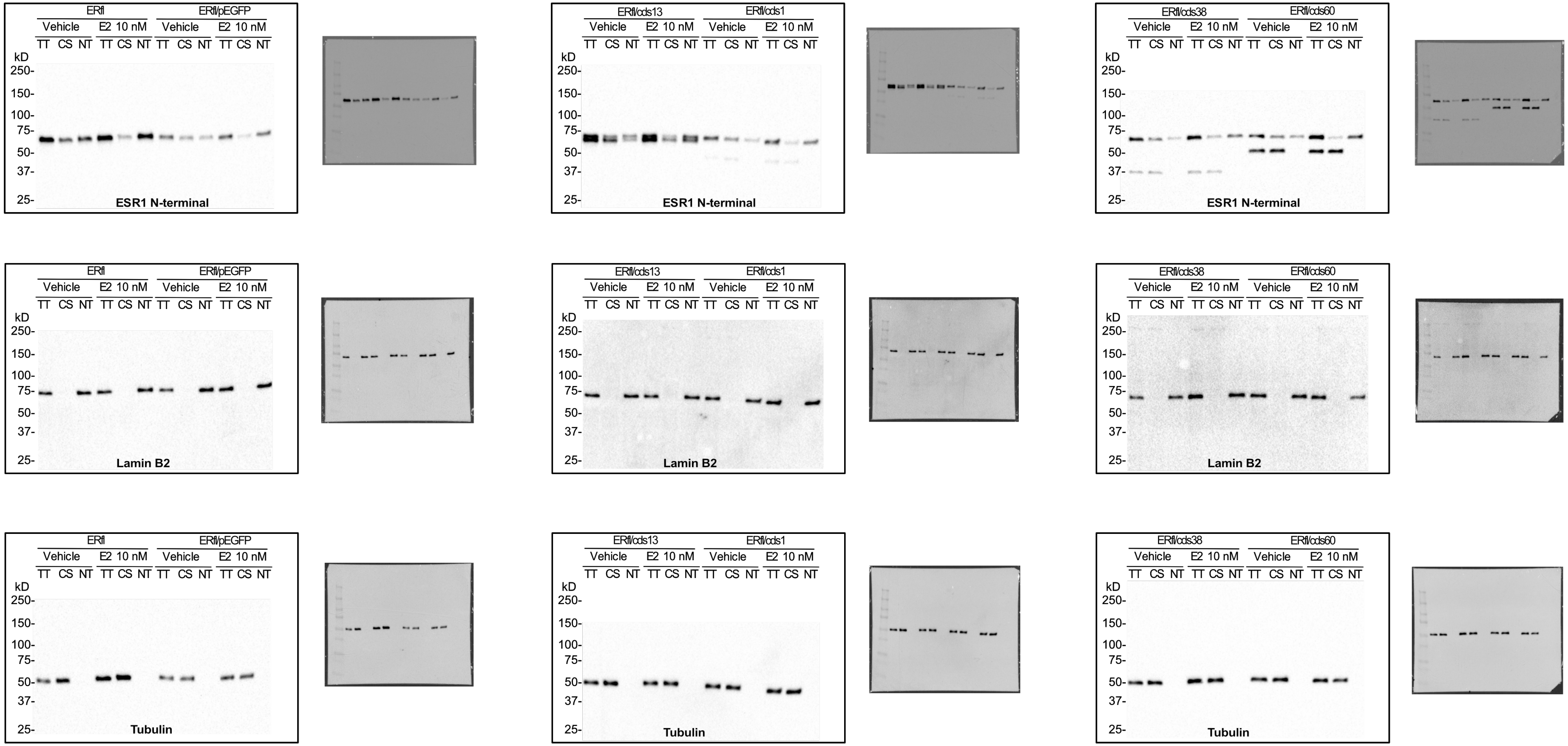

Supplementary Figure S25. Subcellular fractionation followed by western blotting to determine the relative distribution between cytoplasm and nucleus for isoform co-transfection; uncropped blots and overlay with size marker for subcellular fractionation experiments included in Supplementary Figure S19. Isoforms and antibody are marked in each panel.

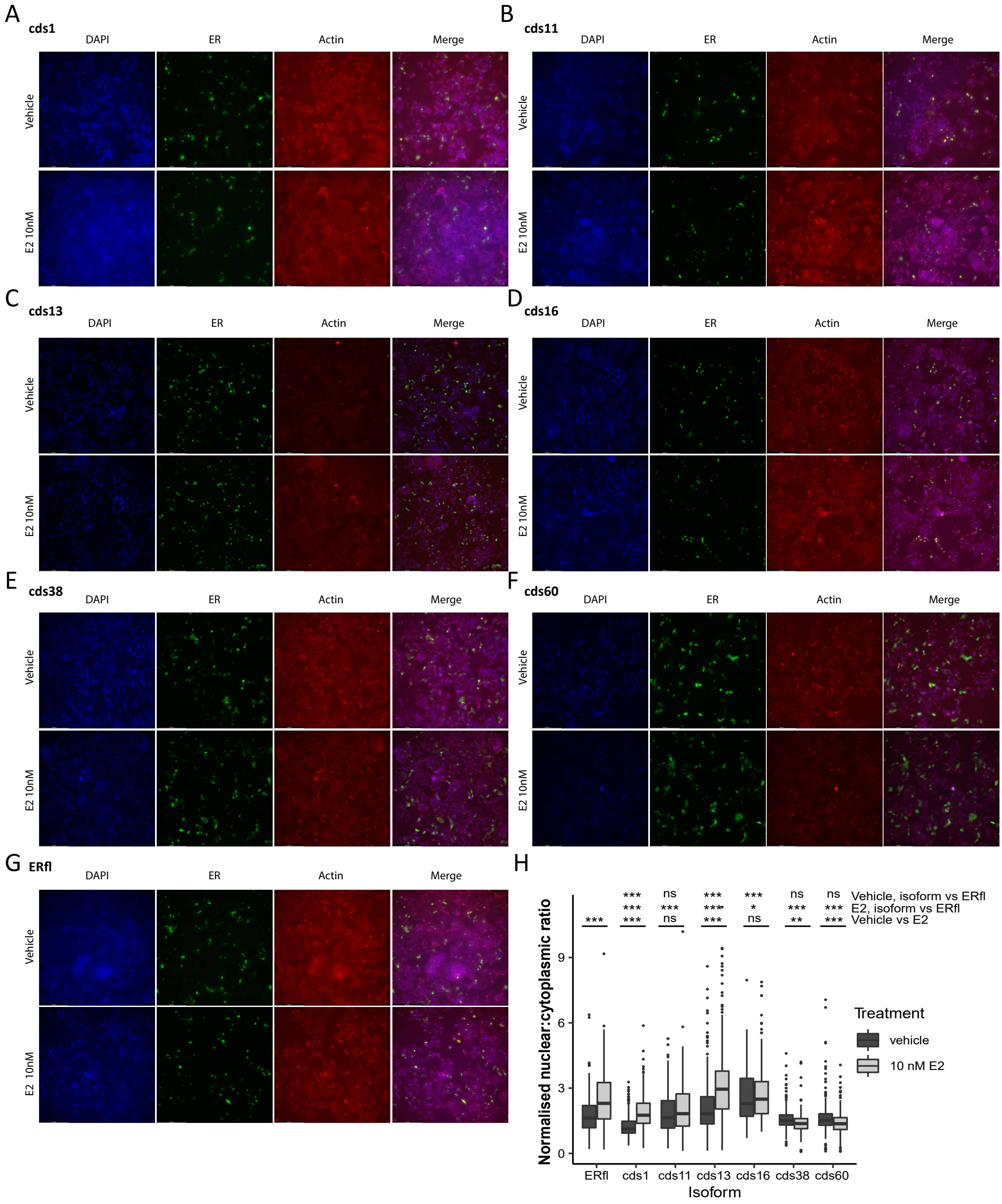

Supplementary Figure S26. Immunofluorescence images showing the subcellular localisation of alternative ER isoforms (A-F) and full-length ER (G) in HepG2 cells treated with 10 nM estradiol (E2) or vehicle control (0.1% ethanol) for 30 min. Quantitation of the ratio between nuclear and cytoplasmic intensity for ER normalised to actin (H). Boxplots include values for a mean of 270 cells (range 170-360 cells across isoforms and treatments) from n = 3 images. Two-tailed Student's *t*-test was used to test for significant differences between E2 and vehicle for each isoform, and for alternative isoforms compared with full-length ER, as indicated. * $p < 0.05$, ** $p < 0.01$, *** $p < 0.001$, and ns = not significant.

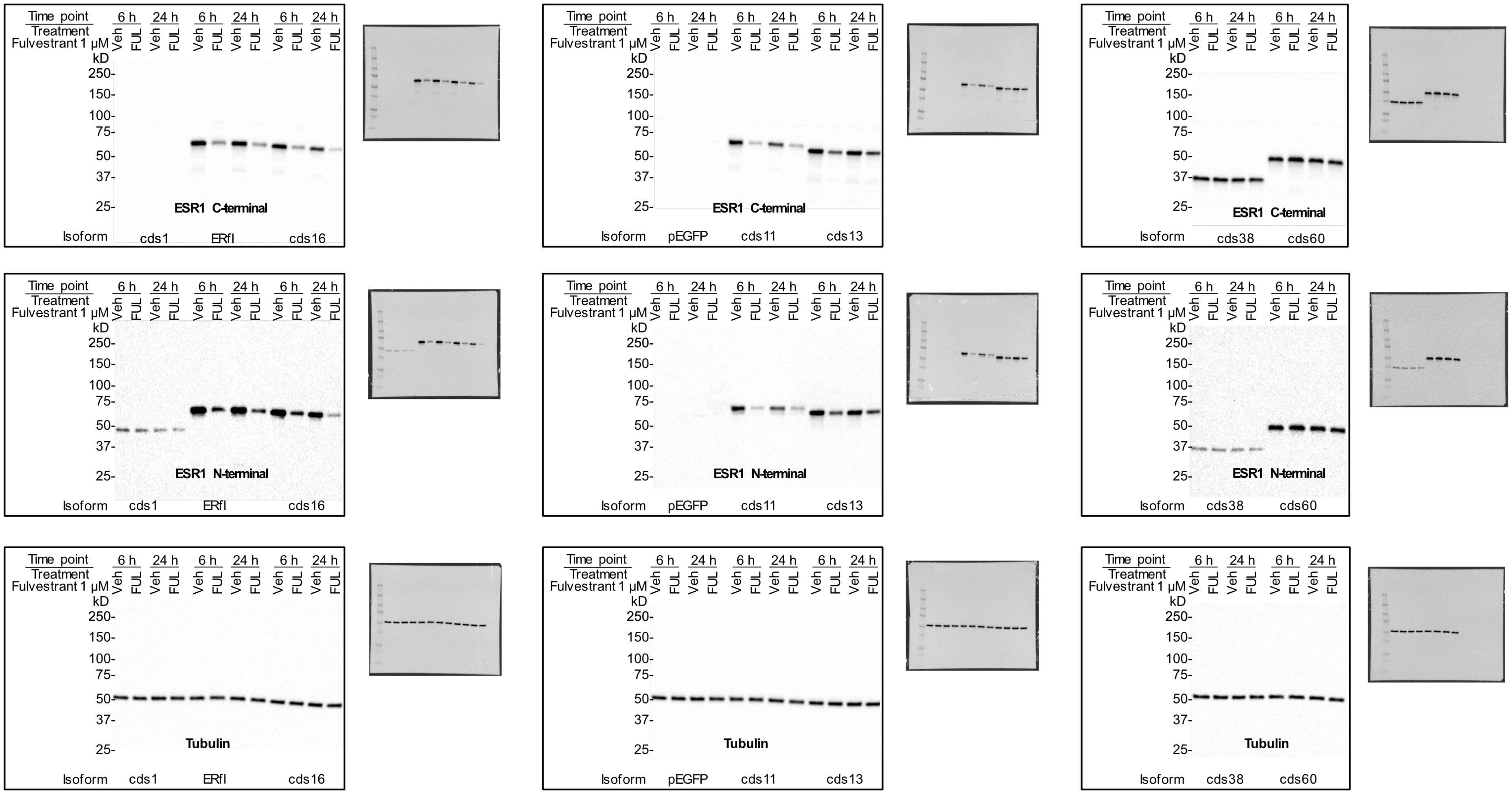

Supplementary Figure S27. Isoform sensitivity to treatment with fulvestrant; uncropped blots and overlay with size marker for fulvestrant treatment experiments included in Figure 10A. Isoform and antibody are marked in each panel.

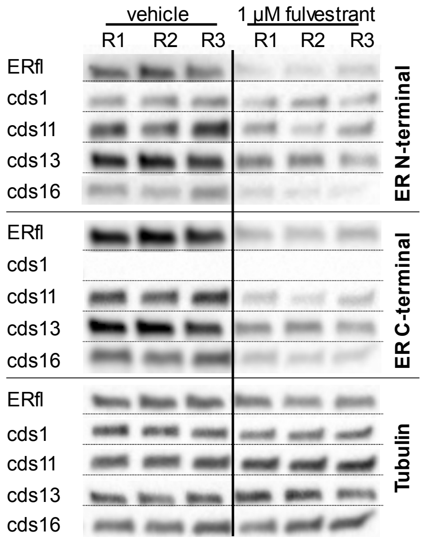

Supplementary Figure S28. HepG2 cells transfected in triplicate with plasmids encoding full-length ER or one of four alternative isoforms were treated with vehicle control (0.01% DMSO) or 1 µM fulvestrant for 24 h and ER expression was assessed by western blotting using N-terminal and C-terminal ER antibodies. Tubulin was included as a control for equal loading. ERfl = full-length estrogen receptor alpha (ER), R1-R3 = replicates 1-3.

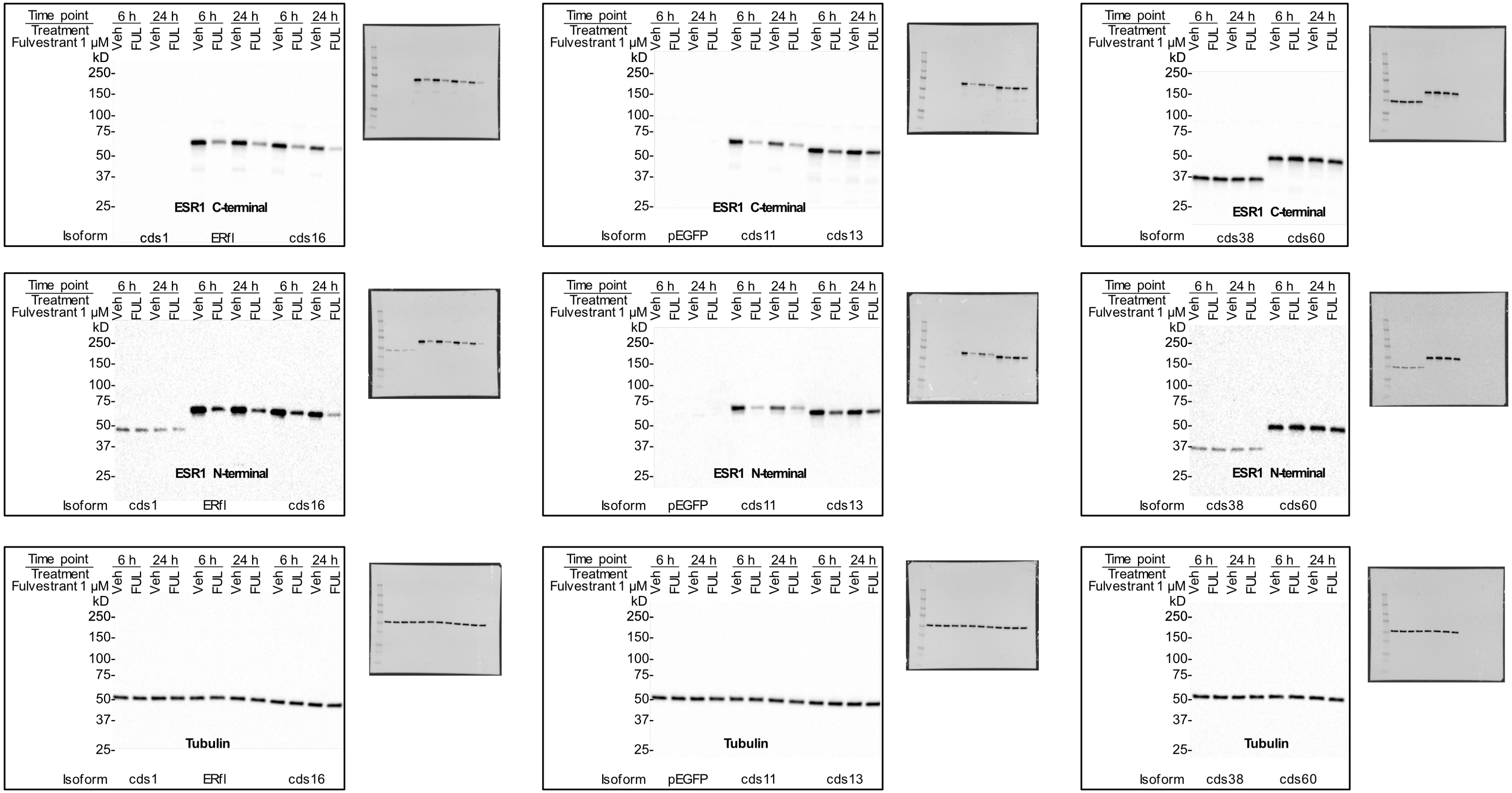

Supplementary Figure S29. Isoform sensitivity to treatment with fulvestrant with replicates; uncropped blots and overlay with size marker for fulvestrant treatment experiments included in Supplementary Figure S22. Isoform and antibody are marked in each panel.

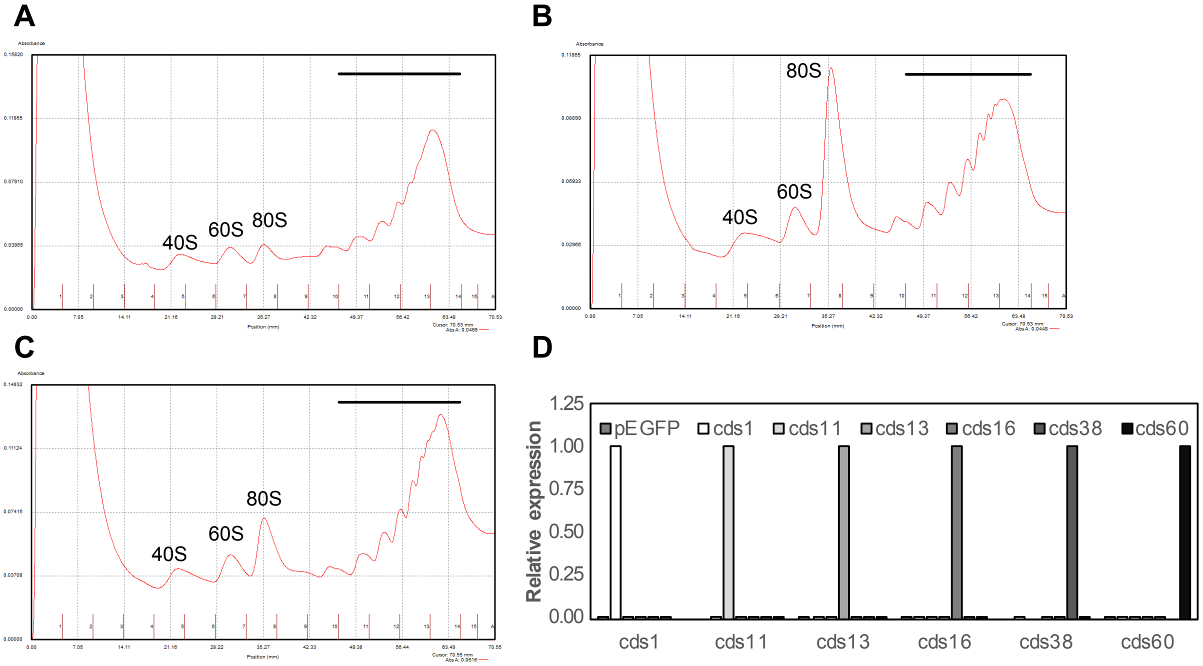

Supplementary Figure S30. Absorption curves for polysome fractionation for the (A) BT-474, (B) MCF7, and (C) T47D breast cancer cell lines. Black horizontal bars indicate the fractions used for purification of RNA from polysomes. The absorption peaks of 40S, 60S ribosomal subunits and 80S monosomes are labelled. (D) Validation of isoform-specific primers for cds1, cds11, cds13, cds16, cds38, and cds60 for real-time RT-PCR. ER-negative HepG2 cells were transfected with either empty pEGFP-C1 or pEGFP-C1 containing one of the six indicated isoforms. The specificity of each primer pair was assessed in all seven samples.

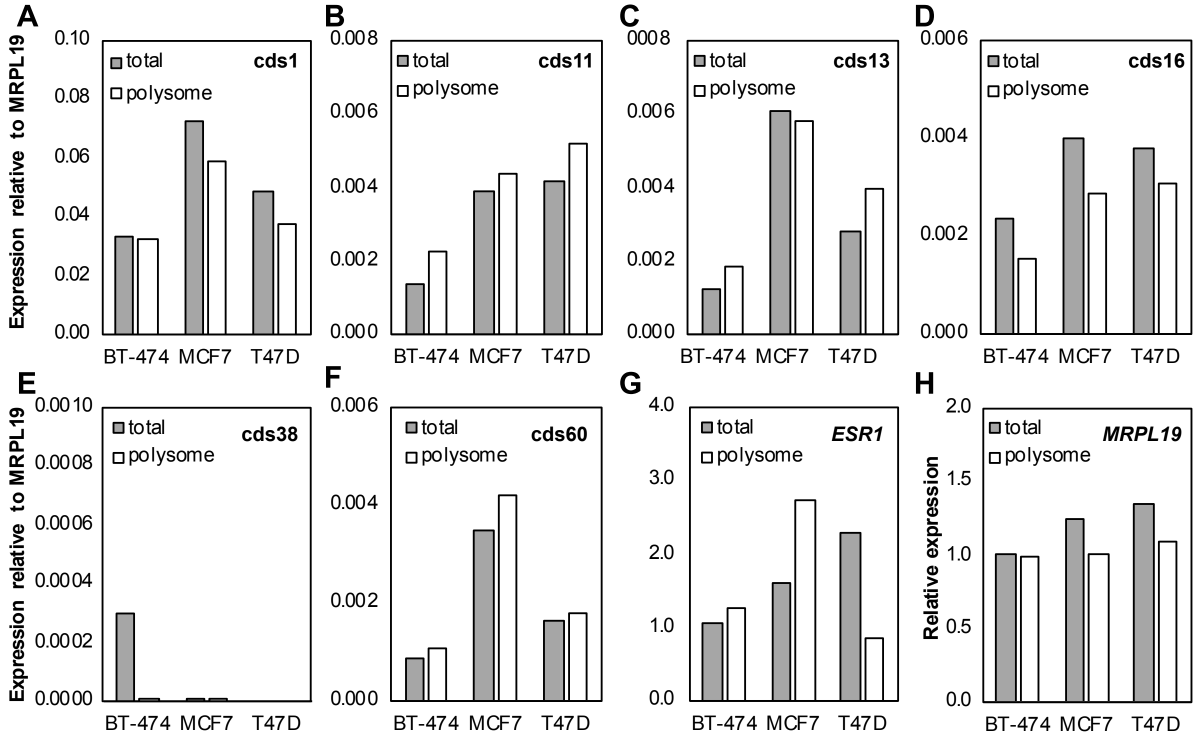

Supplementary Figure S31. (A-F) Detection of alternative isoforms in both total RNA and RNA isolated from translating ribosomes (polysome fraction) by isoform-specific real-time RT-PCR. Isoform names are indicated in each plot and expression levels are shown relative to expression of the reference gene *MRPL19* in each sample. (G) Total expression of all ER transcripts relative to *MRPL19* measured by amplification of a segment of their common 3' UTR. (H) Expression of the reference gene *MRPL19* in each sample relative to BT-474 total RNA.

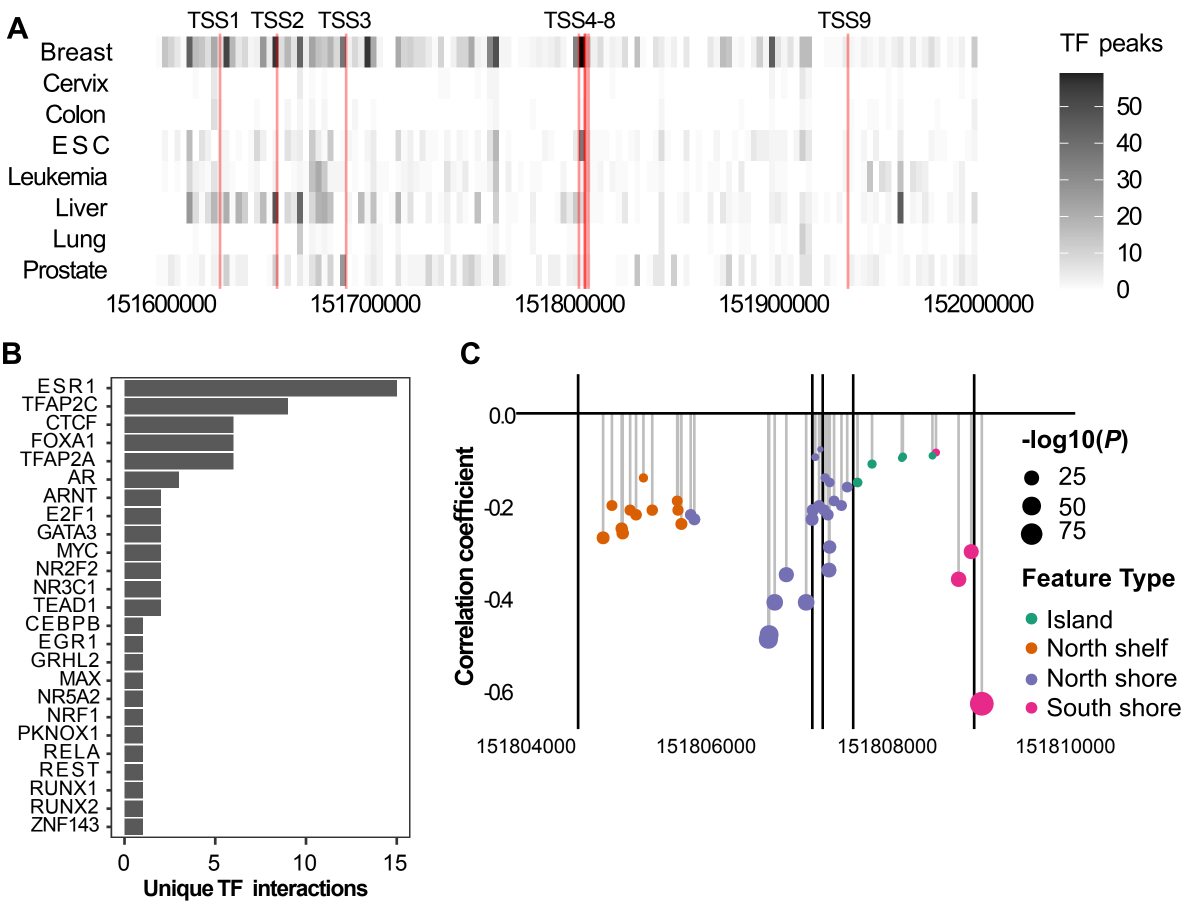

Supplementary Figure S32. (A) Number of unique transcription factor binding peaks per tissue of origin in 3 kb bins for a 400 kb region encompassing the *ESR1* transcription start sites (hg38 chr6:151,600,000-152,000,000) based on the UniBind robust set of transcription factor - DNA interactions. Red bars mark the positions of TSSs. (B) Number of unique peaks in the UniBind robust set per transcription factor in the proximal promoter region based on data for breast cancer cell lines. (C) CpG sites located in the proximal promoter region with significant negative correlation between the methylation beta value and ER expression in the TCGA breast cancer cohort. A total of 69 CpG sites with measured methylation levels were located within the analysed 400 kb region, most of them confined to an approximately 4 kb CpG island in the main promoter region. After correction for multiple testing, 49 out of the 69 CpG sites were significantly negatively correlated with expression of the ER.

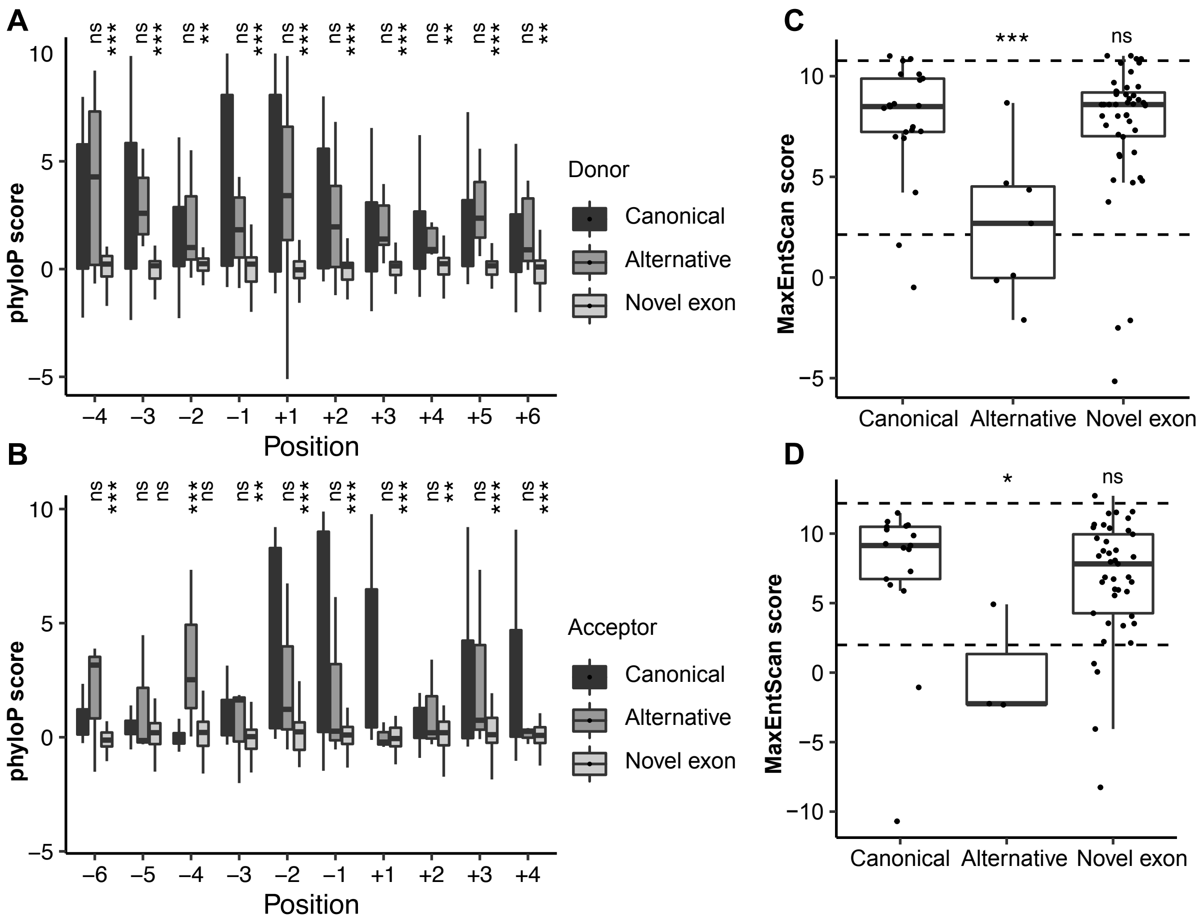

Supplementary Figure S33. (A, B) Evolutionary conservation in 100 vertebrate species from phyloP per position for *ESR1* splice donors and acceptors. The phyloP score is defined as the absolute value of $-\log_{10}(P)$ under a null hypothesis of neutral evolution. Positive scores correspond to conservation and negative scores imply accelerated evolution. For donors, -1 denotes the last base of the exon and +1 the first base of the intron. For acceptors, -1 is the last base of the intron and +1 is the first base of the exon. Donors and acceptors are divided into canonical splice sites in exons annotated in GENCODE and/or RefSeq, alternative splice sites in these exons, and splice sites in novel exons that are not annotated in either database. Both donor and acceptor sites show strong conservation of the three bases that include the intronic GU and AG motifs and the last and first bases of the exon, respectively. (C, D) Splice site strength predicted by MaxEntScan for donors and acceptors. Dashed lines mark the 5th and 95th percentiles of the calculated MaxEntScan scores for all unique splice sites in all protein-coding transcripts from GENCODE V39. Individual data points are included on top of the boxes. * $p < 0.05$, ** $p < 0.01$, *** $p < 0.001$, and ns = not significant.

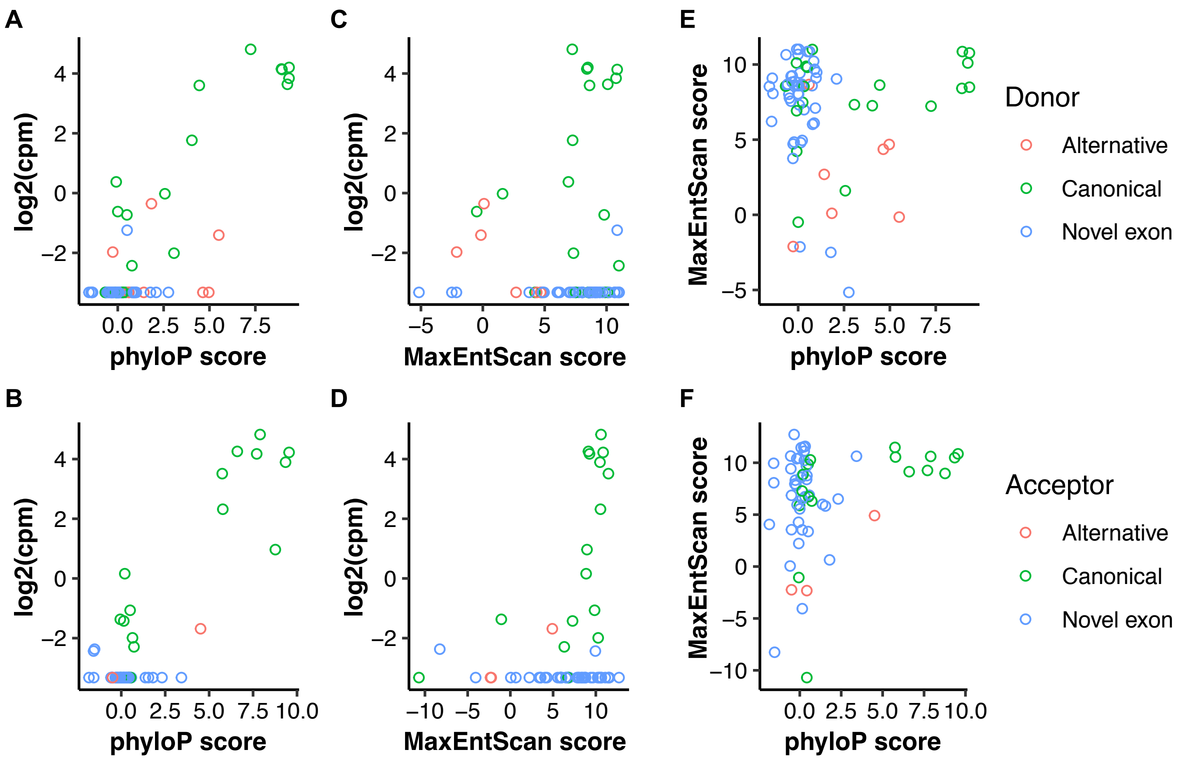

Supplementary Figure S34. (A, B) Median expression of individual *ESR1* splice donors and acceptors across the SCAN-B cohort in log_2-transformed counts per million reads (cpm) *versus* evolutionary conservation among 100 vertebrates from phyloP. Donors and acceptors are divided into canonical splice sites in exons annotated in GENCODE and/or RefSeq, alternative splice sites in these exons, and splice sites in novel exons that are not annotated in either database. The mean phyloP score for the three bases at the splice site (one exonic and two intronic) was used for plotting. (C, D) Expression of individual splice donors and acceptors *versus* splice site strength predicted by MaxEntScan. (E, F) MaxEntScan scores *versus* phyloP scores for splice donors and acceptors.
