# Supplementary material for: Alternative promoters and splicing create multiple functionally distinct isoforms of oestrogen receptor alpha in breast cancer and healthy tissues
Source: Cancer Med. 2023 Sep 7;12(18):18931–45. doi: 10.1002/cam4.6508 (PMC10557849; doi:10.1002/cam4.6508)
Supplement: Supplementary file 2 — Data S2. [file CAM4-12-18931-s002.pdf]

Alternative promoters and splicing create multiple functionally distinct isoforms of estrogen receptor alpha in breast cancer and healthy tissues

Carlos Enrique Balcazar Lopez, Juliane Albrecht, Völundur Hafstað, Cornelia Börjesson Freitag, Johan Vallon-Christersson, Cristian Bellodi & Helena Persson

Supplementary Methods

Patient data and short-read RNA-Seq

The SCAN-B study was conducted in accordance with the Declaration of Helsinki and has been approved by the Regional Ethical Review Board of Lund (2007/155, 2009/658, 2009/659, 2014/8), the county governmental biobank centre, and the Swedish Data Inspection group (364-2010). Written information was given by trained health professionals and all patients provided written informed consent. Library preparation and sequencing for RNA-Seq is described in [1].

Short-read RNA-Seq data analysis

The sequence data was made available by SCAN-B and had been aligned against GENCODE V27 and hg38 using HISAT2 2.1.0 [2]. Splice junction detection was done on BAM files using a custom Perl script that concomitantly calculates per-base sequencing depth and identifies and quantitates split-read alignments across all 3478 included samples. Known exons and splice junctions from GENCODE V36 and RefSeq release 109.20210226 were included in the preliminary annotation. The merged splice junction set was filtered to retain only junctions with an intron size above 70 bp (a calculated minimal intron size for human genes [3]), GU-AG intron ends and support from split reads in at least 10 breast tumour samples. This annotation set was used together with the sequencing read depth to guide manual curation of exons and splice junctions. Expression per splice junction was calculated from split reads as counts per

million reads (cpm) and for exons from all overlapping reads as fragments per kilobase of exon model and million reads (fpkm).

The R package complexHeatmap version 2.8 was used to plot expression values in cpm for splice junctions and fpkm for exons. Values were filtered to retain splice junctions with ≥2 cpm in ≥2 samples and exons with ≥3 fpkm in ≥3 samples and log_2-transformed. Spearman rank correlation analysis was done with the R packages stats version 4.1.2 and corrplot version 0.9 for the visualization of the correlation matrix. Percent spliced in (PSI) scores for differential expression analysis of alternative splicing events were calculated from spliced reads as PSI = I/(I + E), where I = overlapping spliced read pairs including a splice site or cassette exon and E = overlapping spliced read pairs excluding a splice site or cassette exon. Equivalent scores were calculated for first and last exons as PSI = I/(I + E), where I = spliced read pairs including a first or last exon and E = all spliced read pairs including any other first or last exon.

Long-read RNA-Seq

RNA was extracted with TRI Reagent (Sigma-Aldrich) from tumour samples provided by the SCAN-B tissue bank and the BT-474, MCF7, and T47D cell lines (purchased from ATCC) according to the manufacturer's instructions and treated with DNase I (Thermo Fisher Scientific). The FirstChoice Human Total RNA Survey Panel with normal tissue RNA was purchased from Ambion/Thermo Fisher Scientific. First-strand cDNA was generated using RevertAid H minus (Thermo Fisher Scientific) and an anchored oligo(dT) primer (Integrated DNA Technologies) as per the manufacturer's recommendations. Primers for reverse transcription polymerase chain reaction (RT-PCR) were designed to amplify full-length transcripts from seven different locations in first exons and are included in Supplementary

Table S1. PCR was done using AccuPrime GC-Rich DNA Polymerase (Thermo Fisher Scientific) with the following conditions: 98 °C for 3 minutes, followed by 34 cycles of 95 °C for 30 seconds, 55 °C for 30 seconds, 72 °C for 2 minutes, and a final extension step of 72 °C for 10 minutes. Products were purified using AMPure XP beads (Beckman Coulter) and quantitated with the Qubit dsDNA High Sensitivity Assay Kit (Thermo Fisher Scientific) before pooling all amplicons per sample at equimolar concentrations to a final mass of 150 ng (~140 fmol) per pool. Pools underwent end-repair with the NEBNext Ultra II End Repair/dA-Tailing Module (New England Biolabs) before clean-up with AMPure XP beads. Barcoding ligation was performed with the PCR Native Barcoding Expansion 1-12 PCR-free kit (Oxford Nanopore Technologies) and Blunt/TA Ligase Master Mix (New England Biolabs) to allow sample pooling and multiplexing. Barcoded amplicon pools were purified using AMPure XP beads and quantitated with the Qubit dsDNA High Sensitivity Assay Kit before pooling equimolar amounts of each barcoded sample (1-12) to reach a final concentration of 200 femtomoles. Library quality was assessed on a Bioanalyzer with a High Sensitivity DNA chip (Agilent Technologies) and measured using the Qubit dsDNA Broad Range Assay kit. Adapter ligation was performed using the Ligation Sequencing Kit (Oxford Nanopore Technologies), followed by purification using AMPure XP beads and elution in Elution Buffer. A primed flowcell (R9.4.1, Oxford Nanopore Technologies) was loaded with 50 femtomoles of the final pooled library in 75 µl (37 µl sequencing buffer, 25 µl loading beads, 5.5 µl DNA (50 fmol), and 6.5 µl water). The flowcell was sequenced on a GridION (Oxford Nanopore Technologies) for 20 hours.

Long-read RNA-Seq data analysis

Data acquisition was performed with MinKNOW and basecalling and demultiplexing were done using Guppy version 4.2.3. Reads were aligned using minimap2 version 2.17 with the

settings -ax splice -G 500k and --junc-bed together with the merged and filtered ER splice junction set. Full-length reads were identified and assigned to individual amplicons through comparison with the coordinates of forward and reverse primers used for RT-PCR. Transcripts were filtered to only retain isoforms with an intron size ≥70 bp and GU-AG intron ends and then divided into three tiers depending on the available support: Tier 1 isoforms only had exon ends present in the curated set of exons from SCAN-B breast tumours, GENCODE and RefSeq, tier 2 isoforms had one or more exon ends that were only present in the unfiltered SCAN-B junction set and tier 3 isoforms had one or more exon ends unsupported by new or existing annotation. Protein-coding isoforms were identified by scanning transcripts for start codons with open reading frames extending into the last exon to exclude likely candidates for nonsense-mediated mRNA decay. Open reading frames of tier 1 isoforms were translated into the corresponding amino acid sequences and compared to the full-length ER protein in a multiple sequence alignment [4]. Protein isoforms created by single alternative splicing events were identified and six were selected for functional studies.

Isoform and luciferase reporter cloning

The coding sequences of full-length ER and six selected alternative isoforms were ordered as double-stranded DNA fragments (Integrated DNA Technologies). To enable synthesis of these GC-rich sequences, each isoform was reconstructed from two fragments: A common 5' part and unique 3' sequences, all with flanking restriction sites as shown in Supplementary Table S2. The synthesised DNA fragments were then amplified by PCR before digestion of the 5' fragment with NheI and FseI, 3' fragments with FseI and BamHI, and the pEGFP-C1 vector (Clontech) with NheI and BamHI (all enzymes from New England Biolabs). The plasmid was dephosphorylated with FastAP (Thermo Fisher Scientific) before ligation of inserts and bacterial cloning. The isoforms cds11 and cds16 were created from the full-length

isoform construct with the QuikChange Site-Directed Mutagenesis kit (Agilent Technologies). The pmirGLO-3xERE reporter was constructed from the 3xERE-containing region of the 3xERE-TATA-luc plasmid [5] (Addgene) cloned in the pmirGLO vector (Promega) that had been engineered to introduce a multiple cloning site upstream of the transcription start site. The pmirGLO human C3 promoter reporter contains a weak ERE and an AP1 binding site (-307/+58, described in [6]) and was constructed by amplification of a fragment from a plasmid that was a kind gift from Gilles Flouriot, Research Institute for Environmental and Occupational Health, Rennes, France [7]. Plasmid preparations were made with the Plasmid Midi Prep kit (QIAGEN). All inserts were verified by Sanger sequencing. Primer sequences are shown in Supplementary Table S1.

Cell culture

Breast cancer cell lines were obtained from American Type Culture Collection (ATCC) and HepG2 from LGC Standards GmbH and cultured at 37 °C, 5% CO_2 in a humidified atmosphere. Cell line identity was authenticated by STR profiling and all cells were verified to be mycoplasma-free (Eurofins Genomics). HepG2 (RRID: CVCL_0027) and MCF7 (RRID: CVCL_0031) were cultured in Dulbecco's Modified Eagle's Medium (DMEM) High Glucose with 10% fetal bovine serum (FBS), both from Cytiva/HyClone. Insulin (Thermo Fisher Scientific) was added to 10 µg/ml for MCF7. BT-474 (CVCL_0179) and T47D (CVCL_0553) were cultured in RPMI-1640 (Cytiva/HyClone) with 10% FBS (Cytiva/HyClone). For experiments involving stimulation with ER ligands, cells were trypsinised, then washed and seeded in phenol red-free DMEM (Cytiva/HyClone) with 2.5% charcoal-stripped, dextran-treated FBS (CSS-FBS, Cytiva/HyClone).

Western blotting

Cells were lysed in RIPA buffer (1% Triton X-1000, 1% sodium deoxycholate, 10 mM Tris-HCl, pH 8.0, 1 mM EDTA, 0.5 mM EGTA 0.1% SDS, 140 mM NaCl; all Sigma-Aldrich/Merck) supplemented with cOmplete, Mini, EDTA-free Protease Inhibitor Cocktail (Roche/Merck) for all western blotting. Protein concentration was quantified using the Pierce BCA Protein Assay Kit (Thermo Fisher Scientific) according to the manufacturer's protocol and absorbance was measured on a FLUOstar Omega plate reader (BMG Labtech). Ten µg of each lysate were mixed with DTT (Thermo Fisher Scientific) to a final concentration of 0.1 M and diluted at a ratio of 4:1 with SDS-PAGE sample loading buffer (NuPAGE LDS Sample Buffer (4X), Invitrogen/Thermo Fisher Scientific) heated to 70 °C for 10 min and separated by SDS-PAGE on a 4–20% acrylamide gel (Mini-PROTEAN TGX Stain-Free Protein Gels, Bio-Rad). Precision Plus Protein All Blue Prestained Protein Standards (Bio-Rad) were used as molecular weight marker. Gels were run with 1x TGS buffer (Bio-Rad) at 180 V for 1 h. The gels were exposed to ultraviolet light (302 nm) for 60 sec and imaged with a ChemiDoc MP Imaging system (Bio-Rad). Proteins were blotted onto a PVDF membrane (Trans-Blot Turbo Mini 0.2 µm PVDF Transfer Packs, Bio-Rad) by electro-transfer at 2.5 A, 25 V for 7 minutes (Trans-Blot Turbo Transfer System, Bio-Rad) then the membrane was blocked in 5% non-fat dry milk in Tris-buffered saline with 0.1% Tween 20 (TBST) for 1 h at room temperature and washed in TBST. The C-terminal anti-ER antibody (sc-543, Santa Cruz Biotechnology) was diluted 1:500 and the N-terminal anti-ER antibody (HPA000449, Atlas antibodies) was diluted 1:250. The anti-α-tubulin antibody (ab7291, abcam) was used at a dilution of 1:5000 and the anti-Lamin B2 antibody (ab8983, abcam) at 1:1000. After washing in TBST, membranes were incubated in HRP-conjugated goat anti-rabbit (31460, Invitrogen/Thermo Fisher Scientific) or anti-mouse (31430, Invitrogen/Thermo Fisher Scientific) secondary antibodies. Signals were detected using Clarity Western ECL Substrate (Bio-Rad) and imaged on a ChemiDoc MP Imaging system (Bio-Rad). Bands were quantified

using the Image Lab Software Version 6.1.0 (Bio-Rad). Bound primary and secondary antibodies were removed using stripping buffer (Restore PLUS Western Blot, Thermo Fisher Scientific) and stored at 4 °C.

Luciferase assays

20,000 HepG2 cells were seeded per well in 96-well plates 24 h before transfection with empty pEGFP-C1 and/or ER isoforms cloned in pEGFP-C1 using jetOPTIMUS (Polyplus) as per the manufacturer's instructions. For MCF7 15,000 cells were seeded per well and Lipofectamine 2000 (Invitrogen/Thermo Fisher Scientific) was used for transfection as per the manufacturer's instructions. 24 h after transfection, the medium was replaced with fresh phenol red-free DMEM with 2.5% CSS-FBS containing either 0.1% ethanol (vehicle control), 10 nM E2 (Sigma-Aldrich/Merck), 10 nM E2 and 100 nM 4-hydroxytamoxifen (4-OHT, Selleck Chemicals), or 100 nM 4-OHT. Cells were lysed after 24 h in passive lysis buffer and analysed with the Dual-Luciferase Reporter Assay System (Promega).

Subcellular fractionation

300,000 cells were seeded per well in 12-well plates in phenol red-free DMEM with 2.5% CSS-FBS. Cells were transfected at 24 h with empty pEGFP-C1 and/or ER isoforms cloned in pEGFP-C1 using jetOPTIMUS as per the manufacturer's instructions. The medium was replaced with fresh phenol red-free DMEM with 2.5% CSS-FBS containing either 0.1% ethanol (vehicle control) or 10 nM E2 24 h after transfection. Cells were incubated at 37 °C for 30 min before scraping in subcellular fractionation buffer (ice-cold PBS with 0.1% NP-40 alternative (Calbiochem/Merck) supplemented with cOmplete, Mini, EDTA-free Protease Inhibitor Cocktail (Roche/Merck). An aliquot corresponding to 25 % of total protein was taken for the whole-cell lysate fraction and the remaining lysate was centrifuged briefly at

11,000 x g, 4 °C. An aliquot of the supernatant corresponding to 25% of the cytoplasmic fraction was taken. The remaining supernatant was discarded, and the nuclei-containing pellet was resuspended and washed with subcellular fractionation buffer before a brief centrifugation at 11000 x g, 4 °C, leaving a final pellet containing 75% of nuclear protein. All subcellular fractions were lysed in RIPA buffer supplemented with cOmplete, Mini, EDTA-free Protease Inhibitor Cocktail and analysed by western blotting as previously described.

Immunofluorescence

60,000 cells were seeded per well in phenol red-free DMEM with 2.5% CSS-FBS in Lab-Tek II Chamber Slides (Thermo Scientific/Nunc). After 24 h cells were transfected with ER isoforms cloned in pEGFP-C1 using jetOPTIMUS as per the manufacturer's instructions. At 24 h after transfection, cells were treated with vehicle control or 10 nM E2 for 30 min at 37 °C. Cells were washed with PBS, fixed for 10 min in 4% paraformaldehyde in PBS, and washed with PBS before permeabilisation for 5 min with 0.1% Triton X-100 in PBS. Cells were then washed with 0.1% Tween 20 in PBS (PBST) before blocking in 0.3M glycin, 0.1% Tween 20, 10% FBS in PBS for 1 h at room temperature. Incubation with primary antibodies (rabbit anti-ESR1, clone EP1, Dako, diluted 1:100 and mouse anti-actin, clone C4, MP Biomedicals, diluted 1:500 in blocking buffer) was done at 4 °C overnight. Slides were washed with PBST before incubation with secondary antibodies (AF488-conjugated goat anti-rabbit and AF647-conjugated goat anti-mouse diluted 1:1000 in blocking buffer, ThermoFisher/Invitrogen). Finally, slides were washed in PBST, incubated with DAPI in PBS for 15 min and washed again in PBST before mounting with ProLong Diamond Antifade Mountant (ThermoFisher/Invitrogen). Slides were imaged using a Leica DMi8 microscope. Image analysis was performed using CellProfiler 4.1.3.

ER degradation

150,000 cells were seeded per well in a 24-well plate in phenol red-free DMEM with 2.5% CSS-FBS 24 h before transfection with empty pEGFP-C1 and/or ER isoforms cloned in pEGFP-C1 using jetOPTIMUS as per the manufacturer's instructions. The medium was replaced with fresh phenol red-free DMEM with 2.5% CSS-FBS containing either 0.01 % DMSO (vehicle control, Thermo Fisher Scientific) or 1 µM fulvestrant (Selleck Chemicals) 24 h after transfection. Cells were incubated for 24 h then lysed in 35 µl RIPA buffer supplemented with cOmplete, Mini, EDTA-free Protease Inhibitor Cocktail and analysed by western blotting as previously described.

Polysome fractionation and isoform-specific real-time RT-PCR

Polysome fractionation was performed as described in [8] with minor modifications. At 48 h before harvest 3.5 million BT-474, MCF7 and T47D cells were seeded in 10-cm dishes. Cells were washed once with PBS and incubated for 10 min at 37 °C in medium supplemented with 10 µg/ml cycloheximide (Sigma-Aldrich). Cells were then placed on ice and washed twice with ice-cold PBS with 10 µg/ml cycloheximide before lysis in 600 µl passive lysis buffer containing 10 mM Tris-HCl pH 8.0, 150 mM NaCl, 1.5 mM $MgCl_2$, 0.25% NP-40 alternative (Calbiochem), 0.1% Triton X-100, 640 U/ml RiboLock RNase Inhibitor (Thermo Fisher Scientific), 150 µg/ml CHX cycloheximide, and 20 mM DTT. Cells were scraped on ice and lysates transferred to pre-chilled 1.5 ml tubes for incubation on ice for 40 min with intermittent vortexing. Lysates were cleared by centrifugation at 11,180 x g for 5 min at 4 °C and 300 µl of TRI reagent LS (Sigma-Aldrich) was added to 100 µl of supernatant for extraction of the total RNA fraction. Lysates were layered onto a linear sucrose gradient: 10%–60% sucrose (Thermo Fisher Scientific) (w/v), 25 mM Tris-HCl pH 7.4, 25 mM NaCl, 5 mM MgCl2, 0.1 mg/mL Heparin, 2 mM DTT in nuclease-free water and centrifuged in a SW41Ti rotor (Beckman

Coulter) at 37,000 rpm for 2.5 hr at 4 °C. Fractions were collected and polysome profiles generated using a BioComp Gradient Station (BioComp). Samples were stored on ice and 700 µl TRI reagent LS (Sigma-Aldrich) was added to each 650 µl fraction for RNA extraction with the Direct-Zol RNA Microprep kit (Zymo Research) according to the manufacturer's instructions including on-column DNase treatment. Fractions containing polysome RNA were pooled. 200 ng RNA was used for cDNA synthesis with anchored oligo(dT) primers ($dT_{20}VN$) in 10 µl reactions using RevertAid H Minus reverse transcriptase (Thermo Fisher Scientific) according to the manufacturer's instructions. The cDNA was diluted 1:3 with RNase-free H_2O and 2 µl were used in 15 µl reactions for real-time RT-PCR on the CFX96 real-time PCR detection system with iTaq Universal SYBR Green Supermix (Bio-Rad) according to the manufacturer's instructions. Primer sequences are included in Supplementary Table S1.

Analysis of regulation of promoters and alternative splicing

Transcription factor binding data were obtained from the UniBind robust set of transcription factor - DNA interactions [9]. For plotting we calculated the number of unique transcription factor binding peaks in each tissue type in 3 kb bins in a 400,000 base pair (400 kb) region surrounding the known and novel transcription start sites (TSS, hg38 chr6:151,600,000-152,000,000). For promoter methylation analysis *ESR1* expression and beta values were obtained for breast tumours (TCGA-BRCA) from The Cancer Genome Atlas (TCGA) [10]. The Pearson correlation coefficient was calculated for *ESR1* expression in $\log_2$-transformed fpkm against the $\log_2$-transformed beta values of CpG sites within the analysed 400 kb region. The Benjamini-Hochberg false discovery rate was used to correct for multiple testing.

For conservation analysis, phyloP [11] scores for the *ESR1* locus were obtained from the UCSC Table Browser [12] and matched to the positions of donors and acceptors using a Perl script. For

the MaxEntScan [13] web server calculations (http://hollywood.mit.edu/burgelab/maxent/Xmaxentscan_scoreseq.html and http://hollywood.mit.edu/burgelab/maxent/Xmaxentscan_scoreseq_acc.html), BED format coordinate files were first prepared with a Perl script to obtain splice site sequences in FastA format using the UCSC Table Browser, both for *ESR1* splice sites and for the background set of all unique splice sites in protein-coding transcripts from GENCODE V39.

Statistical analysis and data visualisation

Statistical analyses and plotting were done in R version 4.1.0 and Microsoft Excel. Survival analysis was performed in R using the survival and survminer packages. GraphPad Prism version 9 was used to plot the dose-response curve and calculate EC_{50} values. Genomic views of transcripts, exons, and splice junctions were created using custom tracks in the UCSC Genome Browser [14]. Figures were prepared with Affinity Designer 1.10.5.

Supplementary References

1. Saal LH, Vallon-Christersson J, Hakkinen J, Hegardt C, Grabau D, Winter C, Brueffer C, Tang MH, Reutersward C, Schulz R, Karlsson A, Ehinger A, et al. The Sweden Cancerome Analysis Network - Breast (SCAN-B) Initiative: a large-scale multicenter infrastructure towards implementation of breast cancer genomic analyses in the clinical routine. *Genome Med* 2015;**7**: 20.

2. Kim D, Paggi JM, Park C, Bennett C, Salzberg SL. Graph-based genome alignment and genotyping with HISAT2 and HISAT-genotype. *Nature biotechnology* 2019;**37**: 907-15.

3. Abebrese EL, Ali SH, Arnold ZR, Andrews VM, Armstrong K, Burns L, Crowder HR, Day RT, Jr., Hsu DG, Jarrell K, Lee G, Luo Y, et al. Identification of human short introns. *PloS one* 2017;**12**: e0175393.

4. Corpet F. Multiple sequence alignment with hierarchical clustering. *Nucleic acids research* 1988;**16**: 10881-90.

5. Hall JM, McDonnell DP. The estrogen receptor beta-isoform (ERbeta) of the human estrogen receptor modulates ERalpha transcriptional activity and is a key regulator of the cellular response to estrogens and antiestrogens. *Endocrinology* 1999;**140**: 5566-78.

6. Fan JD, Wagner BL, McDonnell DP. Identification of the sequences within the human complement 3 promoter required for estrogen responsiveness provides insight into the mechanism of tamoxifen mixed agonist activity. *Molecular endocrinology (Baltimore, Md* 1996;**10**: 1605-16.

7. Metivier R, Penot G, Flouriot G, Pakdel F. Synergism between ERalpha transactivation function 1 (AF-1) and AF-2 mediated by steroid receptor coactivator protein-1: requirement for the AF-1 alpha-helical core and for a direct interaction between the N- and C-terminal domains. *Molecular endocrinology (Baltimore, Md* 2001;**15**: 1953-70.

8. Guzzi N, Ciesla M, Ngoc PCT, Lang S, Arora S, Dimitriou M, Pimkova K, Sommarin MNE, Munita R, Lubas M, Lim Y, Okuyama K, et al. Pseudouridylation of tRNA-Derived Fragments Steers Translational Control in Stem Cells. *Cell* 2018;**173**: 1204-16 e26.

9. Puig RR, Boddie P, Khan A, Castro-Mondragon JA, Mathelier A. UniBind: maps of high-confidence direct TF-DNA interactions across nine species. *BMC genomics* 2021;**22**: 482.

10. Cancer Genome Atlas N. Comprehensive molecular portraits of human breast tumours. *Nature* 2012;**490**: 61-70.

11. Pollard KS, Hubisz MJ, Rosenbloom KR, Siepel A. Detection of nonneutral substitution rates on mammalian phylogenies. *Genome research* 2010;**20**: 110-21.

12. Karolchik D, Hinrichs AS, Furey TS, Roskin KM, Sugnet CW, Haussler D, Kent WJ. The UCSC Table Browser data retrieval tool. *Nucleic acids research* 2004;**32**: D493-6.

13. Yeo G, Burge CB. Maximum entropy modeling of short sequence motifs with applications to RNA splicing signals. *J Comput Biol* 2004;**11**: 377-94.

14. Kent WJ, Sugnet CW, Furey TS, Roskin KM, Pringle TH, Zahler AM, Haussler D. The human genome browser at UCSC. *Genome research* 2002;**12**: 996-1006.
